# Effects of Fluoride on Submandibular Glands of Mice: Changes in Oxidative Biochemistry, Proteomic Profile, and Genotoxicity

**DOI:** 10.3389/fphar.2021.715394

**Published:** 2021-09-27

**Authors:** Leidiane Alencar de Oliveira Lima, Giza Hellen Nonato Miranda, Walessa Alana Bragança Aragão, Leonardo Oliveira Bittencourt, Sávio Monteiro dos Santos, Michel Platini Caldas de Souza, Lygia S. Nogueira, Edivaldo Herculano Corrêa de Oliveira, Marta Chagas Monteiro, Aline Dionizio, Aline Lima Leite, Juliano Pelim Pessan, Marília Afonso Rabelo Buzalaf, Rafael Rodrigues Lima

**Affiliations:** ^1^ Laboratory of Functional and Structural Biology, Institute of Biological Sciences, Federal University of Pará, Belém, Brazil; ^2^ Laboratory of Clinical Immunology and Oxidative Stress, Faculty of Pharmacy, Institute of Health Sciences, Federal University of Pará, Belém, Brazil; ^3^ Section of Parasitology, Evandro Chagas Institute, Ananindeua, Brazil; ^4^ Laboratory of Cell Culture and Cytogenetics, Environment Section, Evandro Chagas Institute, Ananindeua, Brazil; ^5^ Department of Biological Sciences, Bauru School of Dentistry, University of São Paulo, Bauru, Brazil; ^6^ Department of Chemistry, University of Nebraska-Lincoln, Lincoln, NE, United States; ^7^ Department of Preventive and Restorative Dentistry, School of Dentistry, São Paulo State University (UNESP), Araçatuba, Brazil

**Keywords:** submandibular gland, fluoride, toxicity, proteome, genotoxicity

## Abstract

Although fluoride (F) is well-known to prevent dental caries, changes in cell processes in different tissues have been associated with its excessive exposure. Thus, this study aimed to evaluate the effects of F exposure on biochemical, proteomic, and genotoxic parameters of submandibular glands. Twenty one old rats (*n* = 30) were allocated into three groups: 60 days administration of drinking water containing 10 mgF/L, 50 mgF/L, or only deionized water (control). The submandibular glands were collected for oxidative biochemistry, protein expression profile, and genotoxic potential analyses. The results showed that both F concentrations increased the levels of thiobarbituric acid–reactive substances (TBARS) and reduced glutathione (GSH) and changed the proteomic profile, mainly regarding the cytoskeleton and cellular activity. Only the exposure to 50 mgF/L induced significant changes in DNA integrity. These findings reinforce the importance of continuous monitoring of F concentration in drinking water and the need for strategies to minimize F intake from other sources to obtain maximum preventive/therapeutic effects and avoid potential adverse effects.

## Introduction

Fluoride (F)-based strategies are well-known as effective, safe, and mainly responsible for the reduction of dental caries incidence, which has improved oral health and quality of life (Buzalaf et al., 2011). Whether present at low concentrations in the oral cavity, this ion inhibits tooth demineralization and enhances remineralization (Buzalaf et al., 2011; O'Mullane et al., 2016); thus, F is regularly added to the public water supply in several countries and some dental products to increase its systemic exposure (Buzalaf and Levy, 2011).

There is no evidence that fluoridation of drinking water at recommended concentrations causes systemic damage in humans (McDonagh et al., 2000). Despite the unquestionable benefits of F for caries control, evidence suggests that excessive exposure can affect human tissues in several ways, which depend on exposure time, concentration, and cell type (Iano et al., 2014). Metabolic, structural, and functional changes due to chronic exposure to high F levels have been observed in the liver, mitochondria, kidneys, small intestine, endothelial, neuronal, and gonadal cells; in addition, the development of the nervous system and reproductive skills have been jeopardized (Barbier et al., 2010; Iano et al., 2014; Dionizio et al., 2018; Pereira et al., 2018; Zuo et al., 2018; Araujo et al., 2019; Dionizio et al., 2020).

In this context, the literature claims that F can impair the metabolism of salivary glands, which are secretory organs that play a key role in the oral environment balance (Yamaguti et al., 2013). The parotids, submandibular, and sublingual glands are responsible for producing 90% of the total saliva, while the remaining 10% is produced by minor glands dispersed in the oral mucosa (Fernandes et al., 2015; de Paula et al., 2017). Saliva releases electrolytes, antibacterial compounds, and several enzymes that maintain oral homeostasis, lubricate the mucosa, and protect teeth surfaces (de Paula et al., 2017). The submandibular glands are composed of a mixed population of acini with mucous and serous functions (Amano et al., 2012; Fernandes et al., 2015; Kondo et al., 2015); thus, these glands secrete a unique fluid in the oral cavity that potentially has a specific function. Under unstimulated conditions, submandibular glands produce the major part (71%) of daily saliva (Armstrong and Turturro, 2013); in addition, proteolytic enzymes with antimicrobial functions such as lysozyme are also mainly produced by these glands (Noble, 2000).

Considering the importance of submandibular glands and the influence of F exposure on several tissues, this study aimed to evaluate the effect of this ion on biochemical, proteomic, and genotoxic parameters of submandibular glands.

## Materials and Methods

### Animals and Experimental Protocol

The experimental protocol of this study was submitted to and approved by the Ethics Committee on the Use of Animals (CEUA) of the Federal University of Pará, under the protocol number 9469260117. Thirty male Swiss albino mice, 21 days old, were randomly divided into three groups of 10 animals each. The mice were housed in collective polypropylene cages containing five animals each, with water and food ad libitum, maintained in a light/dark cycle of 12 h (lights on at 7 a.m.).

The protocol of F exposure was based on the administration through the drinking water for 60 consecutive days, at 10 and 50 mgF/L (as sodium fluoride, Sigma-Aldrich, United States), while the control group received pure deionized water. This protocol mimics long-term F intake by humans at concentrations that correspond to approximately 1–2 and 5–10 mgF/L (Dunipace et al., 1995; Dionizio et al., 2018; Miranda et al., 2018). Such concentrations lead to plasma F levels in rodents similar to those found in humans who consume artificially fluoridated water or live in endemic areas of fluorosis, considering the difference of metabolism between both species (5–10-fold higher in rodents than in humans) (Dunipace et al., 1995).

### Collection of Submandibular Glands

**FIGURE 1:**
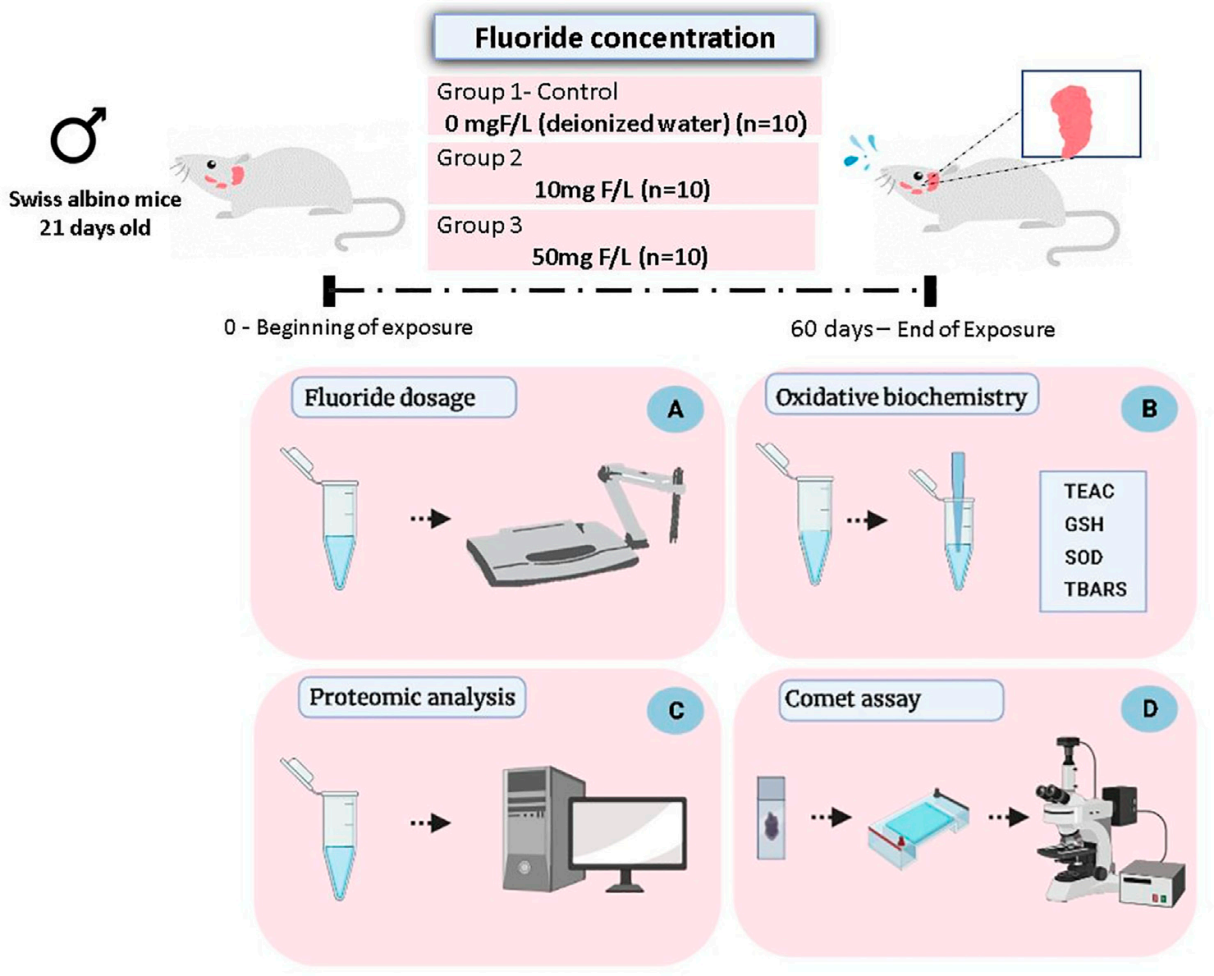
Methodological steps of animal experimentation, sample collection, and analyses. Male Swiss albino mice (21 days old) received drinking water at two concentrations of F (10 or 50 mgF/L) while the control group received only deionized water, for ad libitum consumption. After 60 days of exposure, the animals were euthanized and the pair of submandibular glands was collected for the following analyses: quantification of the fluoride levels in the glandular tissue **(A)**; evaluation of oxidative biochemistry **(B)**, based on the Trolox equivalent antioxidant capacity (TEAC), activity of the superoxide dismutase enzyme (SOD), reduced glutathione (GSH) levels, and thiobarbituric acid–reactive substances (TBARS) levels; proteomic analysis **(C)**; and evaluation of genotoxicity based on the Comet assay **(D)**.

After the experimental period (60 days), the animals were anesthetized with ketamine hydrochloride (90 mg/kg) and xylazine hydrochloride (10 mg/kg), being subsequently euthanized by cervical dislocation. Then the pair of submandibular glands was collected, washed with saline solution, and frozen in liquid nitrogen, followed by storage at −80°C until biochemical, proteomic, and genotoxic analyses (Figure 1).

### Assessment of Fluoride Levels

Approximately 0.2 g of glandular tissue was homogenized in 0.5 ml ultrapure water and had the CO_2_ removed by the addition of hexamethyldisiloxane (HMDS). Then, the F measurements in the tissue were performed, as described by Taves (1968) and modified by Whitford (1996), using an F-specific electrode (Orion, model 9409) and a reference calomel electrode (Accumet, # 13-620-79), both coupled to a potentiometer (Orion model EA 940). The standard solutions used in the calibration curve (0.0048–0.19 μgF) were prepared in triplicate and used in the same way as the samples.

In addition, non-diffused standards were prepared to have exactly the same concentrations of F as the diffused standards. The readings (mV) were converted to µg F using an Excel (Microsoft) spreadsheet. A standard curve was adopted with a coefficient correlation of r ≥ 0.99. The results were expressed in µg F/ml.

### Oxidative Biochemistry Analyses

Each sample was thawed and resuspended in Tris-HCl (20 mM, pH 7.4 at 4 °C) for sonic homogenization (approximate concentration of 1 g/ml). The supernatants were stored in an ultra-freezer until analysis.

#### Determination of Total Antioxidant Capacity (TEAC)

The method proposed by Miller et al. (1993), and modified by Re et al. (1999), was adopted, which consists in a colorimetric technique based on the reaction between 2,2-azino-bis(3-ethylbenzothiazoline)-6-sulfonic acid (ABTS) with potassium persulfate, producing the ABTS^+^ radical, being subsequently incubated with the sample or the Trolox standard. A total of 30 µl of supernatant of the submandibular glands or standard were incubated with 2,970 µl of ABTS for 5 min, and the absorbance was read at 734 nm. The results were expressed in μmol/g and then expressed as % of the control group, after extrapolation with the standard curve.

#### Reduced Glutathione (GSH) levels

The determination of GSH concentrations was performed according to Ellman (1959). This technique is based on GSH’s ability to reduce 5,5-dithiobis-2-nitrobenzoic acid (DTNB) (Sigma-Aldrich) to 5-thio-2-nitrobenzoic acid (TNB), which is quantified by spectrophotometry at a wavelength of 412 nm. First, an aliquot of 20 μl was removed from each of the submandibular glands and placed in a test tube containing 3 ml of PBS/EDTA buffer and 20 μl of distilled water for the first sample reading (T_0_), then 100 µl DTNB (0.47 mmol) was incubated for 3 min to perform another reading (T_1_). The difference in absorbances (T_1_–T_0_) is proportional to the concentration of GSH, which was expressed in μM/g and then converted to % of the control group.

#### Determination of Superoxide Dismutase (SOD) Activity

This assay was performed according to McCord and Fridowich (1969). An aliquot of 50 µl of the submandibular glands was added to a solution composed of cytochrome C (0.075 mM), hypoxanthine (1.5 mM; Sigma-Aldrich, United States), and xanthine oxidase (56 mM; Sigma-Aldrich, United States). The resulting solution was incubated at 37°C, protected from light, and after 15 min, a single reading was performed at 550 nm. This activity was measured using UV spectrophotometry at a wavelength at 550 nm wavelength (Miranda et al., 2018). The activity of the SOD enzyme was expressed in nmol/mg and then converted to % of the control group.

#### Analysis of Thiobarbituric Acid-Reactive Substances (TBARS)

The determination of lipid peroxidation was performed by the method adapted by Percário (1994). An aliquot of 50 µl of the submandibular glands was added to 500 µl of thiobarbituric acid solution (10 nM) and then heated in a water bath at 94 °C for 60 min. Then, the samples were left at room temperature for 10 min, and 2 ml of 1-butyl alcohol was added, vigorously homogenized in a vortex, and centrifuged at 2,500 rpm for 10 min. After centrifugation, 1 ml of the supernatant was collected for spectrophotometric reading at 535 nm. The MDA standard was used for the standard curve; the results were expressed in nmol/g and then converted into % of the control group.

### Proteomic Analysis

The samples of submandibular glands were initially cryofractured with liquid nitrogen and then, two samples from the same group were pooled and all the experiment was proceeded in biological triplicate. The detailed protocol is described in previous publications from our group (Bittencourt et al., 2019; Alves Oliveira et al., 2020; Dionizio et al., 2020; Leão et al., 2020; Lopes et al., 2020; Ferreira et al., 2021).

The first step consisted of protein extraction by lysis buffer containing 7 M urea, 2 M thiourea, and 40 mM dithiothreitol (DTT) in a 50 mM ammonium bicarbonate solution (AmBic) under constant stirring at 4 °C for 2 h. Then, the samples were centrifuged (20,817 g, 30 min, 4 °C) and the supernatant was collected, and the protein content was determined by Bradford’s method (Bradford, 1976). We used 50 µg of protein and then filled it up with AmBic until 50 µl. Then, in the second step, the samples were incubated with RapiGEST (Waters Co., United Kingdom), reduced in 5 mM DTT (Bio-Rad, United States), alkylated in 10 mM iodoacetamide (Bio-Rad, United States), and digested by trypsin (Pierce®, Thermo Fisher, United States). Next, the digestion was stopped using 5% trifluoroacetic acid and incubated under 37°C for 90 min; in the end, samples were centrifuged (20,817 g, 30 min, 6°C), the supernatant was collected for desalting through C18 spin columns (Pierce®, Thermo Fisher, United States), and subsequently concentrated by a vacuum concentrator (Eppendorf, Germany).

To perform the mass spectrometry readings (nanoAcquity UPLC-Xevo QTof MS system; Waters Co., United Kingdom), subsequently, the samples were resuspended in 108 μl of 3% acetonitrile and 0.1% formic acid and 12 μl of internal standard. The peptides identification and statistical analyses were performed by ProteinLynx Global SERVER (PLGS) software, after downloading and comparing the *Mus musculus* proteome from the UniProt database.

#### Bioinformatic Analyses

The proteins were identified according to their respective UniProt Accession ID, and the functional analyses were performed by Cytoscape 3.7.1 software. The biological processes were listed according to the Gene Ontology annotations provided by ClueGO plugin in Cytoscape (Bindea et al., 2009).

For an over-representation analysis (ORA), the fold change values were converted to log2ratio using the WPS spreadsheets editor. Then, cutoff values were applied for screening proteins with an expression value of 50% above or below in the exposed condition compared to the control. The analysis was performed considering only proteins with log2ratio values ≤ −0.58 or ≥0.58. The values −1 were assigned to proteins detected only in the control group and 1 to those presented exclusively in the exposed sample.

Using the R program and the Gene Set Enrichment Analysis (EGSEA) package (Alhamdoosh et al., 2017) and the list of Entrez IDs and their respective log2ratio values as input, an ORA analysis was performed. A database with UniProt information on proteins and biological processes was used, made available by the Bader lab. The *p*-value ≤0.05 was considered significantly altered. Then, to perform the network analysis from the list of proteins with significant statistical changes, networks and clusters were built to visualize their interconnections. The selection process was based on the relationship between the different proteins and their interactions with the others that were found altered, establishing a cut of at least 10 interactions.

The Cytoscape 3.7.1 program (Shannon et al., 2003), the Enrichment map (Merico et al., 2010), and the AutoAnnotate (Kucera et al., 2016) app were used. The online tool NetworkAnalyst was used to analyze the interaction between the sorted proteins (Xia et al., 2014). These were ranked based on their degree values and are therefore the most relevant proteins.

### Genotoxic Evaluation

A DNA damage assay was analyzed using the single-cell gel electrophoresis (SCGE) alkaline comet assay following the method described by Singh and Stephens (1997). Glandular tissue was dissected from the control and exposed animals (10 and 50 mgF/L). The tissues were prepared to isolate the cells by slicing into small pieces and incubated with type IV collagenase diluted in DMEM cell culture medium (Dulbecco’s modified Eagle’s medium) at 37°C for 90 min. Then, the samples were filtered to remove debris and centrifuged for 5 min at 2000 rpm. The pool of cells formed was resuspended with 150 µl of Dulbecco’s modified Eagle’s medium. An aliquot (20 µl) was homogenized with a low melting point agarose (120 µl) and added to the glass slides previously pretreated with normal 1.5% melting point agarose. Coverslips were added in each slide and stored at 4 °C for 20 min. Following that, the coverslips were removed and the slides were incubated in a lysis solution (2.5 M NaCl, 0.1 M EDTA, 0.01 M Tris, and 1% Triton X-100) overnight at 4 °C. Longer lysis incubation provides not only an increase of the sensitivity of the test based on effective disruption of cellular membrane but also the cytoplasm, nuclear envelope, scaffold, and histone proteins around the DNA (Karbaschi et al., 2019; Olive et al., 2002). After incubation, the slides were placed in an electrophoresis vessel with electrophoresis solution (300 mM NaOH, 1 mM EDTA; pH 13) for 20 min for the unwinding of the DNA. The electrophoresis was performed at 300 mA and 30V (1 V/cm) for 20 min. The slides were then neutralized with 0.4 M Tris buffer (pH 7.5) and fixed in 100% ethanol. Slides were stained with DAPI/antifade (Enzo Life Sciences, NY, United States) and images were captured through a fluorescence microscope (Leica Microsystems, Wetzlar, Germany, with 400x magnification) connected to a CCD camera (from an English charge-coupled device). Fifty nucleoids from two different slides per animal were analyzed using Komet Assay software®.

Cells in which DNA was damaged demonstrated increased chromosomal migration (Speit and Hartmann, 1999); as a result, the parameter used for determining DNA damage was the percentage of DNA in the tail comet.

### Statistical Analyses

The normality was initially tested by the Kolmogorov–Smirnov method. Data of F concentration, oxidative biochemistry, and genotoxic evaluation were analyzed using one-way analysis of variance (ANOVA), followed by Tukey’s post hoc test, considering a significant value of *p* < 0.05. Proteomics data analysis was performed using the ProteinLynx Global SERVER software (PLGS, v 2.2.5, Waters), and the significance of the relative expression ratios was calculated using a *t* test (*p* < 0,05).

## Results

### There was a Significant Increase in F levels in the Submandibular Gland Only in the Group that Received 50 mgF/L Drinking Water

**FIGURE 2:**
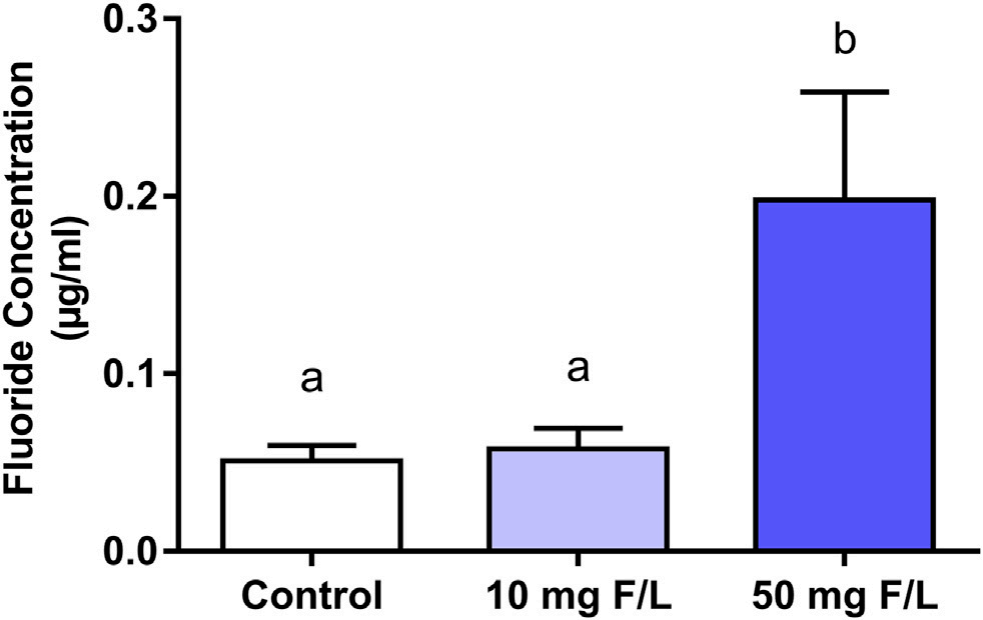
Fluoride levels in the submandibular gland of mice. One-way ANOVA with Tukey’s posttest, *p* < 0.05. Lowercase letters indicate significant differences among the groups.

Fluoride levels present in the submandibular gland of the group that received the highest exposure dose were significantly higher than the levels of the control group (control: 0.05 ± 0.01 µg/ml; 50 mgF/L: 0.19 ± 0.12 µg/ml; *p* = 0.0062). No significant difference was observed when the 10 mg F/L group (0.05 ± 0.03 µg/ml) was compared with the control group (*p* > 0.05) (Figure 2).

### Fluoride Exposure Modulated the Oxidative Biochemistry Profile of Submandibular Glands of Mice

**FIGURE 3:**
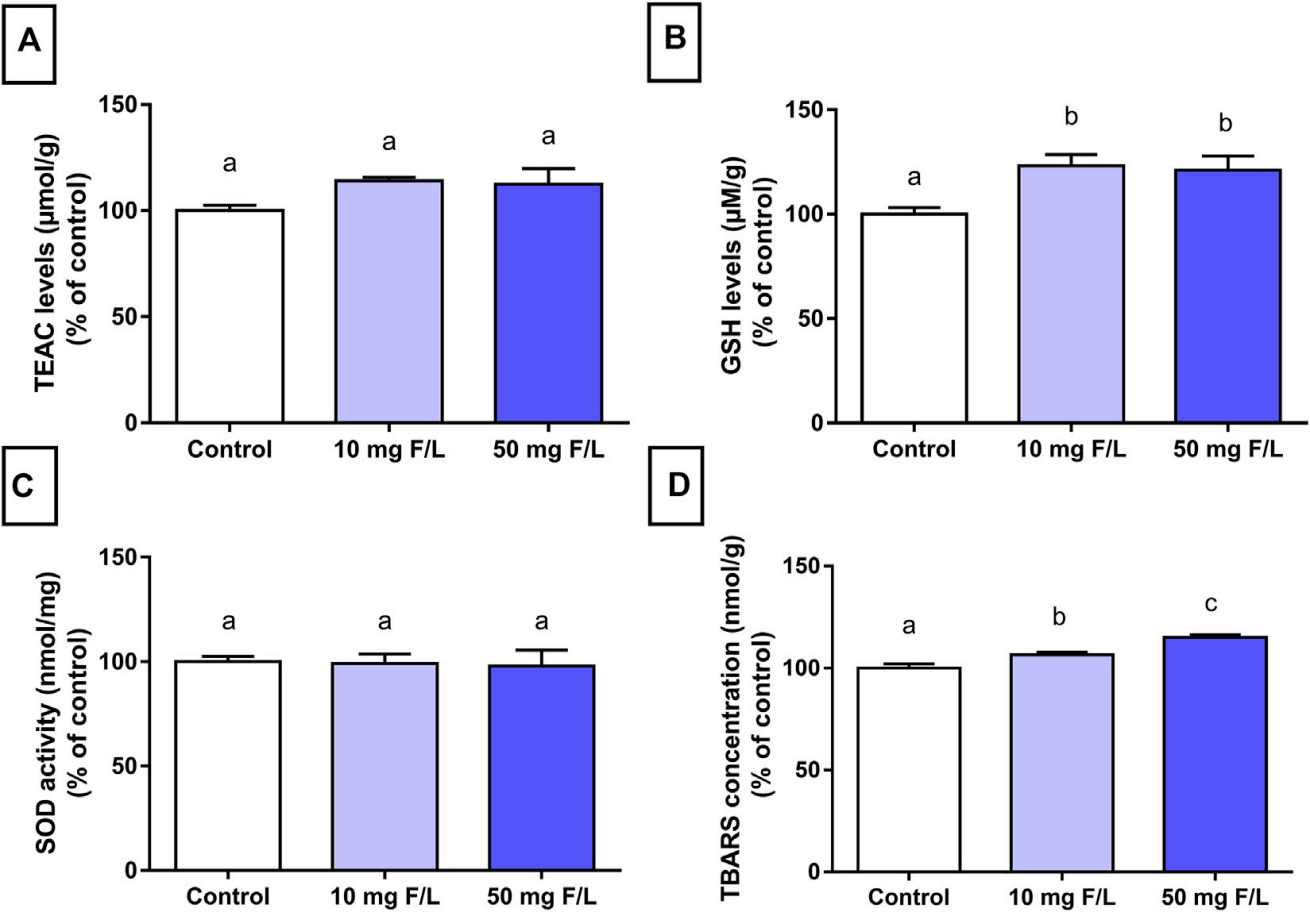
Evaluation of oxidative biochemistry. Analysis of oxidative biochemistry in the submandibular glands of mice that received drinking water containing 0, 10, and 50 mgF/L, during 60 days. The graphs represent, as a relative percentage of the control group, the mean and the standard deviations of the following oxidative parameters: **(A)** TEAC levels; **(B)** GSH levels; **(C)** SOD activity; and **(D)** TBARS concentration. One-way ANOVA and Tukey’s post hoc test, *p* < 0.05. Different lowercase letters indicate significant differences among the groups.

The antioxidant capacity shown by GSH levels was significantly increased in both groups exposed to F (*p* = 0.0123) compared to the control group. No significant difference was observed in TEAC levels and SOD activity (*p* > 0.05). On the other hand, there was a minimal difference between both exposed groups compared to the control (*p* < 0.0001; Figure 3).

### Long-Term Exposure to F Modulated the Global Proteomic Profile of Submandibular Glands of Mice

The number of proteins identified with significant differences in expression, or exclusively expressed, considering comparisons between 10 mgF/L vs. control, 50 mgF/L vs. control, and 10 mgF/L vs. 50 mgF/L, is presented in Table 1. The complete proteomic data of this study are available on the Supplementary Tables S1–S3.

**TABLE 1:** Number of proteins with different status of regulation in submandibular glands of mice exposed to fluoride (F).

Comparison	Upregulated	Downregulated	Exclusive in the first group	Exclusive in the second group
10 mgF/L *vs.* control	248	30	291	244
50 mgF/L *vs.* control	68	146	212	290
50 mgF/L *vs.* 10 mgF/L	15	346	316	202

**FIGURE 4:**
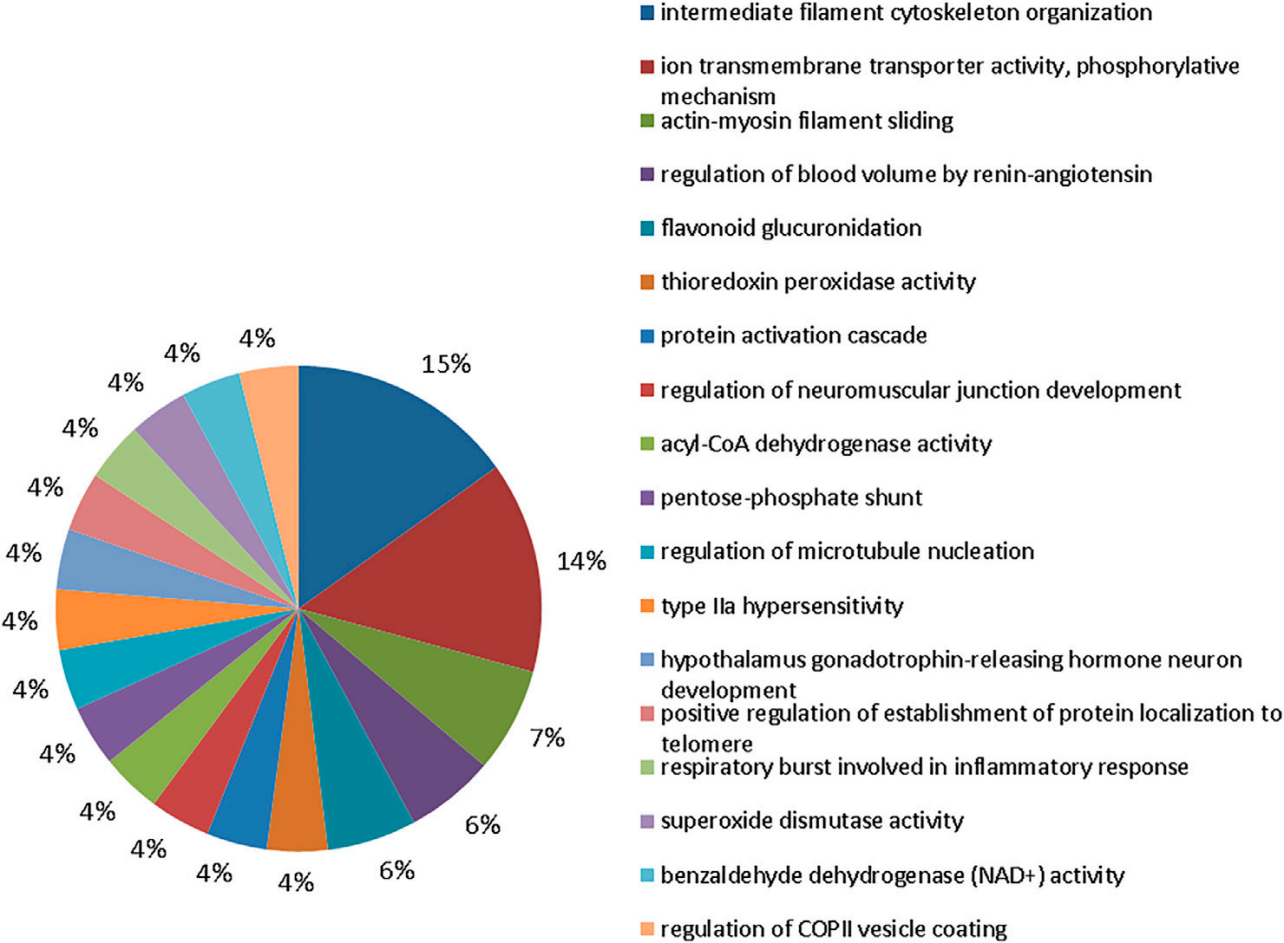
Functional protein distribution in groups 10 mgF/L vs. control group. Functional distribution of proteins identified with differences in expression in submandibular gland of mice chronically exposed (60 days) to drinking water containing 10 mgF/L vs. control group (deionized water). Protein categories were based on Gene Ontology (GO) for biological processes. The access number of the proteins was provided by the UniProt database. The GO was evaluated according to the Cytoscape ® 3.7.1 software, using the ClueGo ^®^ plugin, adopting the significant terms (kappa score = 0.4) and distribution according to the percentage of the number of genes.

The bioinformatic analysis of biological processes, based on gene ontologies, showed that the group exposed to 10 mgF/L had 18 biological processes modulated (Figure 4), in which the five most changed were intermediate filament cytoskeleton organization (16%), ion transmembrane transporter activity, phosphorylative mechanism (15%), actin-myosin filament sliding (7%), regulation of blood volume by renin-angiotensin (6%), and flavonoid glucuronidation (6%).

**FIGURE 5:**
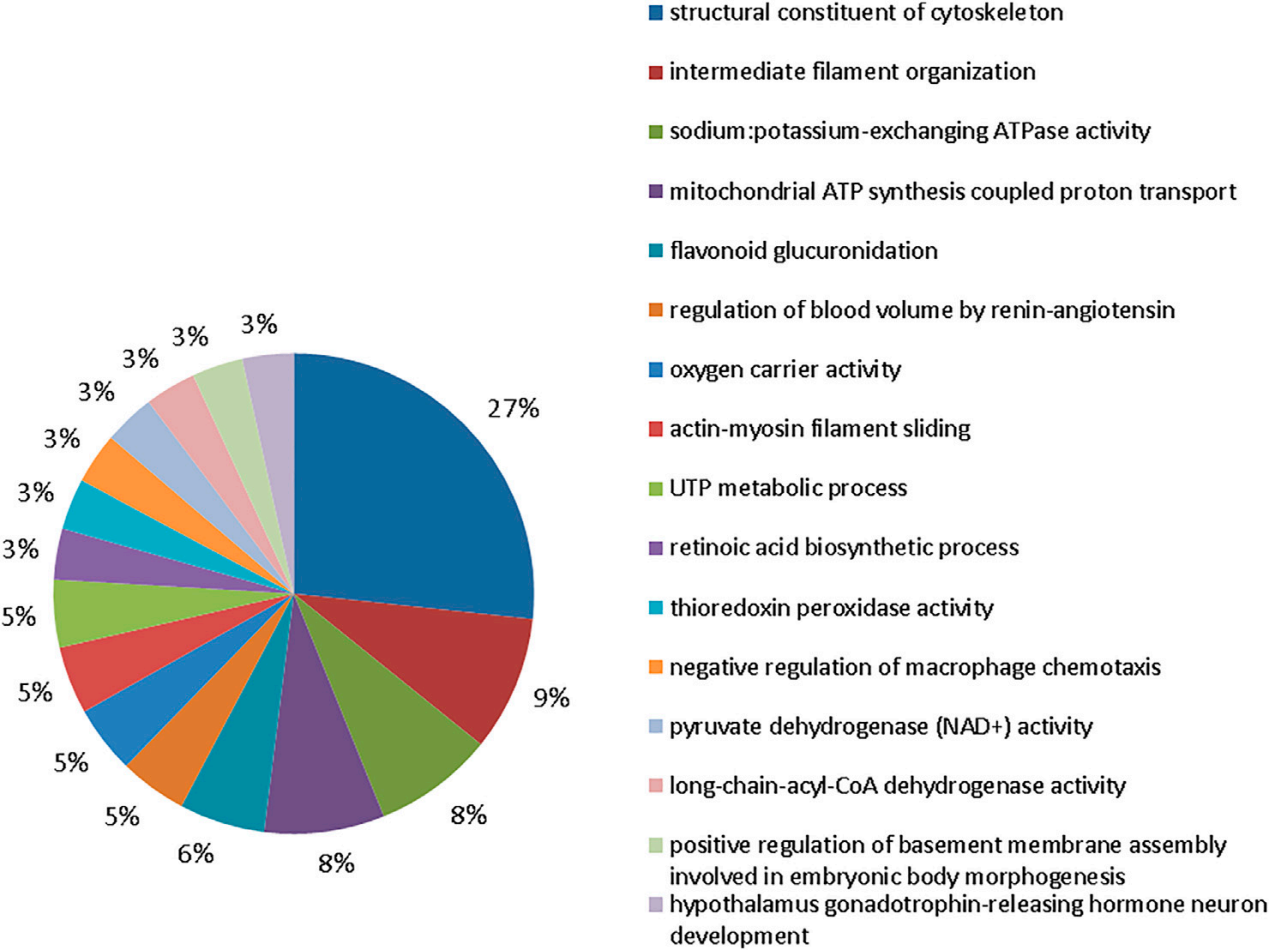
Functional distribution of proteins in the 50 mgF/L vs. control groups. Functional distribution of proteins identified with expression difference in submandibular gland of mice chronically exposed (60 days) to drinking water containing 50 mgF/L vs. control group (deionized water). Protein categories based on Gene Ontology (GO) selected for biological processes. The accession number of the proteins was provided by the UniProt data bank. The GO was evaluated according to the Cytoscape® 3.7.1 software, using the ClueGo® plugin, adopting the significant terms (kappa score = 0.4) and distribution according to the percentage of the number of genes.

The group exposed to 50 mgF/L also showed 16 functional categories affected (Figure 5). The five most affected were: structural constituent of cytoskeleton (27%), intermediate filament organization (9%), sodium:potassium–exchanging ATPase activity (8%), mitochondrial ATP synthesis–coupled proton transport (8%), and flavonoid glucuronidation (6%).

**FIGURE 6:**
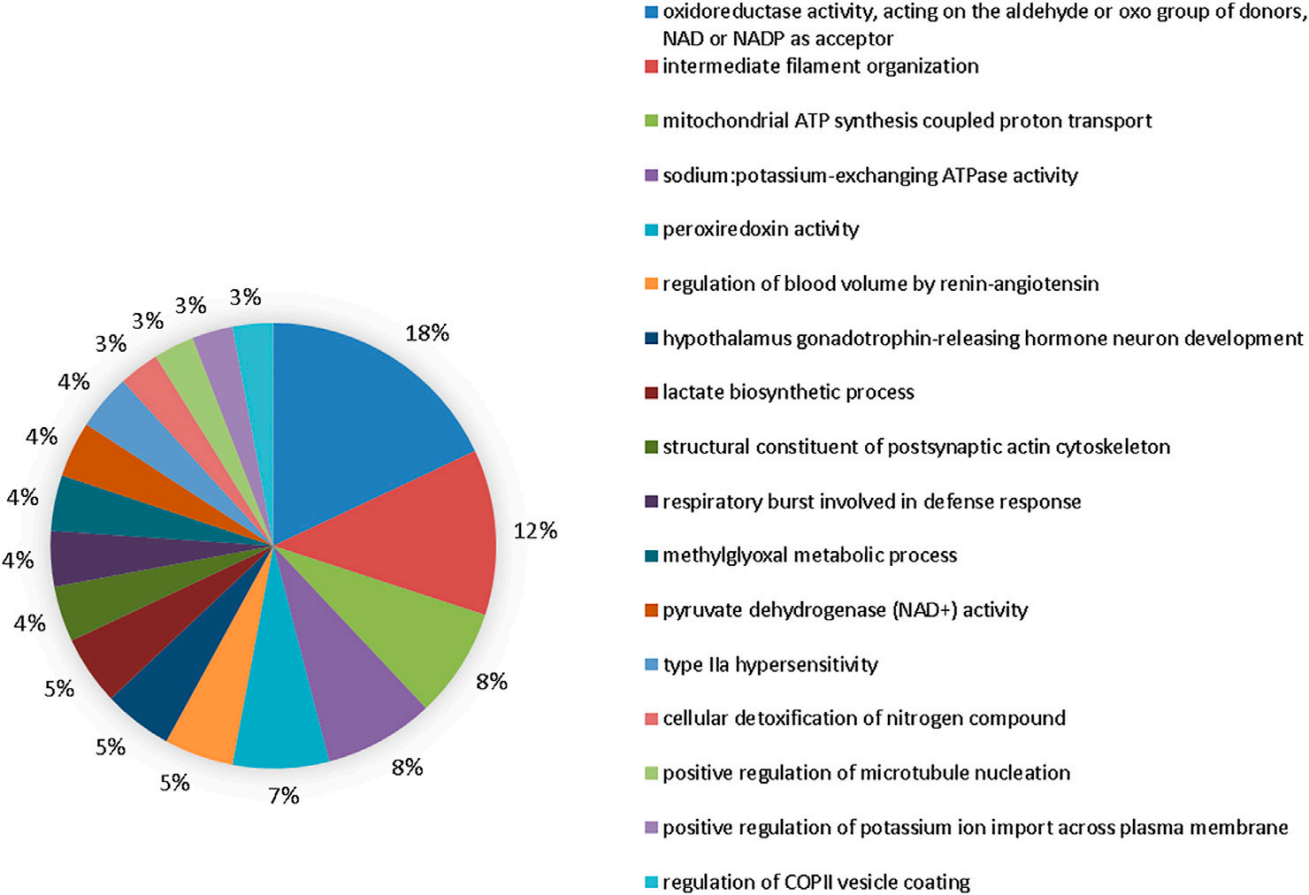
Functional distribution of proteins in the 50 mgF/L vs. 10 mgF/L. Functional distribution of proteins identified with expression difference in the submandibular gland of mice chronically exposed (60 days) to drinking water containing 50 mgF/L vs. 10 mgF/L. Protein categories based on Gene Ontology (GO) selected for biological processes. The accession number of the proteins was provided by the UniProt data bank. The GO was evaluated according to the Cytoscape ® 3.7.1 software, using the ClueGo ® plugin, adopting the significant terms (kappa score = 0.4) and distribution according to the percentage of the number of genes.

The comparison group at 50 mgF/L vs. 10 mgF/L showed 20 functional categories affected (Figure 6). The five most affected were oxidoreductase activity, acting on the aldehyde or oxo group of donors, NAD or NADP as acceptor (17%), intermediate filament organization (11%), mitochondrial ATP synthesis–coupled proton transport (7%), sodium:potassium–exchanging ATPase activity (7%), and peroxiredoxin activity (6%).

**FIGURE 7:**
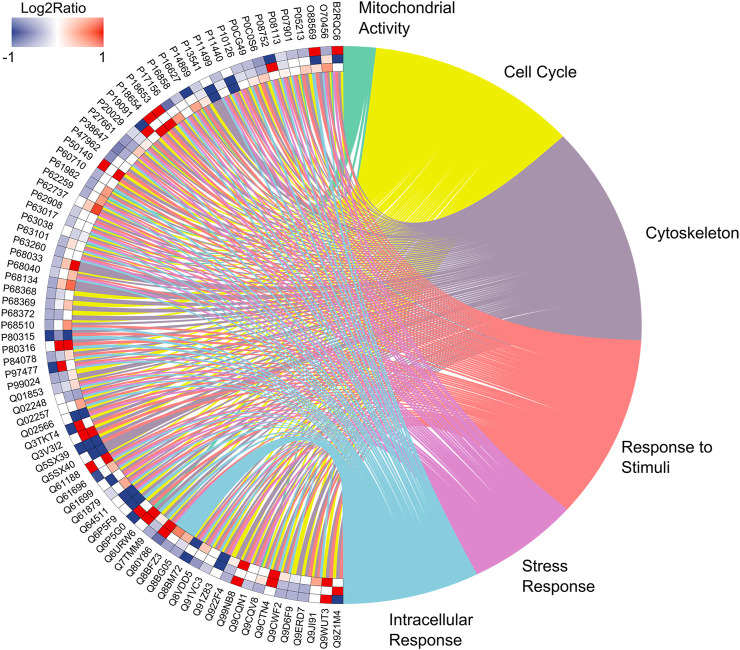
Enrichment analysis. Over-represented proteins in the submandibular gland of exposed mice for 60 days at two different concentrations of F, following the edge to center, for the comparisons: 50 mgF/L vs. 10 mgF/L, 50 mgF/L vs. control, and 10 mgF/L vs. control. Protein categories based on Gene Ontology (GO) selected for biological processes. The color indicates the differential expression of each protein, which is represented with its access code. Red indicates upregulation and blue, downregulation. The tone varies according to the intensity of the adjustment.

Based on the analysis of the proteomic profile, we compiled the data to evaluate protein–protein interactions with the circosplot tool. It is possible to observe 81 proteins with a greater number of interactions with others in each group. Moreover, these proteins are associated with 6 major biological processes as mitochondrial activity, cell cycle, cytoskeleton, response to stimuli, and stress response and intracellular response (Supplementary Table S4; Figure 7). The quantitative description is available in Table 2.

**TABLE 2:** Number of negatively regulated and positively regulated proteins in the submandibular glands, for the comparisons: 10 mgF/L vs. control, 50 mgF/L vs. control, and 50 mgF/L vs. 10 mgF/L, according to the ORA analysis in the protein criterion with the largest protein interaction network.

Category	10 mgF/L *vs.* control			50 mgF/L *vs.* control			50 mgF/L *vs.* 10 mgF/L		
	Down	Up	Total	Down	Up	Total	Down	Up	Total
Mitochondria activity	-	5	5	2	4	6	10	1	11
Cell cycle	9	34	43	29	13	42	51	7	58
Cytoskeleton	12	39	51	33	17	50	54	8	62
Response to stimuli	8	33	41	24	14	38	45	8	53
Stress response	7	16	23	15	6	21	26	4	30
Intracellular response	7	25	32	17	8	25	34	22	36

DOWN, downregulated; UP, upregulated.

### The Exposure to the Highest F Concentration Led to Genotoxic Effects on Submandibular Gland of Mice

**FIGURE 8:**
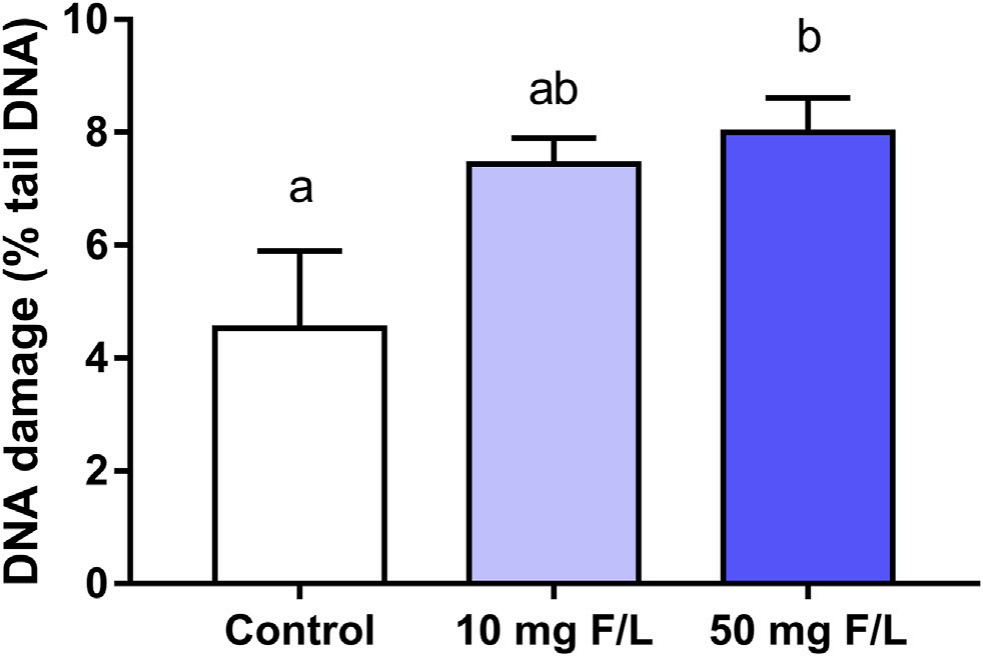
Analysis of the genotoxicity. The Comet assay in submandibular gland of mice chronically exposed (60 days) drinking water containing 0, 10, and 50 mgF/L, during 60 days. The rate of genotoxic damage was represented as a percentage of DNA in the tail. One-way ANOVA and Tukey’s post hoc test, *p* < 0.05. Different lowercase letters indicate significant differences among the groups.

The genotoxic analysis showed a significant difference in the DNA damage (% DNA in the tail) between the 50 mgF/L (7.997 ± 1.505%) and the control group (4.530 ± 2.737%; *p* = 0.0233). The 10 mgF/L group did not differ from the control and 50 mgF/L groups (*p* > 0.05) (Figure 8).

## Discussion

To the best of our knowledge, this is the first study that simultaneously investigated the biochemical, proteomic, and genotoxic parameters of rat’s submandibular glands after long-term exposure to two relevant F concentrations. These concentrations mimic plasma F levels observed in humans that regularly consume fluoridated water or live in areas of endemic fluorosis (Angmar-Månsson and Whitford, 1984; Dunipace et al., 1995; Dionizio et al., 2018) and have been often used in other studies (Carvalho et al., 2009; Melo et al., 2017). It is worth mentioning that the World Health Organization (WHO) established 0.5 to 1.0 mgF/L as the ideal concentration range for drinking water (World Health, 2017). This study indicated that submandibular glands are important targets for F since biochemical modulations that lead to oxidative imbalance (with increased lipid peroxidation), changes in the proteomic profile of the glandular tissue, and genotoxic effects were observed in a dose-dependent trend. Moreover, several potential biomarkers were identified in the proteomic analysis as well as other impairments triggered by F.

Incorporation into mineralized tissues and, to a lesser extent, accumulation in soft tissues are the major consequences of excessive F intake since this highly reactive electronegative element associates with positively charged ions in particular aluminum, calcium, and iron (Sauerheber, 2013; Miranda et al., 2018). Therefore, F can interfere in the expression of proteins related to cellular activity as observed in the proteomic analysis (Figures 4–6). Alterations in the proteins responsible for the maintenance of the electrochemical gradient across the plasma membrane (at the expense of ATP) and transportation of other important ions and metabolites related to ionic homeostasis and cell signaling were altered.

In this sense, the inactivity of proteins that regulate sodium and potassium gradients is associated with several pathophysiological disorders, such as asthma, allergies, metabolic disorders, cancer, cardiovascular diseases, neurodevelopmental, neuropsychiatric, and neurodegenerative diseases (Waugh, 2019). Several studies have shown that depending on the dose, cell type, and tissue, F can inhibit enzymes such as enolase, pyruvate kinase, lactate dehydrogenase, and potassium-activated adenosine triphosphatase (Strunecka et al., 2007) and can stimulate adenylate cyclase, alanine transaminase, lactate dehydrogenase, and glycogen phosphorylase. Therefore, F can alter energy metabolism, induce oxidative stress, inflammation, and immunoexcitotoxicity, albeit the respective mechanisms are still unclear (Araujo et al., 2019; Strunecka and Strunecky, 2019).

Oxidative biochemistry is closely related to the cellular proteostasis network since stress conditions influence the behavior of proteins (Reichmann et al., 2018). Despite proteomic changes in tissues such as the kidneys, liver, and intestine being widely reported in the literature (Xu et al., 2005; Kobayashi et al., 2009; Dionizio et al., 2018; Pereira et al., 2018; Khan et al., 2019; Dionizio et al., 2020), the results of this study demonstrated that F altered the expression of several glandular proteins.

The oxidative stress observed in submandibular glands suggests that F may trigger cell damage mechanisms as a result of an imbalanced release of antioxidant and prooxidant compounds. These results were confirmed by the biochemical analyses, which showed an increase in lipid peroxidation through the TBARS method and genotoxicity (Figure 3). Exposure to F increases the generation of superoxide (O_2_^-^) and other reactive oxygen species (ROS), which leads to oxidative imbalance, mitochondrial dysfunction, and DNA damage. This sequence of events can cause cell death, as previously demonstrated in studies, with peripheral blood and cerebellum (Miranda et al., 2018; Lopes et al., 2020).

The imbalance between ROS and the antioxidant system characterizes the so-called oxidative impairment, which is an important factor in the toxicity mechanism that affects cell integrity (Halliwell, 2007). One of the toxicity mechanisms is related to the interaction between F and enzymes that can inhibit antioxidant activities (SOD, GSH, and CAT levels) (Ma et al., 2017). Organisms usually have several mechanisms for protection against ROS such as the endogenous antioxidant molecules GSH and SOD (Halliwell, 2007). Moreover, the results from the enzymatic assays in this study demonstrated that chronic exposure to F increased GSH levels, which suggests that F triggered protective mechanisms to protect the integrity of cellular biomolecules (Figure 3). The increase in antioxidant enzymes has been observed in rat liver mitochondria after exposure to 15 mgF/L for 60 days (Araujo et al., 2019).

The TBARS measurement is a very relevant technique to assess oxidative damage in lipids and quantify the by-products of tissue lipid peroxidation (Liu et al., 1997). The increase in the TBARS increases the peroxidation of polyunsaturated fatty acids of cell membranes, which in turn indicates the presence of oxidative stress (Gutiérrez-Salinas et al., 2013). The animals chronically exposed to F in this study showed higher TBARS levels than the control, which indicates lipid peroxidation and potential damage to the cell membrane; thus, the F dose–response trend is suggested.

The bioinformatics analysis showed positive regulation of stress response proteins (so-called heat shock proteins—HSP) such as HSP 70 kDa 1-like (P16627), HSP 70 kDa 1A (Q61696), and HSP-related 70 kDa 2 (P17156) after exposure to 10 mgF/L (Supplementary Table S4). These chaperones proteins play a key role in the synthesis, assembly, folding, protein degradation, and cell survival under adverse environmental conditions (Gupta et al., 2007). Stressful situations such as oxidative or osmotic stress increase HSP levels, which support the synthesis and maturation of proteins to replace those affected by the metabolic alteration (Bukau and Horwich, 1998). The results of this study suggest that positive regulation of HSP may occur due to a response to oxidative biochemistry modulation.

The proteomic profile revealed changes in the modulation of protein functional groups mainly related to the cellular cytoskeleton such as “structural constituent of cytoskeleton” and “intermediate filament cytoskeleton organization” as well as to muscle contraction such as “actin-myosin filament sliding.” Figures 4–6 objectively indicate the main protein changes that can compromise submandibular glands, while Figure 7 categorizes them according to function in the over-representation analysis (ORA). This technique is widely used to assess the role of gene sets, transcripts, proteins, or metabolites; in addition, it also determines whether known biological functions or processes are over-represented among a group of identified analytes (Tavazoie et al., 1999; Goeman and Bühlmann, 2007; Khatri et al., 2012; Jeuken and Käll, 2018).

The cellular cytoskeleton is mainly composed of microfilaments, intermediate filaments, and microtubules. Alpha- and beta-tubulin dimers form the microtubules related to specific proteins and regulate certain signaling pathways mediated by the G protein. Previous studies indicate that F alters the structure, metabolism, and dynamics of the microtubular system (Wang et al., 1990;Kovács et al., 2008;Monteiro et al., 2011). In this study, the beta-tubulin protein-3 (Q9ERD7), beta-4 (Q9D6F9), beta-4B (P68372), beta-2B (Q9CWF2), beta-5 (P99024) beta-2A (Q7TMM9), beta-6 (Q922F4), alpha-1a (P68369), and alpha-4a (P68368) were downregulated after exposure to the 50 mgF/L group (Supplementary Table S4).

Myosin converts chemical energy into mechanical work through cyclic interaction with actin filaments and generates strength and movement during muscle contraction. These proteins are found between the basal laminas of acini and ducts of myoepithelial cells, which are located in the terminal portion of salivary glands (Redman, 1994). These cells help to expel glandular secretory products into the excretory duct system. Studies have shown that F degenerates the structure and functions of the actin and myosin filaments in the skeletal muscle (Shashi, 1989, 1992; Park et al., 1999). Negative expressions of the proteins myosin-7 (Q91Z83), myosin-1 (Q5SX40), myosin-3 (P13541), myosin-4 (Q5SX39), and myosin-6 (Q02566) after exposure to both 10 mgF/L and 50 mgF/L were observed in this study, which indicates that F affects a wide range of cellular events related to motility and potential adverse consequences for submandibular gland contraction may occur.

The chromatin structure is the main modulator of all DNA-based processes such as genetic transcription, replication, and DNA repair. The basic unit of chromatin (nucleosome) is composed of DNA and histone proteins. Both Histone H2A.Z (P0C0S6) and Histone H2AX (P27661) play important roles in the regulation of chromatin structure, genetic transcription, DNA replication and repair, and maintenance of genome integrity (Talbert and Henikoff, 2017; Wang et al., 2018; Long et al., 2020).

The transitional endoplasmic reticulum ATPase protein (Q01853) is associated with nuclear envelope reconstruction, transcriptional control, cell cycle regulation, and DNA damage response (Rabinovich et al., 2002; Lilley and Ploegh, 2005; Meyer, 2012; Erzurumlu et al., 2013). In this study, these proteins were positively regulated after exposure to 10 mgF/L, which suggests an attempt to cell repair; however, these proteins were negatively regulated when exposed to 50 mgF/L, which indicates a potential loss of function (Supplementary Table S4).

Oxidative changes can modulate protein expression and damage nucleic acids, which induce DNA strand fragmentation (Mendes Arent et al., 2014). It is known that F-induced genetic damage can be triggered by oxidative biochemical mechanisms (Ribeiro et al., 2017); therefore, this study aimed to investigate potential damages to the nuclear components based on the biochemical and proteomic changes triggered by fluoride exposure. The results showed that DNA strand break only after exposure to 50 mgF/L, which was also observed in hepatocytes of rats exposed to 50 mgF/L for 120 days (Song et al., 2015) and both hepatocytes and oral cells of rats exposed to 150 mgNaF/L for 4 weeks (He and Chen, 2006). The percentage of DNA in the comet tail (12.37 ± 2.68) caused by the prolonged F exposure used by Song et al. (2015) is in accordance with the results of the present study; however, the increased F concentration combined with short exposure used by He and Chen (2006) induced about 50% of damage in both cell types.

These findings corroborate with the present proteomic analysis since negative regulation of proteins related to repair of DNA damage repair was observed after exposure to 50 mgF/L, which might have promoted DNA strand breaks. However, Buzalaf et al. (2006) did not observe significant DNA damage in the liver, kidneys, and urinary bladder of rats that drank 100 mgNaF/L-containing water for 75 days. These data suggest that submandibular glands are more susceptible to the F adverse effects than other organs.

The findings of this study corroborate with the literature regarding the F potential to promote cellular changes, which are very dependent on F concentration, exposure time, and the cell/organ type. Significant changes were observed in rats exposed to fluoridated drinking water at similar concentrations reported in areas of endemic skeletal fluorosis (50 mgF/L). Conversely, the lower F concentration induced only minor alterations.

## Conclusion

Our data revealed modulation of the oxidative and proteomic biochemistry of submandibular gland after exposure to F in mice at concentrations of 10 or 50 mgF/L, as well as genotoxic changes promoted by exposure to the highest concentration (50 mgF/L). These data reinforce the need of finding alternative sources of potable water for people living in endemic areas of fluorosis. Moreover, our findings reinforce the importance of permanent monitoring of F concentrations in the drinking water and of strategies to minimize F intake from other sources, to achieve maximum preventive/therapeutic effects and avoid potential side effects.

## Data Availability

The datasets presented in this study can be found in online repositories. The names of the repository/repositories and accession number(s) can be found below: Peptide Atlas, accession no: PASS01690.
