# Supplementary material for: Effects of Fluoride on Submandibular Glands of Mice: Changes in Oxidative Biochemistry, Proteomic Profile, and Genotoxicity
Source: Front Pharmacol. 2021 Sep 27;12:715394. doi: 10.3389/fphar.2021.715394 (PMC8503261; doi:10.3389/fphar.2021.715394)
Supplement: Supplementary file 1 [file DataSheet1.docx]

Supplementary Material

**Table S1**. Proteins with different expression significantly altered in the submandibular glands of mice exposed to 10 mgF/L vs. control

| ***^a^*Access Number** | **Protein name description** | **PLGS**  **Score** | **^b^Ratio**  **10 mgF/ L: control** |
| --- | --- | --- | --- |
| Q91VU6 | DDB1- and CUL4-associated factor 11 | 186 | 165.67 |
| Q70FJ1 | A-kinase anchor protein 9 | 95 | 11.82 |
| P08730 | Keratin, type I cytoskeletal 13 | 185 | 3.00 |
| P97861 | Keratin, type II cuticular Hb6 | 55 | 2.80 |
| Q9ERE2 | Keratin, type II cuticular Hb1 | 55 | 2.66 |
| Q6IMF0 | Keratin, type II cuticular 87 | 55 | 2.64 |
| Q9R0P5 | Destrin | 254 | 2.59 |
| Q9Z2T6 | Keratin, type II cuticular Hb5 | 55 | 2.59 |
| P03995 | Glial fibrillary acidic protein | 55 | 2.53 |
| Q6ZQF0 | DNA topoisomerase 2-binding protein 1 | 290 | 2.51 |
| P10107 | Annexin A1 | 173 | 2.44 |
| Q99L20 | Glutathione S-transferase theta-3 | 89 | 2.25 |
| P00756 | Kallikrein 1-related peptidase b3 | 13563 | 2.16 |
| P31001 | Desmin | 78 | 2.05 |
| Q6P9K9 | Neurexin-3 | 122 | 2.03 |
| Q9Z1W8 | Potassium-transporting ATPase alpha chain 2 | 116 | 2.03 |
| P68033 | Actin, alpha cardiac muscle 1 | 33613 | 2.01 |
| P06281 | Renin-1 | 7733 | 1.97 |
| Q6ZQA0 | Neurobeachin-like protein 2 | 84 | 1.92 |
| Q9WV27 | Sodium/potassium-transporting ATPase subunit alpha-4 | 143 | 1.92 |
| P54818 | Galactocerebrosidase | 82 | 1.90 |
| P62737 | Actin, aortic smooth muscle | 31651 | 1.82 |
| Q61414 | Keratin, type I cytoskeletal 15 | 218 | 1.79 |
| P05202 | Aspartate aminotransferase, mitochondrial | 288 | 1.77 |
| P15948 | Kallikrein 1-related peptidase b22 | 40034 | 1.77 |
| Q9JM71 | Kallikrein 1-related peptidase b27 | 18515 | 1.75 |
| P60867 | 40S ribosomal protein S20 | 260 | 1.72 |
| O08553 | Dihydropyrimidinase-related protein 2 | 148 | 1.68 |
| P19157 | Glutathione S-transferase P 1 | 120 | 1.68 |
| Q61759 | Kallikrein 1-related peptidase b21 | 21122 | 1.68 |
| Q8BGZ7 | Keratin, type II cytoskeletal 75 | 332 | 1.68 |
| E9Q8I9 | Protein furry homolog | 84 | 1.68 |
| Q6PIC6 | Sodium/potassium-transporting ATPase subunit alpha-3 | 189 | 1.68 |
| P62631 | Elongation factor 1-alpha 2 | 3859 | 1.67 |
| Q3UU35 | Ovostatin homolog | 113 | 1.67 |
| Q62261 | Spectrin beta chain, non-erythrocytic 1 | 72 | 1.67 |
| P68134 | Actin, alpha skeletal muscle | 33054 | 1.65 |
| P07628 | Kallikrein 1-related peptidase b8 | 17164 | 1.63 |
| Q3UV17 | Keratin, type II cytoskeletal 2 oral | 83 | 1.63 |
| P00796 | Renin-2 | 9834 | 1.63 |
| Q8VCK3 | Tubulin gamma-2 chain | 103 | 1.63 |
| Q02788 | Collagen alpha-2(VI) chain | 128 | 1.62 |
| Q8BTM8 | Filamin-A | 417 | 1.62 |
| P46425 | Glutathione S-transferase P 2 | 120 | 1.62 |
| Q99J77 | Sialic acid synthase | 732 | 1.62 |
| P01869 | Ig gamma-1 chain C region, membrane-bound form | 269 | 1.60 |
| P15949 | Kallikrein 1-related peptidase b9 | 40806 | 1.60 |
| Q9JJG0 | Transforming acidic coiled-coil-containing protein 2 | 120 | 1.60 |
| O70251 | Elongation factor 1-beta | 492 | 1.58 |
| Q8CI43 | Myosin light chain 6B | 485 | 1.58 |
| P47791 | Glutathione reductase, mitochondrial | 86 | 1.57 |
| P62806 | Histone H4 | 16551 | 1.57 |
| Q922U2 | Keratin, type II cytoskeletal 5 | 336 | 1.57 |
| Q3V132 | ADP/ATP translocase 4 | 119 | 1.55 |
| Q9D6P8 | Calmodulin-like protein 3 | 166 | 1.55 |
| P18242 | Cathepsin D | 776 | 1.55 |
| Q9QWL7 | Keratin, type I cytoskeletal 17 | 218 | 1.55 |
| Q99KK2 | N-acylneuraminate cytidylyltransferase | 116 | 1.55 |
| Q3UQ44 | Ras GTPase-activating-like protein IQGAP2 | 54 | 1.55 |
| Q60854 | Serpin B6 | 3395 | 1.55 |
| Q8BFZ3 | Beta-actin-like protein 2 | 9060 | 1.54 |
| Q04857 | Collagen alpha-1(VI) chain | 95 | 1.54 |
| P83887 | Tubulin gamma-1 chain | 103 | 1.52 |
| P68433 | Histone H3.1 | 1632 | 1.51 |
| P84228 | Histone H3.2 | 1632 | 1.51 |
| Q64518 | Sarcoplasmic/endoplasmic reticulum calcium ATPase 3 | 88 | 1.51 |
| Q8VDN2 | Sodium/potassium-transporting ATPase subunit alpha-1 | 333 | 1.51 |
| P84244 | Histone H3.3 | 1632 | 1.49 |
| P02301 | Histone H3.3C | 1632 | 1.49 |
| P36369 | Kallikrein 1-related peptidase b26 | 37887 | 1.48 |
| Q99M73 | Keratin, type II cuticular Hb4 | 114 | 1.48 |
| Q6PIE5 | Sodium/potassium-transporting ATPase subunit alpha-2 | 189 | 1.48 |
| P00405 | Cytochrome c oxidase subunit 2 | 200 | 1.46 |
| P05977 | Myosin light chain 1/3, skeletal muscle isoform | 988 | 1.46 |
| Q62186 | Translocon-associated protein subunit delta | 138 | 1.46 |
| P68510 | 14-3-3 protein eta | 999 | 1.45 |
| P36368 | Epidermal growth factor-binding protein type B | 21370 | 1.45 |
| Q61781 | Keratin, type I cytoskeletal 14 | 55 | 1.45 |
| Q6IFX2 | Keratin, type I cytoskeletal 42 | 55 | 1.45 |
| P50396 | Rab GDP dissociation inhibitor alpha | 167 | 1.45 |
| O70456 | 14-3-3 protein sigma | 999 | 1.43 |
| P04104 | Keratin, type II cytoskeletal 1 | 198 | 1.43 |
| Q9R0H5 | Keratin, type II cytoskeletal 71 | 121 | 1.43 |
| O09159 | Lysosomal alpha-mannosidase | 185 | 1.43 |
| P61982 | 14-3-3 protein gamma | 999 | 1.42 |
| P48036 | Annexin A5 | 612 | 1.42 |
| P17156 | Heat shock-related 70 kDa protein 2 | 1604 | 1.42 |
| P15946 | Kallikrein 1-related peptidase b11 | 14312 | 1.42 |
| Q8VED5 | Keratin, type II cytoskeletal 79 | 82 | 1.42 |
| P07309 | Transthyretin | 465 | 1.42 |
| A2AQ07 | Tubulin beta-1 chain | 79 | 1.42 |
| P62259 | 14-3-3 protein epsilon | 1445 | 1.40 |
| Q9D3D9 | ATP synthase subunit delta, mitochondrial | 244 | 1.40 |
| Q61754 | Kallikrein 1-related peptidase b24 | 11318 | 1.40 |
| P53657 | Pyruvate kinase PKLR | 130 | 1.40 |
| P48962 | ADP/ATP translocase 1 | 227 | 1.39 |
| P97315 | Cysteine and glycine-rich protein 1 | 456 | 1.39 |
| P11352 | Glutathione peroxidase 1 | 437 | 1.39 |
| P07744 | Keratin, type II cytoskeletal 4 | 82 | 1.39 |
| Q6IME9 | Keratin, type II cytoskeletal 72 | 452 | 1.39 |
| P25444 | 40S ribosomal protein S2 | 288 | 1.38 |
| P15626 | Glutathione S-transferase Mu 2 | 569 | 1.38 |
| P19001 | Keratin, type I cytoskeletal 19 | 1031 | 1.38 |
| Q3TTY5 | Keratin, type II cytoskeletal 2 epidermal | 399 | 1.38 |
| P50446 | Keratin, type II cytoskeletal 6A | 118 | 1.38 |
| Q60817 | Nascent polypeptide-associated complex subunit alpha | 129 | 1.38 |
| P07724 | Serum albumin | 16426 | 1.38 |
| Q9WVB2 | Transducin-like enhancer protein 2 | 107 | 1.38 |
| Q91WD4 | UPF0415 protein C7orf25 homolog | 113 | 1.38 |
| Q9JI91 | Alpha-actinin-2 | 138 | 1.36 |
| Q80X90 | Filamin-B | 97 | 1.36 |
| P16627 | Heat shock 70 kDa protein 1-like | 1613 | 1.36 |
| Q02257 | Junction plakoglobin | 110 | 1.36 |
| Q6IFZ6 | Keratin, type II cytoskeletal 1b | 386 | 1.36 |
| Q6NXH9 | Keratin, type II cytoskeletal 73 | 386 | 1.36 |
| P34884 | Macrophage migration inhibitory factor | 838 | 1.36 |
| P08553 | Neurofilament medium polypeptide | 62 | 1.36 |
| Q78PY7 | Staphylococcal nuclease domain-containing protein 1 | 322 | 1.36 |
| P51881 | ADP/ATP translocase 2 | 1658 | 1.35 |
| P01027 | Complement C3 | 59 | 1.35 |
| D3Z0U5 | Katanin p60 ATPase-containing subunit A-like 2 | 141 | 1.35 |
| Q3THE2 | Myosin regulatory light chain 12B | 2813 | 1.35 |
| Q9CQ19 | Myosin regulatory light polypeptide 9 | 2961 | 1.35 |
| P24549 | Retinal dehydrogenase 1 | 217 | 1.35 |
| Q68FD5 | Clathrin heavy chain 1 | 134 | 1.34 |
| Q61879 | Myosin-10 | 65 | 1.34 |
| P45376 | Aldose reductase | 174 | 1.32 |
| Q00897 | Alpha-1-antitrypsin 1-4 | 404 | 1.32 |
| Q8BW94 | Dynein heavy chain 3, axonemal | 99 | 1.32 |
| P58252 | Elongation factor 2 | 699 | 1.32 |
| P06467 | Hemoglobin subunit zeta | 309 | 1.32 |
| Q9DCV7 | Keratin, type II cytoskeletal 7 | 2797 | 1.32 |
| Q60605 | Myosin light polypeptide 6 | 1247 | 1.32 |
| P01132 | Pro-epidermal growth factor | 364 | 1.32 |
| Q99PT1 | Rho GDP-dissociation inhibitor 1 | 318 | 1.32 |
| P29621 | Serine protease inhibitor A3C | 130 | 1.32 |
| Q80X76 | Serine protease inhibitor A3F | 130 | 1.32 |
| P14824 | Annexin A6 | 403 | 1.31 |
| P05784 | Keratin, type I cytoskeletal 18 | 5478 | 1.31 |
| O08807 | Peroxiredoxin-4 | 268 | 1.31 |
| Q9D051 | Pyruvate dehydrogenase E1 component subunit beta, mitochondrial | 298 | 1.31 |
| P20108 | Thioredoxin-dependent peroxide reductase, mitochondrial | 513 | 1.31 |
| O35945 | Aldehyde dehydrogenase, cytosolic 1 | 201 | 1.30 |
| P12787 | Cytochrome c oxidase subunit 5A, mitochondrial | 1026 | 1.30 |
| P43024 | Cytochrome c oxidase subunit 6A1, mitochondrial | 656 | 1.30 |
| Q8BG05 | Heterogeneous nuclear ribonucleoprotein A3 | 203 | 1.30 |
| P00755 | Kallikrein 1-related peptidase b1 | 24880 | 1.30 |
| Q6IFZ9 | Keratin, type II cytoskeletal 74 | 142 | 1.30 |
| P11679 | Keratin, type II cytoskeletal 8 | 3580 | 1.30 |
| Q91WP6 | Serine protease inhibitor A3N | 130 | 1.30 |
| P62889 | 60S ribosomal protein L30 | 983 | 1.28 |
| Q64467 | Glyceraldehyde-3-phosphate dehydrogenase, testis-specific | 943 | 1.28 |
| Q61696 | Heat shock 70 kDa protein 1A | 1558 | 1.28 |
| P17879 | Heat shock 70 kDa protein 1B | 1561 | 1.28 |
| P08228 | Superoxide dismutase [Cu-Zn] | 1996 | 1.28 |
| Q3UX10 | Tubulin alpha chain-like 3 | 101 | 1.28 |
| O88990 | Alpha-actinin-3 | 105 | 1.27 |
| P00757 | Kallikrein 1-related peptidase-like b4 | 5700 | 1.27 |
| Q01768 | Nucleoside diphosphate kinase B | 4265 | 1.27 |
| P97351 | 40S ribosomal protein S3a | 247 | 1.26 |
| Q7TPR4 | Alpha-actinin-1 | 273 | 1.26 |
| P56480 | ATP synthase subunit beta, mitochondrial | 5560 | 1.26 |
| P28654 | Decorin | 98 | 1.26 |
| P04071 | Kallikrein 1-related peptidase b16 | 16582 | 1.26 |
| P50580 | Proliferation-associated protein 2G4 | 190 | 1.26 |
| P68040 | Receptor of activated protein C kinase 1 | 340 | 1.26 |
| P01837 | Ig kappa chain C region | 858 | 1.25 |
| Q07417 | Short-chain specific acyl-CoA dehydrogenase, mitochondrial | 293 | 1.25 |
| P14094 | Sodium/potassium-transporting ATPase subunit beta-1 | 595 | 1.25 |
| P58774 | Tropomyosin beta chain | 1662 | 1.25 |
| Q6P8J7 | Creatine kinase S-type, mitochondrial | 106 | 1.23 |
| Q9CR68 | Cytochrome b-c1 complex subunit Rieske, mitochondrial | 512 | 1.23 |
| P60843 | Eukaryotic initiation factor 4A-I | 861 | 1.23 |
| A0A075B5P2 | Immunoglobulin kappa constant | 858 | 1.23 |
| Q8VDD5 | Myosin-9 | 64 | 1.23 |
| P68369 | Tubulin alpha-1A chain | 2012 | 1.23 |
| P63242 | Eukaryotic translation initiation factor 5A-1 | 310 | 1.22 |
| Q8R1M2 | Histone H2A.J | 30354 | 1.22 |
| Q3THW5 | Histone H2A.V | 5103 | 1.22 |
| P01867 | Ig gamma-2B chain C region | 398 | 1.22 |
| P09411 | Phosphoglycerate kinase 1 | 913 | 1.22 |
| P16546 | Spectrin alpha chain, non-erythrocytic 1 | 57 | 1.22 |
| P05214 | Tubulin alpha-3 chain | 1185 | 1.22 |
| Q9CXW4 | 60S ribosomal protein L11 | 568 | 1.21 |
| Q9D8N0 | Elongation factor 1-gamma | 349 | 1.21 |
| Q8CGP5 | Histone H2A type 1-F | 30354 | 1.21 |
| Q8CGP7 | Histone H2A type 1-K | 30354 | 1.21 |
| Q6GSS7 | Histone H2A type 2-A | 30354 | 1.21 |
| P27661 | Histone H2AX | 4710 | 1.21 |
| P68373 | Tubulin alpha-1C chain | 2012 | 1.21 |
| Q8CGP6 | Histone H2A type 1-H | 30354 | 1.20 |
| Q64523 | Histone H2A type 2-C | 30354 | 1.20 |
| Q8BFU2 | Histone H2A type 3 | 30354 | 1.20 |
| P0C0S6 | Histone H2A.Z | 5103 | 1.20 |
| P05213 | Tubulin alpha-1B chain | 2227 | 1.20 |
| P07758 | Alpha-1-antitrypsin 1-1 | 2093 | 1.19 |
| Q61598 | Rab GDP dissociation inhibitor beta | 245 | 1.19 |
| Q02053 | Ubiquitin-like modifier-activating enzyme 1 | 174 | 1.19 |
| P61205 | ADP-ribosylation factor 3 | 3776 | 1.17 |
| P22599 | Alpha-1-antitrypsin 1-2 | 2093 | 1.17 |
| Q00896 | Alpha-1-antitrypsin 1-3 | 2093 | 1.17 |
| Q9D154 | Leukocyte elastase inhibitor A | 134 | 1.17 |
| P54071 | Isocitrate dehydrogenase [NADP], mitochondrial | 211 | 1.16 |
| P14152 | Malate dehydrogenase, cytoplasmic | 2919 | 1.16 |
| P17751 | Triosephosphate isomerase | 6289 | 1.16 |
| Q9JJZ2 | Tubulin alpha-8 chain | 914 | 1.16 |
| P84078 | ADP-ribosylation factor 1 | 3776 | 1.15 |
| Q03265 | ATP synthase subunit alpha, mitochondrial | 6988 | 1.15 |
| P02104 | Hemoglobin subunit epsilon-Y2 | 8436 | 1.15 |
| P99029 | Peroxiredoxin-5, mitochondrial | 2221 | 1.14 |
| Q01853 | Transitional endoplasmic reticulum ATPase | 578 | 1.14 |
| Q9CZU6 | Citrate synthase, mitochondrial | 399 | 1.13 |
| Q9JIF7 | Coatomer subunit beta | 69 | 1.13 |
| P56391 | Cytochrome c oxidase subunit 6B1 | 3587 | 1.13 |
| P01942 | Hemoglobin subunit alpha | 22924 | 1.13 |
| O88569 | Heterogeneous nuclear ribonucleoproteins A2/B1 | 885 | 1.13 |
| Q9EQ20 | Methylmalonate-semialdehyde dehydrogenase [acylating], mitochondrial | 221 | 1.13 |
| Q921I1 | Serotransferrin | 2511 | 1.13 |
| P09671 | Superoxide dismutase [Mn], mitochondrial | 700 | 1.13 |
| P08003 | Protein disulfide-isomerase A4 | 95 | 1.12 |
| Q03734 | Serine protease inhibitor A3M | 78 | 1.12 |
| P14869 | 60S acidic ribosomal protein P0 | 1615 | 1.11 |
| P35979 | 60S ribosomal protein L12 | 588 | 1.11 |
| Q64524 | Histone H2B type 2-E | 5897 | 1.11 |
| Q9D2U9 | Histone H2B type 3-A | 5897 | 1.11 |
| P56565 | Protein S100-A1 | 5476 | 1.11 |
| Q9CWF2 | Tubulin beta-2B chain | 9950 | 1.11 |
| P99027 | 60S acidic ribosomal protein P2 | 4026 | 1.09 |
| Q64475 | Histone H2B type 1-B | 7628 | 1.09 |
| Q6ZWY9 | Histone H2B type 1-C/E/G | 7628 | 1.09 |
| Q64478 | Histone H2B type 1-H | 7628 | 1.09 |
| Q8CGP2 | Histone H2B type 1-P | 7628 | 1.09 |
| Q8CGP0 | Histone H2B type 3-B | 5897 | 1.09 |
| P81117 | Nucleobindin-2 | 837 | 1.09 |
| P40142 | Transketolase | 711 | 1.09 |
| Q7TMM9 | Tubulin beta-2A chain | 9950 | 1.09 |
| P99024 | Tubulin beta-5 chain | 10154 | 1.09 |
| P05064 | Fructose-bisphosphate aldolase A | 4066 | 1.08 |
| P08249 | Malate dehydrogenase, mitochondrial | 5216 | 1.08 |
| P0CG49 | Polyubiquitin-B | 10219 | 1.08 |
| P62983 | Ubiquitin-40S ribosomal protein S27a | 10219 | 1.08 |
| P10853 | Histone H2B type 1-F/J/L | 7628 | 1.07 |
| Q61171 | Peroxiredoxin-2 | 1227 | 1.07 |
| P0CG50 | Polyubiquitin-C | 10219 | 1.07 |
| Q922F4 | Tubulin beta-6 chain | 8367 | 1.07 |
| P20029 | 78 kDa glucose-regulated protein | 3678 | 1.06 |
| P07759 | Serine protease inhibitor A3K | 248 | 1.06 |
| P14211 | Calreticulin | 1792 | 1.05 |
| P08113 | Endoplasmin | 1411 | 1.05 |
| P15945 | Kallikrein 1-related peptidase b5 | 31682 | 0.96 |
| P63038 | 60 kDa heat shock protein, mitochondrial | 214 | 0.93 |
| Q9D6F9 | Tubulin beta-4A chain | 1444 | 0.93 |
| Q8CGP1 | Histone H2B type 1-K | 7628 | 0.90 |
| P10854 | Histone H2B type 1-M | 7628 | 0.90 |
| Q64525 | Histone H2B type 2-B | 7628 | 0.90 |
| Q99LC5 | Electron transfer flavoprotein subunit alpha, mitochondrial | 1260 | 0.87 |
| Q9DCW4 | Electron transfer flavoprotein subunit beta | 2096 | 0.87 |
| Q03402 | Cysteine-rich secretory protein 3 | 766 | 0.86 |
| P07743 | BPI fold-containing family A member 2 | 986 | 0.74 |
| Q02248 | Catenin beta-1 | 745 | 0.73 |
| Q91XA9 | Acidic mammalian chitinase | 795 | 0.71 |
| P00688 | Pancreatic alpha-amylase | 1706 | 0.63 |
| Q3UPL0 | Protein transport protein Sec31A | 153 | 0.59 |
| P00687 | Alpha-amylase 1 | 4746 | 0.58 |
| Q6ZWX6 | Eukaryotic translation initiation factor 2 subunit 1 | 101 | 0.52 |
| O70423 | Membrane primary amine oxidase | 152 | 0.52 |
| Q61900 | Submaxillary gland androgen-regulated protein 3A | 1611 | 0.47 |
| Q9Z1P8 | Angiopoietin-related protein 4 | 117 | 0.45 |
| Q501J2 | Protein FAM173A | 181 | 0.44 |
| Q8K3W0 | BRISC and BRCA1-A complex member 2 | 96 | 0.40 |
| Q8BIK4 | Dedicator of cytokinesis protein 9 | 154 | 0.40 |
| O55126 | Protein NipSnap homolog 2 | 160 | 0.38 |
| Q5NC05 | Transcription termination factor 2 | 142 | 0.37 |
| Q64727 | Vinculin | 103 | 0.36 |
| Q8BWT1 | 3-ketoacyl-CoA thiolase, mitochondrial | 204 | 0.35 |
| Q5SX40 | Myosin-1 | 334 | 0.31 |
| Q8CAF4 | NHS-like protein 1 | 102 | 0.29 |
| Q8R4U7 | Leucine zipper protein 1 | 181 | 0.26 |
| Q00558 | Factor VIII intron 22 protein | 128 | 0.25 |
| Q99JR1 | Sideroflexin-1 | 127 | 0.14 |
| Q9CX56 | 26S proteasome non-ATPase regulatory subunit 8 | 136 | 10 mg F / L* |
| Q91ZV4 | 2-acylglycerol O-acyltransferase 1 | 107 | 10 mg F / L |
| Q99L13 | 3-hydroxyisobutyrate dehydrogenase, mitochondrial | 147 | 10 mg F / L |
| Q9CZX8 | 40S ribosomal protein S19 | 690 | 10 mg F / L |
| Q9CQR2 | 40S ribosomal protein S21 | 929 | 10 mg F / L |
| Q9D8E6 | 60S ribosomal protein L4 | 103 | 10 mg F / L |
| Q9CQ60 | 6-phosphogluconolactonase | 300 | 10 mg F / L |
| P04756 | Acetylcholine receptor subunit alpha | 109 | 10 mg F / L |
| Q9Z2N8 | Actin-like protein 6A | 333 | 10 mg F / L |
| Q9QXN3 | Activating signal cointegrator 1 | 242 | 10 mg F / L |
| Q8K348 | Activin receptor type-1C | 234 | 10 mg F / L |
| Q9QYR9 | Acyl-coenzyme A thioesterase 2, mitochondrial | 134 | 10 mg F / L |
| P61211 | ADP-ribosylation factor-like protein 1 | 314 | 10 mg F / L |
| A2ASQ1 | Agrin | 74 | 10 mg F / L |
| Q9DBR4 | Amyloid-beta A4 precursor protein-binding family B member 2 | 163 | 10 mg F / L |
| P12023 | Amyloid-beta A4 protein | 64 | 10 mg F / L |
| Q9CZK6 | Ankyrin repeat and SAM domain-containing protein 3 | 57 | 10 mg F / L |
| Q6PD24 | Ankyrin repeat domain-containing protein 13D | 183 | 10 mg F / L |
| O88312 | Anterior gradient protein 2 homolog | 218 | 10 mg F / L |
| Q80WC7 | Arf-GAP domain and FG repeat-containing protein 2 | 120 | 10 mg F / L |
| Q4LDD4 | Arf-GAP with Rho-GAP domain, ANK repeat and PH domain-containing protein 1 | 482 | 10 mg F / L |
| Q61176 | Arginase-1 | 117 | 10 mg F / L |
| Q91YI0 | Argininosuccinate lyase | 184 | 10 mg F / L |
| Q3URY6 | Armadillo repeat-containing protein 2 | 284 | 10 mg F / L |
| Q3UD01 | Ataxin-7-like protein 3B | 186 | 10 mg F / L |
| Q5SSE9 | ATP-binding cassette sub-family A member 13 | 86 | 10 mg F / L |
| O88566 | Axin-2 | 115 | 10 mg F / L |
| P20060 | Beta-hexosaminidase subunit beta | 141 | 10 mg F / L |
| Q8R015 | Biogenesis of lysosome-related organelles complex 1 subunit 5 | 123 | 10 mg F / L |
| O35855 | Branched-chain-amino-acid aminotransferase, mitochondrial | 279 | 10 mg F / L |
| Q922D8 | C-1-tetrahydrofolate synthase, cytoplasmic | 68 | 10 mg F / L |
| O88338 | Cadherin-16 | 109 | 10 mg F / L |
| Q8BH59 | Calcium-binding mitochondrial carrier protein Aralar1 | 322 | 10 mg F / L |
| Q8K1N1 | Calcium-independent phospholipase A2-gamma | 74 | 10 mg F / L |
| Q9D805 | Calpain-9 | 137 | 10 mg F / L |
| P23953 | Carboxylesterase 1C | 104 | 10 mg F / L |
| Q8VCT4 | Carboxylesterase 1D | 239 | 10 mg F / L |
| Q8BK63 | Casein kinase I isoform alpha | 106 | 10 mg F / L |
| E9Q355 | Cation channel sperm-associated protein subunit gamma 1 | 68 | 10 mg F / L |
| C6KI89 | Cation channel sperm-associated protein subunit gamma 2 | 76 | 10 mg F / L |
| P27548 | CD40 ligand | 73 | 10 mg F / L |
| P51949 | CDK-activating kinase assembly factor MAT1 | 143 | 10 mg F / L |
| Q8CII2 | Cell division cycle protein 123 homolog | 75 | 10 mg F / L |
| Q9CXS4 | Centromere protein V | 165 | 10 mg F / L |
| Q6IRU7 | Centrosomal protein of 78 kDa | 62 | 10 mg F / L |
| B2RX88 | Centrosome and spindle pole associated protein 1 | 464 | 10 mg F / L |
| Q61410 | cGMP-dependent protein kinase 2 | 240 | 10 mg F / L |
| P0CG14 | Chromosome transmission fidelity protein 8 homolog isoform 2 | 247 | 10 mg F / L |
| Q9ESN9 | C-Jun-amino-terminal kinase-interacting protein 3 | 117 | 10 mg F / L |
| Q5XJY5 | Coatomer subunit delta | 60 | 10 mg F / L |
| Q3ULW6 | Coiled-coil domain-containing protein 33 | 96 | 10 mg F / L |
| Q4QRL3 | Coiled-coil domain-containing protein 88B | 52 | 10 mg F / L |
| P11087 | Collagen alpha-1(I) chain | 59 | 10 mg F / L |
| Q8BLX7 | Collagen alpha-1(XVI) chain | 231 | 10 mg F / L |
| Q01149 | Collagen alpha-2(I) chain | 56 | 10 mg F / L |
| Q8K2Z4 | Condensin complex subunit 1 | 88 | 10 mg F / L |
| Q8C0L8 | Conserved oligomeric Golgi complex subunit 5 | 112 | 10 mg F / L |
| Q9CPW0 | Contactin-associated protein-like 2 | 116 | 10 mg F / L |
| O88543 | COP9 signalosome complex subunit 3 | 123 | 10 mg F / L |
| Q8K2X3 | CST complex subunit STN1 | 397 | 10 mg F / L |
| Q3TCH7 | Cullin-4A | 282 | 10 mg F / L |
| A2A432 | Cullin-4B | 88 | 10 mg F / L |
| Q8BGU5 | Cyclin-Y | 82 | 10 mg F / L |
| Q9JHU4 | Cytoplasmic dynein 1 heavy chain 1 | 105 | 10 mg F / L |
| Q45VK7 | Cytoplasmic dynein 2 heavy chain 1 | 37 | 10 mg F / L |
| Q7TS74 | Cytoskeleton-associated protein 2-like | 119 | 10 mg F / L |
| Q99LN9 | Deoxyhypusine hydroxylase | 199 | 10 mg F / L |
| P97427 | Dihydropyrimidinase-related protein 1 | 127 | 10 mg F / L |
| B1AZP2 | Disks large-associated protein 4 | 202 | 10 mg F / L |
| Q9D7K5 | Distal membrane-arm assembly complex protein 2 | 131 | 10 mg F / L |
| P33611 | DNA polymerase alpha subunit B | 73 | 10 mg F / L |
| Q6PFE3 | DNA repair and recombination protein RAD54B | 158 | 10 mg F / L |
| O35134 | DNA-directed RNA polymerase I subunit RPA1 | 100 | 10 mg F / L |
| Q921X6 | DNA-directed RNA polymerase III subunit RPC6 | 176 | 10 mg F / L |
| Q9R022 | DnaJ homolog subfamily C member 12 | 289 | 10 mg F / L |
| Q05AA6 | Dystrophin-related protein 2 | 71 | 10 mg F / L |
| Q5DTM8 | E3 ubiquitin-protein ligase BRE1A | 107 | 10 mg F / L |
| Q8R516 | E3 ubiquitin-protein ligase MIB2 | 72 | 10 mg F / L |
| Q9QXK2 | E3 ubiquitin-protein ligase RAD18 | 72 | 10 mg F / L |
| Q8R0K2 | E3 ubiquitin-protein ligase TRIM31 | 60 | 10 mg F / L |
| Q8C7M3 | E3 ubiquitin-protein ligase TRIM9 | 108 | 10 mg F / L |
| Q8K0G5 | EARP-interacting protein | 103 | 10 mg F / L |
| Q3UVK0 | Endoplasmic reticulum metallopeptidase 1 | 62 | 10 mg F / L |
| Q9D1Q6 | Endoplasmic reticulum resident protein 44 | 85 | 10 mg F / L |
| P42125 | Enoyl-CoA delta isomerase 1, mitochondrial | 167 | 10 mg F / L |
| O54839 | Eomesodermin homolog | 99 | 10 mg F / L |
| Q3UYR4 | Espin-like protein | 64 | 10 mg F / L |
| Q8JZQ9 | Eukaryotic translation initiation factor 3 subunit B | 73 | 10 mg F / L |
| Q9QZ11 | Exonuclease 1 | 177 | 10 mg F / L |
| Q08943 | FACT complex subunit SSRP1 | 79 | 10 mg F / L |
| Q3UQN2 | F-BAR domain only protein 2 | 251 | 10 mg F / L |
| Q9EPX5 | F-box/LRR-repeat protein 12 | 153 | 10 mg F / L |
| Q8BHD4 | FERM domain-containing protein 3 | 74 | 10 mg F / L |
| Q8K0E8 | Fibrinogen beta chain | 290 | 10 mg F / L |
| Q8BUR3 | Forkhead box protein J3 | 99 | 10 mg F / L |
| Q61091 | Frizzled-8 | 84 | 10 mg F / L |
| Q810T2 | G2/mitotic-specific cyclin-B3 | 73 | 10 mg F / L |
| P13020 | Gelsolin | 238 | 10 mg F / L |
| Q8BL74 | General transcription factor 3C polypeptide 2 | 128 | 10 mg F / L |
| P97324 | Glucose-6-phosphate 1-dehydrogenase 2 | 152 | 10 mg F / L |
| Q01097 | Glutamate receptor ionotropic, NMDA 2B | 107 | 10 mg F / L |
| P19639 | Glutathione S-transferase Mu 3 | 84 | 10 mg F / L |
| Q3ULJ0 | Glycerol-3-phosphate dehydrogenase 1-like protein | 212 | 10 mg F / L |
| Q8CHP8 | Glycerol-3-phosphate phosphatase | 70 | 10 mg F / L |
| Q9ET01 | Glycogen phosphorylase, liver form | 91 | 10 mg F / L |
| Q9WUB3 | Glycogen phosphorylase, muscle form | 126 | 10 mg F / L |
| Q921M4 | Golgin subfamily A member 2 | 205 | 10 mg F / L |
| Q8K3J9 | G-protein coupled receptor family C group 5 member C | 951 | 10 mg F / L |
| Q80TI0 | GRAM domain-containing protein 1B | 87 | 10 mg F / L |
| Q03160 | Growth factor receptor-bound protein 7 | 80 | 10 mg F / L |
| Q61820 | GTP-binding nuclear protein Ran, testis-specific isoform | 67 | 10 mg F / L |
| P36536 | GTP-binding protein SAR1a | 256 | 10 mg F / L |
| Q9CQC9 | GTP-binding protein SAR1b | 174 | 10 mg F / L |
| Q9R0C8 | Guanine nucleotide exchange factor VAV3 | 78 | 10 mg F / L |
| P50149 | Guanine nucleotide-binding protein G(t) subunit alpha-2 | 79 | 10 mg F / L |
| Q3V3I2 | Guanine nucleotide-binding protein G(t) subunit alpha-3 | 105 | 10 mg F / L |
| P48722 | Heat shock 70 kDa protein 4L | 74 | 10 mg F / L |
| Q91X72 | Hemopexin | 155 | 10 mg F / L |
| Q8VEK3 | Heterogeneous nuclear ribonucleoprotein U | 102 | 10 mg F / L |
| P70349 | Histidine triad nucleotide-binding protein 1 | 541 | 10 mg F / L |
| P79457 | Histone demethylase UTY | 183 | 10 mg F / L |
| Q61188 | Histone-lysine N-methyltransferase EZH2 | 109 | 10 mg F / L |
| Q3U8K7 | Histone-lysine N-methyltransferase KMT5B | 286 | 10 mg F / L |
| Q8R1H0 | Homeodomain-only protein | 152 | 10 mg F / L |
| Q9JKY5 | Huntingtin-interacting protein 1-related protein | 117 | 10 mg F / L |
| Q2TPA8 | Hydroxysteroid dehydrogenase-like protein 2 | 116 | 10 mg F / L |
| P03975 | IgE-binding protein | 380 | 10 mg F / L |
| P15975 | Inactive ubiquitin carboxyl-terminal hydrolase 53 | 179 | 10 mg F / L |
| Q9D8Y8 | Inhibitor of growth protein 5 | 294 | 10 mg F / L |
| O88351 | Inhibitor of nuclear factor kappa-B kinase subunit beta | 125 | 10 mg F / L |
| A2ARA8 | Integrin alpha-8 | 92 | 10 mg F / L |
| Q80SU7 | Interferon-induced very large GTPase 1 | 133 | 10 mg F / L |
| P19182 | Interferon-related developmental regulator 1 | 147 | 10 mg F / L |
| Q6VH22 | Intraflagellar transport protein 172 homolog | 67 | 10 mg F / L |
| Q5DTN8 | Janus kinase and microtubule-interacting protein 3 | 72 | 10 mg F / L |
| Q62168 | Keratin, type I cuticular Ha2 | 134 | 10 mg F / L |
| Q497I4 | Keratin, type I cuticular Ha5 | 134 | 10 mg F / L |
| B1AQ75 | Keratin, type I cuticular Ha6 | 134 | 10 mg F / L |
| P02535 | Keratin, type I cytoskeletal 10 | 134 | 10 mg F / L |
| A1L317 | Keratin, type I cytoskeletal 24 | 134 | 10 mg F / L |
| A6BLY7 | Keratin, type I cytoskeletal 28 | 152 | 10 mg F / L |
| Q6IFX3 | Keratin, type I cytoskeletal 40 | 134 | 10 mg F / L |
| A2A9C3 | KICSTOR complex protein SZT2 | 66 | 10 mg F / L |
| O08672 | Kinesin-like protein KIFC2 | 92 | 10 mg F / L |
| Q8BX02 | KN motif and ankyrin repeat domain-containing protein 2 | 317 | 10 mg F / L |
| Q8BGA5 | KRR1 small subunit processome component homolog | 64 | 10 mg F / L |
| Q91WN4 | Kynurenine 3-monooxygenase | 96 | 10 mg F / L |
| Q80ST9 | Lebercilin | 75 | 10 mg F / L |
| Q8C0R9 | Leucine-rich repeat and death domain-containing protein 1 | 210 | 10 mg F / L |
| Q922Q8 | Leucine-rich repeat-containing protein 59 | 336 | 10 mg F / L |
| Q8BFW7 | Lipoma-preferred partner homolog | 76 | 10 mg F / L |
| Q61805 | Lipopolysaccharide-binding protein | 144 | 10 mg F / L |
| O35711 | Liprin-beta-2 | 139 | 10 mg F / L |
| P16125 | L-lactate dehydrogenase B chain | 355 | 10 mg F / L |
| P00342 | L-lactate dehydrogenase C chain | 202 | 10 mg F / L |
| Q99MN1 | Lysine--tRNA ligase | 128 | 10 mg F / L |
| P70699 | Lysosomal alpha-glucosidase | 134 | 10 mg F / L |
| Q62190 | Macrophage-stimulating protein receptor | 73 | 10 mg F / L |
| Q0PMG2 | MAM domain-containing glycosylphosphatidylinositol anchor protein 1 | 74 | 10 mg F / L |
| Q924M7 | Mannose-6-phosphate isomerase | 141 | 10 mg F / L |
| P70669 | Metalloendopeptidase homolog PEX | 168 | 10 mg F / L |
| Q3TY92 | Methyl-CpG-binding domain protein 6 | 154 | 10 mg F / L |
| L0HN04 | Methylcytosine dioxygenase TET3 | 67 | 10 mg F / L |
| P16332 | Methylmalonyl-CoA mutase, mitochondrial | 90 | 10 mg F / L |
| Q922T2 | Microfibril-associated glycoprotein 3 | 210 | 10 mg F / L |
| P10637 | Microtubule-associated protein tau | 96 | 10 mg F / L |
| Q80Y86 | Mitogen-activated protein kinase 15 | 149 | 10 mg F / L |
| F7BJB9 | MORC family CW-type zinc finger protein 3 | 104 | 10 mg F / L |
| Q80WJ6 | Multidrug resistance-associated protein 9 | 63 | 10 mg F / L |
| A6H6E2 | Multimerin-2 | 94 | 10 mg F / L |
| Q5RJH2 | Multiple C2 and transmembrane domain-containing protein 2 | 59 | 10 mg F / L |
| Q8K5B2 | Multiple coagulation factor deficiency protein 2 homolog | 1254 | 10 mg F / L |
| Q61006 | Muscle, skeletal receptor tyrosine-protein kinase | 75 | 10 mg F / L |
| Q80YT7 | Myomegalin | 129 | 10 mg F / L |
| Q6URW6 | Myosin-14 | 116 | 10 mg F / L |
| Q8BMF3 | NADP-dependent malic enzyme, mitochondrial | 100 | 10 mg F / L |
| Q4FZC9 | Nesprin-3 | 63 | 10 mg F / L |
| P21661 | Neuroendocrine convertase 2 | 155 | 10 mg F / L |
| Q99K10 | Neuroligin-1 | 94 | 10 mg F / L |
| Q8BM65 | Neuronal tyrosine-phosphorylated phosphoinositide-3-kinase adapter 2 | 69 | 10 mg F / L |
| P97333 | Neuropilin-1 | 87 | 10 mg F / L |
| Q8BVW0 | Neutral alpha-glucosidase C | 62 | 10 mg F / L |
| P10493 | Nidogen-1 | 102 | 10 mg F / L |
| P29477 | Nitric oxide synthase, inducible | 82 | 10 mg F / L |
| Q3UP24 | NLR family CARD domain-containing protein 4 | 63 | 10 mg F / L |
| Q9CZA6 | Nuclear distribution protein nudE homolog 1 | 192 | 10 mg F / L |
| Q8R0G9 | Nuclear pore complex protein Nup133 | 54 | 10 mg F / L |
| Q9WU42 | Nuclear receptor corepressor 2 | 242 | 10 mg F / L |
| Q6PIP5 | NudC domain-containing protein 1 | 79 | 10 mg F / L |
| P34983 | Olfactory receptor 1537 | 140 | 10 mg F / L |
| P29758 | Ornithine aminotransferase, mitochondrial | 74 | 10 mg F / L |
| A2AJ88 | Patatin-like phospholipase domain-containing protein 7 | 204 | 10 mg F / L |
| B9EJ80 | PDZ domain-containing protein 8 | 66 | 10 mg F / L |
| Q64378 | Peptidyl-prolyl cis-trans isomerase FKBP5 | 142 | 10 mg F / L |
| O54943 | Period circadian protein homolog 2 | 84 | 10 mg F / L |
| Q62009 | Periostin | 138 | 10 mg F / L |
| P15331 | Peripherin | 119 | 10 mg F / L |
| Q9Z280 | Phospholipase D1 | 121 | 10 mg F / L |
| P97813 | Phospholipase D2 | 321 | 10 mg F / L |
| Q9Z2M7 | Phosphomannomutase 2 | 188 | 10 mg F / L |
| Q8BLJ3 | PI-PLC X domain-containing protein 3 | 125 | 10 mg F / L |
| Q9R0K7 | Plasma membrane calcium-transporting ATPase 2 | 67 | 10 mg F / L |
| Q8BHJ9 | Pre-mRNA-splicing factor SLU7 | 109 | 10 mg F / L |
| Q3UX83 | Probable inactive 1-aminocyclopropane-1-carboxylate synthase-like protein 2 | 110 | 10 mg F / L |
| Q69ZK6 | Probable JmjC domain-containing histone demethylation protein 2C | 82 | 10 mg F / L |
| O35129 | Prohibitin-2 | 191 | 10 mg F / L |
| Q91ZA3 | Propionyl-CoA carboxylase alpha chain, mitochondrial | 192 | 10 mg F / L |
| Q99MN9 | Propionyl-CoA carboxylase beta chain, mitochondrial | 110 | 10 mg F / L |
| Q8VCR7 | Protein ABHD14B | 513 | 10 mg F / L |
| Q9D6I7 | Protein FAM69A | 83 | 10 mg F / L |
| P33215 | Protein NEDD1 | 60 | 10 mg F / L |
| Q9D281 | Protein Noxp20 | 66 | 10 mg F / L |
| Q3UMT1 | Protein phosphatase 1 regulatory subunit 12C | 54 | 10 mg F / L |
| Q9QZ67 | Protein phosphatase 1D | 102 | 10 mg F / L |
| Q8BVT6 | Protein phosphatase 2C-like domain-containing protein 1 | 112 | 10 mg F / L |
| Q99K43 | Protein regulator of cytokinesis 1 | 68 | 10 mg F / L |
| P14069 | Protein S100-A6 | 368 | 10 mg F / L |
| Q91XT4 | Protein transport protein Sec16B | 81 | 10 mg F / L |
| Q99KD5 | Protein unc-45 homolog A | 52 | 10 mg F / L |
| Q8R1F5 | Putative hydroxypyruvate isomerase | 195 | 10 mg F / L |
| Q8BZ32 | Putative Polycomb group protein ASXL2 | 88 | 10 mg F / L |
| Q8K183 | Pyridoxal kinase | 258 | 10 mg F / L |
| Q80Y56 | Rabenosyn-5 | 140 | 10 mg F / L |
| Q9QUG9 | RAS guanyl-releasing protein 2 | 108 | 10 mg F / L |
| Q8C2K5 | RAS protein activator like-3 | 85 | 10 mg F / L |
| Q9Z268 | RasGAP-activating-like protein 1 | 58 | 10 mg F / L |
| B2RU80 | Receptor-type tyrosine-protein phosphatase beta | 54 | 10 mg F / L |
| Q05909 | Receptor-type tyrosine-protein phosphatase gamma | 75 | 10 mg F / L |
| Q9Z2H1 | Regulator of G-protein signaling 11 | 146 | 10 mg F / L |
| Q8BHF5 | Reticulon | 54 | 10 mg F / L |
| Q62148 | Retinal dehydrogenase 2 | 145 | 10 mg F / L |
| O35600 | Retinal-specific ATP-binding cassette transporter | 63 | 10 mg F / L |
| Q61599 | Rho GDP-dissociation inhibitor 2 | 354 | 10 mg F / L |
| A2AWP8 | Rho guanine nucleotide exchange factor 10-like protein | 64 | 10 mg F / L |
| Q8R4H2 | Rho guanine nucleotide exchange factor 12 | 238 | 10 mg F / L |
| Q7TNR9 | Rho guanine nucleotide exchange factor 4 | 218 | 10 mg F / L |
| Q9CTN4 | Rho-related BTB domain-containing protein 3 | 89 | 10 mg F / L |
| P07742 | Ribonucleoside-diphosphate reductase large subunit | 144 | 10 mg F / L |
| P18653 | Ribosomal protein S6 kinase alpha-1 | 135 | 10 mg F / L |
| Q9WUT3 | Ribosomal protein S6 kinase alpha-2 | 173 | 10 mg F / L |
| P18654 | Ribosomal protein S6 kinase alpha-3 | 163 | 10 mg F / L |
| Q149F1 | RNA pseudouridylate synthase domain-containing protein 2 | 133 | 10 mg F / L |
| Q9QX96 | Sal-like protein 2 | 76 | 10 mg F / L |
| Q3URD3 | Sarcolemmal membrane-associated protein | 184 | 10 mg F / L |
| P42208 | Septin-2 | 108 | 10 mg F / L |
| P84104 | Serine/arginine-rich splicing factor 3 | 123 | 10 mg F / L |
| P83741 | Serine/threonine-protein kinase WNK1 | 58 | 10 mg F / L |
| Q80W00 | Serine/threonine-protein phosphatase 1 regulatory subunit 10 | 146 | 10 mg F / L |
| P19324 | Serpin H1 | 86 | 10 mg F / L |
| Q8BMC3 | SHC-transforming protein 2 | 135 | 10 mg F / L |
| Q91Y57 | Sialic acid-binding Ig-like lectin 12 | 63 | 10 mg F / L |
| Q9JMH7 | Sialidase-3 | 107 | 10 mg F / L |
| Q6ZWQ7 | Signal peptidase complex subunit 3 | 141 | 10 mg F / L |
| P47758 | Signal recognition particle receptor subunit beta | 81 | 10 mg F / L |
| P42225 | Signal transducer and activator of transcription 1 | 134 | 10 mg F / L |
| Q8BHY8 | Sorting nexin-14 | 81 | 10 mg F / L |
| Q7TME2 | Sperm-associated antigen 5 | 64 | 10 mg F / L |
| Q9CZV5 | STAGA complex 65 subunit gamma | 95 | 10 mg F / L |
| F6XZJ7 | Sterile alpha motif domain-containing protein 15 | 78 | 10 mg F / L |
| Q62465 | Synaptic vesicle membrane protein VAT-1 homolog | 186 | 10 mg F / L |
| Q62209 | Synaptonemal complex protein 1 | 97 | 10 mg F / L |
| Q8R570 | Synaptosomal-associated protein 47 | 119 | 10 mg F / L |
| Q9R0N4 | Synaptotagmin-10 | 99 | 10 mg F / L |
| Q71LX4 | Talin-2 | 89 | 10 mg F / L |
| Q3UES3 | Tankyrase-2 | 70 | 10 mg F / L |
| Q8BM85 | TBC domain-containing protein kinase-like protein | 63 | 10 mg F / L |
| P80316 | T-complex protein 1 subunit epsilon | 146 | 10 mg F / L |
| P42932 | T-complex protein 1 subunit theta | 126 | 10 mg F / L |
| P61406 | Telomerase-binding protein EST1A | 88 | 10 mg F / L |
| Q8CC21 | Tetratricopeptide repeat protein 19, mitochondrial | 139 | 10 mg F / L |
| Q8BH58 | TIP41-like protein | 100 | 10 mg F / L |
| P70191 | TNF receptor-associated factor 5 | 108 | 10 mg F / L |
| Q93092 | Transaldolase | 293 | 10 mg F / L |
| O55201 | Transcription elongation factor SPT5 | 119 | 10 mg F / L |
| Q91YD4 | Transient receptor potential cation channel subfamily M member 2 | 110 | 10 mg F / L |
| Q9D4D4 | Transketolase-like protein 2 | 131 | 10 mg F / L |
| Q8BH24 | Transmembrane 9 superfamily member 4 | 227 | 10 mg F / L |
| P20801 | Troponin C, skeletal muscle | 139 | 10 mg F / L |
| Q99NB8 | Ubiquilin-4 | 226 | 10 mg F / L |
| O88329 | Unconventional myosin-Ia | 118 | 10 mg F / L |
| P46735 | Unconventional myosin-Ib | 125 | 10 mg F / L |
| Q5SYD0 | Unconventional myosin-Id | 58 | 10 mg F / L |
| E9Q634 | Unconventional myosin-Ie | 77 | 10 mg F / L |
| Q9QZZ4 | Unconventional myosin-XV | 40 | 10 mg F / L |
| Q2QI47 | Usherin | 112 | 10 mg F / L |
| Q9Z1Q9 | Valine--tRNA ligase | 101 | 10 mg F / L |
| P49766 | Vascular endothelial growth factor B | 111 | 10 mg F / L |
| Q62059 | Versican core protein | 112 | 10 mg F / L |
| P62814 | V-type proton ATPase subunit B, brain isoform | 98 | 10 mg F / L |
| Q9JHY3 | WAP four-disulfide core domain protein 12 | 1089 | 10 mg F / L |
| O88342 | WD repeat-containing protein 1 | 77 | 10 mg F / L |
| O88532 | Zinc finger RNA-binding protein | 64 | 10 mg F / L |
| Q64433 | 10 kDa heat shock protein, mitochondrial | 198 | control |
| Q9CQ62 | 2,4-dienoyl-CoA reductase, mitochondrial | 155 | control |
| Q9Z0S1 | 3'(2'),5'-bisphosphate nucleotidase 1 | 142 | control |
| P63325 | 40S ribosomal protein S10 | 319 | control |
| P63323 | 40S ribosomal protein S12 | 257 | control |
| P62264 | 40S ribosomal protein S14 | 693 | control |
| P63276 | 40S ribosomal protein S17 | 389 | control |
| Q6ZWU9 | 40S ribosomal protein S27 | 707 | control |
| Q6ZWY3 | 40S ribosomal protein S27-like | 707 | control |
| Q9D110 | 5-formyltetrahydrofolate cyclo-ligase | 120 | control |
| Q6ZWV3 | 60S ribosomal protein L10 | 224 | control |
| P86048 | 60S ribosomal protein L10-like | 224 | control |
| P62900 | 60S ribosomal protein L31 | 338 | control |
| Q9D8M4 | 60S ribosomal protein L7-like 1 | 72 | control |
| P51410 | 60S ribosomal protein L9 | 490 | control |
| Q7TT18 | Activating transcription factor 7-interacting protein 1 | 231 | control |
| Q80XL6 | Acyl-CoA dehydrogenase family member 11 | 78 | control |
| Q8BQS5 | Adiponectin receptor protein 2 | 157 | control |
| Q8BMI3 | ADP-ribosylation factor-binding protein GGA3 | 65 | control |
| Q9QZQ1 | Afadin | 123 | control |
| E9Q3E1 | Aldehyde dehydrogenase family 3 member B2 | 131 | control |
| Q9QXJ1 | Amyloid-beta A4 precursor protein-binding family B member 1 | 234 | control |
| D3Z3C6 | AN1-type zinc finger protein 4 | 83 | control |
| G3UZ78 | Androglobin | 112 | control |
| Q80V94 | AP-4 complex subunit epsilon-1 | 132 | control |
| Q00623 | Apolipoprotein A-I | 289 | control |
| P09813 | Apolipoprotein A-II | 1176 | control |
| Q9Z0X1 | Apoptosis-inducing factor 1, mitochondrial | 77 | control |
| Q9WTY4 | Aquaporin-5 | 254 | control |
| Q8BVF9 | Archaemetzincin-1 | 70 | control |
| Q91WU5 | Arsenite methyltransferase | 96 | control |
| Q6HA09 | Astacin-like metalloendopeptidase | 70 | control |
| Q91YH5 | Atlastin-3 | 175 | control |
| Q91V24 | ATP-binding cassette sub-family A member 7 | 73 | control |
| Q9Z2H5 | Band 4.1-like protein 1 | 76 | control |
| Q8BHT6 | Beta-1,3-glucosyltransferase | 83 | control |
| P28653 | Biglycan | 84 | control |
| A2AHJ4 | Bromodomain and WD repeat-containing protein 3 | 256 | control |
| Q80XR2 | Calcium-transporting ATPase type 2C member 1 | 92 | control |
| P35564 | Calnexin | 162 | control |
| O35350 | Calpain-1 catalytic subunit | 87 | control |
| P12367 | cAMP-dependent protein kinase type II-alpha regulatory subunit | 125 | control |
| P13634 | Carbonic anhydrase 1 | 132 | control |
| P16015 | Carbonic anhydrase 3 | 1706 | control |
| Q9WVJ3 | Carboxypeptidase Q | 88 | control |
| Q6ZQ08 | CCR4-NOT transcription complex subunit 1 | 175 | control |
| Q8CB62 | Centrobin | 131 | control |
| Q5SW19 | Clustered mitochondria protein homolog | 63 | control |
| Q9QXK3 | Coatomer subunit gamma-2 | 122 | control |
| Q9DA73 | Coiled-coil domain-containing protein 89 | 69 | control |
| Q5SU73 | Coilin | 133 | control |
| Q9QZS0 | Collagen alpha-3(IV) chain | 354 | control |
| Q9Z160 | Conserved oligomeric Golgi complex subunit 1 | 165 | control |
| P07310 | Creatine kinase M-type | 328 | control |
| P11440 | Cyclin-dependent kinase 1 | 245 | control |
| Q9JM84 | Cystatin 10 | 482 | control |
| Q61753 | D-3-phosphoglycerate dehydrogenase | 81 | control |
| Q8CIQ7 | Dedicator of cytokinesis protein 3 | 123 | control |
| Q3U1T9 | DENN domain-containing protein 1B | 75 | control |
| Q8C4S8 | DENN domain-containing protein 2A | 109 | control |
| Q60710 | Deoxynucleoside triphosphate triphosphohydrolase SAMHD1 | 145 | control |
| Q7M6Y5 | Deuterosome assembly protein 1 | 113 | control |
| P31428 | Dipeptidase 1 | 302 | control |
| Q9JIC3 | DNA cross-link repair 1A protein | 75 | control |
| Q64511 | DNA topoisomerase 2-beta | 104 | control |
| Q60611 | DNA-binding protein SATB1 | 107 | control |
| Q9D7M8 | DNA-directed RNA polymerase II subunit RPB4 | 214 | control |
| Q91YQ5 | Dolichyl-diphosphooligosaccharide--protein glycosyltransferase subunit 1 | 363 | control |
| Q6XUX1 | Dual serine/threonine and tyrosine protein kinase | 126 | control |
| Q8BZ98 | Dynamin-3 | 134 | control |
| Q8BM54 | E3 ubiquitin-protein ligase MYLIP | 78 | control |
| Q9WTV7 | E3 ubiquitin-protein ligase RLIM | 75 | control |
| Q7TNG5 | Echinoderm microtubule-associated protein-like 2 | 88 | control |
| Q8K0L2 | Ectonucleoside triphosphate diphosphohydrolase 8 | 285 | control |
| Q60902 | Epidermal growth factor receptor substrate 15-like 1 | 92 | control |
| Q99JW5 | Epithelial cell adhesion molecule | 250 | control |
| Q6GQV7 | Erythroid differentiation-related factor 1 | 251 | control |
| Q9D172 | ES1 protein homolog, mitochondrial | 366 | control |
| Q91V76 | Ester hydrolase C11orf54 homolog | 86 | control |
| Q8R1B4 | Eukaryotic translation initiation factor 3 subunit C | 66 | control |
| Q9WUK2 | Eukaryotic translation initiation factor 4H | 170 | control |
| O35250 | Exocyst complex component 7 | 132 | control |
| Q6P5F9 | Exportin-1 | 231 | control |
| Q8R123 | FAD synthase | 94 | control |
| Q3TQB2 | FAD-dependent oxidoreductase domain-containing protein 1 | 90 | control |
| Q3TDN2 | FAS-associated factor 2 | 110 | control |
| P19096 | Fatty acid synthase | 66 | control |
| P04117 | Fatty acid-binding protein, adipocyte | 143 | control |
| Q9CQ24 | F-box only protein 36 | 201 | control |
| Q8BIE6 | FERM domain-containing protein 4A | 95 | control |
| E9PV24 | Fibrinogen alpha chain | 77 | control |
| Q8VCM7 | Fibrinogen gamma chain | 109 | control |
| O70497 | Ficolin-2 | 105 | control |
| Q9R216 | Frizzled-9 | 78 | control |
| Q3U2I3 | FTS and Hook-interacting protein | 99 | control |
| Q5SNZ0 | Girdin | 98 | control |
| P26443 | Glutamate dehydrogenase 1, mitochondrial | 115 | control |
| P15105 | Glutamine synthetase | 146 | control |
| O35660 | Glutathione S-transferase Mu 6 | 198 | control |
| Q9JJI6 | GPI ethanolamine phosphate transferase 3 | 100 | control |
| Q8VEL9 | GTP-binding protein REM 2 | 195 | control |
| P36916 | Guanine nucleotide-binding protein-like 1 | 103 | control |
| Q6TL19 | Guanylate cyclase 2G | 81 | control |
| Q8K0U4 | Heat shock 70 kDa protein 12A | 161 | control |
| Q8BM72 | Heat shock 70 kDa protein 13 | 255 | control |
| Q99020 | Heterogeneous nuclear ribonucleoprotein A/B | 412 | control |
| Q9Z130 | Heterogeneous nuclear ribonucleoprotein D-like | 119 | control |
| Q9D0E1 | Heterogeneous nuclear ribonucleoprotein M | 391 | control |
| Q9WVG6 | Histone-arginine methyltransferase CARM1 | 276 | control |
| P55200 | Histone-lysine N-methyltransferase 2A | 109 | control |
| P09026 | Homeobox protein Hox-B3 | 145 | control |
| Q9ESM3 | Hyaluronan and proteoglycan link protein 2 | 141 | control |
| P01864 | Ig gamma-2A chain C region secreted form | 205 | control |
| Q9DBZ1 | Inhibitor of nuclear factor kappa-B kinase-interacting protein | 98 | control |
| Q8CIM8 | Integrator complex subunit 4 | 181 | control |
| Q9QXH4 | Integrin alpha-X | 127 | control |
| Q5DU25 | IQ motif and SEC7 domain-containing protein 2 | 70 | control |
| Q9DBJ6 | Josephin-1 | 128 | control |
| Q80W68 | Kin of IRRE-like protein 1 | 107 | control |
| P33173 | Kinesin-like protein KIF1A | 117 | control |
| Q7TNC6 | Kinesin-like protein KIF26B | 66 | control |
| Q8K1F9 | Lactase-like protein | 132 | control |
| O89112 | LanC-like protein 1 | 162 | control |
| Q9EQR5 | Lck-interacting transmembrane adapter 1 | 119 | control |
| W8DXL4 | Leucine-rich repeat, immunoglobulin-like domain and transmembrane domain-containing protein 3 | 75 | control |
| P51174 | Long-chain specific acyl-CoA dehydrogenase, mitochondrial | 211 | control |
| P51885 | Lumican | 106 | control |
| P59997 | Lysine-specific demethylase 2A | 28 | control |
| O35954 | Membrane-associated phosphatidylinositol transfer protein 1 | 85 | control |
| P20357 | Microtubule-associated protein 2 | 114 | control |
| P28665 | Murinoglobulin-1 | 57 | control |
| P28666 | Murinoglobulin-2 | 66 | control |
| Q5SUV2 | MYCBP-associated protein | 92 | control |
| P97457 | Myosin regulatory light chain 2, skeletal muscle isoform | 1320 | control |
| P13541 | Myosin-3 | 223 | control |
| Q5SX39 | Myosin-4 | 468 | control |
| Q02566 | Myosin-6 | 190 | control |
| Q91Z83 | Myosin-7 | 184 | control |
| A2AQP0 | Myosin-7B | 138 | control |
| P13542 | Myosin-8 | 381 | control |
| Q9Z2C9 | Myotubularin-related protein 7 | 102 | control |
| Q99LC3 | NADH dehydrogenase [ubiquinone] 1 alpha subcomplex subunit 10, mitochondrial | 1049 | control |
| Q91WD5 | NADH dehydrogenase [ubiquinone] iron-sulfur protein 2, mitochondrial | 75 | control |
| Q9CXZ1 | NADH dehydrogenase [ubiquinone] iron-sulfur protein 4, mitochondrial | 92 | control |
| Q8K1S3 | Netrin receptor UNC5B | 94 | control |
| Q62443 | Neuronal pentraxin-1 | 105 | control |
| Q3TRM4 | Neuropathy target esterase | 336 | control |
| P42580 | NK1 transcription factor-related protein 2 | 115 | control |
| Q8C163 | Nuclease EXOG, mitochondrial | 332 | control |
| Q9CZ30 | Obg-like ATPase 1 | 75 | control |
| P32848 | Parvalbumin alpha | 932 | control |
| Q3URU2 | Paternally-expressed gene 3 protein | 194 | control |
| P58501 | PAX3- and PAX7-binding protein 1 | 122 | control |
| Q9WUA2 | Phenylalanine--tRNA ligase beta subunit | 159 | control |
| Q8VEM8 | Phosphate carrier protein, mitochondrial | 294 | control |
| P97350 | Plakophilin-1 | 201 | control |
| G5E829 | Plasma membrane calcium-transporting ATPase 1 | 65 | control |
| Q9CY58 | Plasminogen activator inhibitor 1 RNA-binding protein | 202 | control |
| Q99K51 | Plastin-3 | 79 | control |
| Q3UH93 | Plexin-D1 | 100 | control |
| Q6PCL9 | Poly(A) polymerase gamma | 238 | control |
| Q91VA6 | Polymerase delta-interacting protein 2 | 80 | control |
| P17225 | Polypyrimidine tract-binding protein 1 | 421 | control |
| Q501J6 | Probable ATP-dependent RNA helicase DDX17 | 104 | control |
| Q6PDI5 | Proteasome-associated protein ECM29 homolog | 115 | control |
| Q9DAN1 | Protein disulfide-isomerase-like protein of the testis | 132 | control |
| Q8C729 | Protein FAM126B | 109 | control |
| Q8BHZ0 | Protein FAM49A | 130 | control |
| Q8C753 | Protein KIAA0556 | 58 | control |
| Q7TPM1 | Protein PRRC2B | 100 | control |
| Q8C8N2 | Protein SCAI | 206 | control |
| Q08642 | Protein-arginine deiminase type-2 | 61 | control |
| P23492 | Purine nucleoside phosphorylase | 167 | control |
| Q8VIG3 | Radial spoke head 1 homolog | 75 | control |
| Q68EF8 | Rap guanine nucleotide exchange factor-like 1 | 121 | control |
| P61027 | Ras-related protein Rab-10 | 2350 | control |
| P35283 | Ras-related protein Rab-12 | 1028 | control |
| Q9DD03 | Ras-related protein Rab-13 | 1284 | control |
| Q91V41 | Ras-related protein Rab-14 | 1163 | control |
| Q8K386 | Ras-related protein Rab-15 | 2369 | control |
| P62821 | Ras-related protein Rab-1A | 2476 | control |
| Q9D1G1 | Ras-related protein Rab-1B | 2366 | control |
| Q504M8 | Ras-related protein Rab-26 | 999 | control |
| Q923S9 | Ras-related protein Rab-30 | 999 | control |
| O35963 | Ras-related protein Rab-33B | 1715 | control |
| Q6PHN9 | Ras-related protein Rab-35 | 2107 | control |
| Q9JKM7 | Ras-related protein Rab-37 | 999 | control |
| Q8BHD0 | Ras-related protein Rab-39A | 1621 | control |
| Q8BHC1 | Ras-related protein Rab-39B | 999 | control |
| P63011 | Ras-related protein Rab-3A | 999 | control |
| Q9CZT8 | Ras-related protein Rab-3B | 999 | control |
| P62823 | Ras-related protein Rab-3C | 999 | control |
| P35276 | Ras-related protein Rab-3D | 999 | control |
| Q8CG50 | Ras-related protein Rab-43 | 999 | control |
| P56371 | Ras-related protein Rab-4A | 999 | control |
| Q91ZR1 | Ras-related protein Rab-4B | 999 | control |
| P35279 | Ras-related protein Rab-6A | 1606 | control |
| P61294 | Ras-related protein Rab-6B | 1606 | control |
| P55258 | Ras-related protein Rab-8A | 2282 | control |
| P61028 | Ras-related protein Rab-8B | 2282 | control |
| Q09PK2 | Retroviral-like aspartic protease 1 | 104 | control |
| Q9CXK9 | RNA-binding protein 33 | 95 | control |
| Q8BHW9 | Schlafen-like protein 1 | 93 | control |
| Q8C0C4 | Serine-rich coiled-coil domain-containing protein 1 | 137 | control |
| P52430 | Serum paraoxonase/arylesterase 1 | 127 | control |
| Q8BH49 | Sesquipedalian-1 | 341 | control |
| Q9JM90 | Signal-transducing adaptor protein 1 | 233 | control |
| Q91Z67 | SLIT-ROBO Rho GTPase-activating protein 2 | 180 | control |
| Q3UZP4 | Small VCP/p97-interacting protein | 136 | control |
| Q62417 | Sorbin and SH3 domain-containing protein 1 | 55 | control |
| O55183 | Stanniocalcin-1 | 114 | control |
| Q9Z2I8 | Succinate--CoA ligase [GDP-forming] subunit beta, mitochondrial | 134 | control |
| Q9CPR7 | Suppressor of IKBKE 1 | 328 | control |
| O70439 | Syntaxin-7 | 110 | control |
| Q3UKC1 | Tax1-binding protein 1 homolog | 122 | control |
| P80315 | T-complex protein 1 subunit delta | 127 | control |
| Q8R3G9 | Tetraspanin-8 | 214 | control |
| P10639 | Thioredoxin | 571 | control |
| Q7TN22 | Thioredoxin domain-containing protein 16 | 94 | control |
| Q3U269 | Thiosulfate sulfurtransferase/rhodanese-like domain-containing protein 2 | 152 | control |
| P97770 | THUMP domain-containing protein 3 | 177 | control |
| Q60803 | TNF receptor-associated factor 3 | 70 | control |
| P70399 | TP53-binding protein 1 | 103 | control |
| Q8VE65 | Transcription initiation factor TFIID subunit 12 | 298 | control |
| Q7TN60 | Transmembrane channel-like protein 6 | 415 | control |
| Q8VHK8 | Transmembrane protease serine 11D | 128 | control |
| Q8BNV1 | tRNA (uracil-5-)-methyltransferase homolog A | 260 | control |
| P24529 | Tyrosine 3-monooxygenase | 327 | control |
| Q5I043 | Ubiquitin carboxyl-terminal hydrolase 28 | 61 | control |
| P62838 | Ubiquitin-conjugating enzyme E2 D2 | 480 | control |
| P61079 | Ubiquitin-conjugating enzyme E2 D3 | 480 | control |
| Q8VI16 | UDP-GlcNAc:betaGal beta-1,3-N-acetylglucosaminyltransferase 9 | 159 | control |
| Q63886 | UDP-glucuronosyltransferase 1-1 | 248 | control |
| P70691 | UDP-glucuronosyltransferase 1-2 | 248 | control |
| Q64435 | UDP-glucuronosyltransferase 1-6 | 248 | control |
| Q6ZQM8 | UDP-glucuronosyltransferase 1-7C | 248 | control |
| Q62452 | UDP-glucuronosyltransferase 1-9 | 249 | control |
| A1A535 | Ventricular zone-expressed PH domain-containing protein 1 | 81 | control |
| P50544 | Very long-chain specific acyl-CoA dehydrogenase, mitochondrial | 120 | control |
| Q6QD59 | Vesicle transport protein SEC20 | 121 | control |
| Q6PHS9 | Voltage-dependent calcium channel subunit alpha-2/delta-2 | 58 | control |
| Q6VNB8 | WD repeat and FYVE domain-containing protein 3 | 106 | control |
| Q91V09 | WD repeat-containing protein 13 | 93 | control |
| Q8R1D1 | Zinc finger protein 426 | 361 | control |
| Q7TSH3 | Zinc finger protein 516 | 56 | control |

^a^Identification is based on proteins ID from UniProt protein database, reviewed only (<http://www.uniprot.org/>).

^b^ Proteins with expression significantly altered are organized according to the ratio

*Indicates unique proteins in alphabetical order.

**Table S2**. Proteins with different expression significantly altered in the submandibular glands of mice exposed to 50 mg F/L vs. control

| ***^a^*Access Number** | **Protein name description** | **PLGS Score** | **^b^Ratio**  **50 mgF/ L: control** |
| --- | --- | --- | --- |
| Q9WVA4 | Transgelin-2 | 856 | 8.08 |
| Q3UNW5 | Transcription factor CP2-like protein 1 | 301 | 7.85 |
| P20108 | Thioredoxin-dependent peroxide reductase, mitochondrial | 513 | 4.14 |
| P26039 | Talin-1 | 405 | 2.56 |
| Q61900 | Submaxillary gland androgen-regulated protein 3A | 1611 | 2.25 |
| Q9WV27 | Sodium/potassium-transporting ATPase subunit alpha-4 | 143 | 2.18 |
| Q6PIC6 | Sodium/potassium-transporting ATPase subunit alpha-3 | 189 | 1.82 |
| Q6PIE5 | Sodium/potassium-transporting ATPase subunit alpha-2 | 189 | 1.70 |
| Q99J77 | Sialic acid synthase | 732 | 1.68 |
| Q8VDN2 | Sodium/potassium-transporting ATPase subunit alpha-1 | 333 | 1.68 |
| Q60854 | Serpin B6 | 3395 | 1.67 |
| P24549 | Retinal dehydrogenase 1 | 217 | 1.65 |
| Q99PT1 | Rho GDP-dissociation inhibitor 1 | 318 | 1.65 |
| P00796 | Renin-2 | 9834 | 1.63 |
| Q8VIG3 | Radial spoke head 1 homolog | 75 | 1.60 |
| P06281 | Renin-1 | 7733 | 1.60 |
| E9Q8I9 | Protein furry homolog | 84 | 1.58 |
| P17225 | Polypyrimidine tract-binding protein 1 | 421 | 1.55 |
| Q8R1F1 | Niban-like protein 1 | 204 | 1.54 |
| P00688 | Pancreatic alpha-amylase | 1706 | 1.54 |
| Q8C985 | Neurexin-3-beta | 75 | 1.49 |
| P34884 | Macrophage migration inhibitory factor | 838 | 1.48 |
| Q9CQ19 | Myosin regulatory light polypeptide 9 | 2961 | 1.48 |
| Q6P9K9 | Neurexin-3 | 122 | 1.48 |
| O09159 | Lysosomal alpha-mannosidase | 185 | 1.46 |
| Q6IFX2 | Keratin, type I cytoskeletal 42 | 55 | 1.45 |
| P15949 | Kallikrein 1-related peptidase b9 | 40806 | 1.43 |
| P07628 | Kallikrein 1-related peptidase b8 | 17164 | 1.40 |
| Q9JM71 | Kallikrein 1-related peptidase b27 | 18515 | 1.39 |
| P00756 | Kallikrein 1-related peptidase b3 | 13563 | 1.39 |
| P36369 | Kallikrein 1-related peptidase b26 | 37887 | 1.38 |
| Q61754 | Kallikrein 1-related peptidase b24 | 11318 | 1.35 |
| Q61759 | Kallikrein 1-related peptidase b21 | 21122 | 1.32 |
| P15948 | Kallikrein 1-related peptidase b22 | 40034 | 1.32 |
| P00755 | Kallikrein 1-related peptidase b1 | 24880 | 1.31 |
| P15946 | Kallikrein 1-related peptidase b11 | 14312 | 1.31 |
| P04071 | Kallikrein 1-related peptidase b16 | 16582 | 1.31 |
| Q99L20 | Glutathione S-transferase theta-3 | 89 | 1.28 |
| P01864 | Ig gamma-2A chain C region secreted form | 205 | 1.28 |
| P15626 | Glutathione S-transferase Mu 2 | 569 | 1.27 |
| P46425 | Glutathione S-transferase P 2 | 120 | 1.27 |
| P54818 | Galactocerebrosidase | 82 | 1.26 |
| Q9D8N0 | Elongation factor 1-gamma | 349 | 1.23 |
| P36368 | Epidermal growth factor-binding protein type B | 21370 | 1.23 |
| P97807 | Fumarate hydratase, mitochondrial | 343 | 1.23 |
| Q6P8J7 | Creatine kinase S-type, mitochondrial | 106 | 1.22 |
| P97315 | Cysteine and glycine-rich protein 1 | 456 | 1.22 |
| Q9CPY7 | Cytosol aminopeptidase | 462 | 1.22 |
| Q9DBG6 | Dolichyl-diphosphooligosaccharide--protein glycosyltransferase subunit 2 | 191 | 1.22 |
| O89079 | Coatomer subunit epsilon | 241 | 1.21 |
| Q04857 | Collagen alpha-1(VI) chain | 95 | 1.21 |
| P18242 | Cathepsin D | 776 | 1.20 |
| P24270 | Catalase | 341 | 1.19 |
| Q9WVJ3 | Carboxypeptidase Q | 88 | 1.17 |
| Q08091 | Calponin-1 | 368 | 1.16 |
| P05202 | Aspartate aminotransferase, mitochondrial | 288 | 1.14 |
| P56480 | ATP synthase subunit beta, mitochondrial | 5560 | 1.14 |
| P10107 | Annexin A1 | 173 | 1.13 |
| P14824 | Annexin A6 | 403 | 1.13 |
| P68134 | Actin, alpha skeletal muscle | 33054 | 1.12 |
| P62737 | Actin, aortic smooth muscle | 31651 | 1.12 |
| P45376 | Aldose reductase | 174 | 1.12 |
| P62889 | 60S ribosomal protein L30 | 983 | 1.11 |
| P62918 | 60S ribosomal protein L8 | 96 | 1.11 |
| Q91XA9 | Acidic mammalian chitinase | 795 | 1.11 |
| P68033 | Actin, alpha cardiac muscle 1 | 33613 | 1.11 |
| O70456 | 14-3-3 protein sigma | 999 | 1.07 |
| P63101 | 14-3-3 protein zeta/delta | 1446 | 1.07 |
| Q9CQV8 | 14-3-3 protein beta/alpha | 1112 | 1.04 |
| Q922F4 | Tubulin beta-6 chain | 8367 | 0.95 |
| P62983 | Ubiquitin-40S ribosomal protein S27a | 10219 | 0.95 |
| Q8VDJ3 | Vigilin | 233 | 0.95 |
| Q64727 | Vinculin | 103 | 0.95 |
| P99024 | Tubulin beta-5 chain | 10154 | 0.93 |
| Q9ERD7 | Tubulin beta-3 chain | 9451 | 0.92 |
| Q9D6F9 | Tubulin beta-4A chain | 1444 | 0.92 |
| P68372 | Tubulin beta-4B chain | 9733 | 0.92 |
| P68373 | Tubulin alpha-1C chain | 2012 | 0.91 |
| P05214 | Tubulin alpha-3 chain | 1185 | 0.91 |
| P68368 | Tubulin alpha-4A chain | 3534 | 0.91 |
| Q9JJZ2 | Tubulin alpha-8 chain | 914 | 0.91 |
| A2AQ07 | Tubulin beta-1 chain | 79 | 0.91 |
| Q7TMM9 | Tubulin beta-2A chain | 9950 | 0.91 |
| Q9CWF2 | Tubulin beta-2B chain | 9950 | 0.91 |
| Q7TN60 | Transmembrane channel-like protein 6 | 415 | 0.90 |
| Q8BMS1 | Trifunctional enzyme subunit alpha, mitochondrial | 231 | 0.90 |
| Q99JY0 | Trifunctional enzyme subunit beta, mitochondrial | 106 | 0.90 |
| P17751 | Triosephosphate isomerase | 6289 | 0.90 |
| P58774 | Tropomyosin beta chain | 1662 | 0.90 |
| P68369 | Tubulin alpha-1A chain | 2012 | 0.90 |
| P14094 | Sodium/potassium-transporting ATPase subunit beta-1 | 595 | 0.90 |
| P38647 | Stress-70 protein, mitochondrial | 93 | 0.90 |
| P09671 | Superoxide dismutase [Mn], mitochondrial | 700 | 0.90 |
| P80315 | T-complex protein 1 subunit delta | 127 | 0.90 |
| P97770 | THUMP domain-containing protein 3 | 177 | 0.90 |
| Q01853 | Transitional endoplasmic reticulum ATPase | 578 | 0.90 |
| P40142 | Transketolase | 711 | 0.90 |
| Q9Z1W8 | Potassium-transporting ATPase alpha chain 2 | 116 | 0.89 |
| Q61838 | Pregnancy zone protein | 1024 | 0.89 |
| P27773 | Protein disulfide-isomerase A3 | 2854 | 0.89 |
| Q8C729 | Protein FAM126B | 109 | 0.89 |
| Q68EF8 | Rap guanine nucleotide exchange factor-like 1 | 121 | 0.89 |
| Q921I1 | Serotransferrin | 2511 | 0.89 |
| P0CG50 | Polyubiquitin-C | 10219 | 0.88 |
| Q64436 | Potassium-transporting ATPase alpha chain 1 | 161 | 0.88 |
| Q01768 | Nucleoside diphosphate kinase B | 4265 | 0.87 |
| P17742 | Peptidyl-prolyl cis-trans isomerase A | 5192 | 0.87 |
| P24369 | Peptidyl-prolyl cis-trans isomerase B | 358 | 0.87 |
| P35700 | Peroxiredoxin-1 | 3271 | 0.87 |
| Q61171 | Peroxiredoxin-2 | 1227 | 0.87 |
| Q9DBJ1 | Phosphoglycerate mutase 1 | 565 | 0.87 |
| P29341 | Polyadenylate-binding protein 1 | 80 | 0.87 |
| P0CG49 | Polyubiquitin-B | 10219 | 0.87 |
| Q91VD9 | NADH-ubiquinone oxidoreductase 75 kDa subunit, mitochondrial | 97 | 0.86 |
| Q8K1S3 | Netrin receptor UNC5B | 94 | 0.86 |
| E9Q7X7 | Neurexin II | 77 | 0.86 |
| Q61937 | Nucleophosmin | 200 | 0.86 |
| P15532 | Nucleoside diphosphate kinase A | 2769 | 0.86 |
| Q9EQ20 | Methylmalonate-semialdehyde dehydrogenase [acylating], mitochondrial | 221 | 0.85 |
| P28665 | Murinoglobulin-1 | 57 | 0.85 |
| Q5SX39 | Myosin-4 | 468 | 0.85 |
| P13542 | Myosin-8 | 381 | 0.85 |
| Q8K1F9 | Lactase-like protein | 132 | 0.84 |
| Q9EQR5 | Lck-interacting transmembrane adapter 1 | 119 | 0.84 |
| P08249 | Malate dehydrogenase, mitochondrial | 5216 | 0.84 |
| Q8VED5 | Keratin, type II cytoskeletal 79 | 82 | 0.84 |
| P11679 | Keratin, type II cytoskeletal 8 | 3580 | 0.84 |
| Q80W68 | Kin of IRRE-like protein 1 | 107 | 0.84 |
| P07744 | Keratin, type II cytoskeletal 4 | 82 | 0.83 |
| P50446 | Keratin, type II cytoskeletal 6A | 118 | 0.83 |
| Q6IME9 | Keratin, type II cytoskeletal 72 | 452 | 0.83 |
| Q6IFZ9 | Keratin, type II cytoskeletal 74 | 142 | 0.83 |
| Q8BGZ7 | Keratin, type II cytoskeletal 75 | 332 | 0.83 |
| Q99M73 | Keratin, type II cuticular Hb4 | 114 | 0.82 |
| Q6IFZ6 | Keratin, type II cytoskeletal 1b | 386 | 0.82 |
| Q3TTY5 | Keratin, type II cytoskeletal 2 epidermal | 399 | 0.82 |
| Q3UV17 | Keratin, type II cytoskeletal 2 oral | 83 | 0.82 |
| Q9JKR6 | Hypoxia up-regulated protein 1 | 1164 | 0.81 |
| P01878 | Ig alpha chain C region | 515 | 0.81 |
| P54071 | Isocitrate dehydrogenase [NADP], mitochondrial | 211 | 0.81 |
| P15945 | Kallikrein 1-related peptidase b5 | 31682 | 0.81 |
| P00757 | Kallikrein 1-related peptidase-like b4 | 5700 | 0.81 |
| P15947 | Kallikrein-1 | 45282 | 0.81 |
| P84244 | Histone H3.3 | 1632 | 0.80 |
| P02301 | Histone H3.3C | 1632 | 0.80 |
| P62806 | Histone H4 | 16551 | 0.80 |
| Q8CGP2 | Histone H2B type 1-P | 7628 | 0.79 |
| Q64525 | Histone H2B type 2-B | 7628 | 0.79 |
| Q64524 | Histone H2B type 2-E | 5897 | 0.79 |
| Q9D2U9 | Histone H2B type 3-A | 5897 | 0.79 |
| Q8CGP0 | Histone H2B type 3-B | 5897 | 0.79 |
| P68433 | Histone H3.1 | 1632 | 0.79 |
| P84228 | Histone H3.2 | 1632 | 0.79 |
| Q64475 | Histone H2B type 1-B | 7628 | 0.79 |
| Q6ZWY9 | Histone H2B type 1-C/E/G | 7628 | 0.79 |
| P10853 | Histone H2B type 1-F/J/L | 7628 | 0.79 |
| Q64478 | Histone H2B type 1-H | 7628 | 0.79 |
| Q8CGP1 | Histone H2B type 1-K | 7628 | 0.79 |
| P10854 | Histone H2B type 1-M | 7628 | 0.79 |
| P27661 | Histone H2AX | 4710 | 0.78 |
| P70696 | Histone H2B type 1-A | 1098 | 0.78 |
| Q8BFU2 | Histone H2A type 3 | 30354 | 0.77 |
| Q3THW5 | Histone H2A.V | 5103 | 0.77 |
| P0C0S6 | Histone H2A.Z | 5103 | 0.77 |
| P01942 | Hemoglobin subunit alpha | 22924 | 0.76 |
| P02088 | Hemoglobin subunit beta-1 | 84334 | 0.76 |
| P02089 | Hemoglobin subunit beta-2 | 41267 | 0.76 |
| P06467 | Hemoglobin subunit zeta | 309 | 0.76 |
| Q8BG05 | Heterogeneous nuclear ribonucleoprotein A3 | 203 | 0.76 |
| P61979 | Heterogeneous nuclear ribonucleoprotein K | 1038 | 0.76 |
| Q8CGP7 | Histone H2A type 1-K | 30354 | 0.76 |
| Q64522 | Histone H2A type 2-B | 4024 | 0.76 |
| Q99LC5 | Electron transfer flavoprotein subunit alpha, mitochondrial | 1260 | 0.76 |
| P10126 | Elongation factor 1-alpha 1 | 23267 | 0.76 |
| P62631 | Elongation factor 1-alpha 2 | 3859 | 0.76 |
| P58252 | Elongation factor 2 | 699 | 0.76 |
| P08113 | Endoplasmin | 1411 | 0.76 |
| Q8BH95 | Enoyl-CoA hydratase, mitochondrial | 241 | 0.76 |
| Q8CHG3 | GRIP and coiled-coil domain-containing protein 2 | 186 | 0.76 |
| P11499 | Heat shock protein HSP 90-beta | 3260 | 0.76 |
| P56391 | Cytochrome c oxidase subunit 6B1 | 3587 | 0.75 |
| P62897 | Cytochrome c, somatic | 840 | 0.75 |
| Q9D0M3 | Cytochrome c1, heme protein, mitochondrial | 176 | 0.75 |
| Q8BIK4 | Dedicator of cytokinesis protein 9 | 154 | 0.75 |
| Q91YQ5 | Dolichyl-diphosphooligosaccharide--protein glycosyltransferase subunit 1 | 363 | 0.75 |
| Q9CZ13 | Cytochrome b-c1 complex subunit 1, mitochondrial | 277 | 0.74 |
| Q9DB77 | Cytochrome b-c1 complex subunit 2, mitochondrial | 296 | 0.74 |
| Q9CZU6 | Citrate synthase, mitochondrial | 399 | 0.72 |
| Q03402 | Cysteine-rich secretory protein 3 | 766 | 0.72 |
| P16015 | Carbonic anhydrase 3 | 1706 | 0.71 |
| Q02248 | Catenin beta-1 | 745 | 0.71 |
| P14211 | Calreticulin | 1792 | 0.70 |
| Q03265 | ATP synthase subunit alpha, mitochondrial | 6988 | 0.70 |
| Q9D3D9 | ATP synthase subunit delta, mitochondrial | 244 | 0.70 |
| Q9DB20 | ATP synthase subunit O, mitochondrial | 512 | 0.70 |
| Q9Z1P8 | Angiopoietin-related protein 4 | 117 | 0.69 |
| Q3UMR0 | Ankyrin repeat domain-containing protein 27 | 246 | 0.69 |
| O88990 | Alpha-actinin-3 | 105 | 0.68 |
| Q70FJ1 | A-kinase anchor protein 9 | 95 | 0.68 |
| P61750 | ADP-ribosylation factor 4 | 3413 | 0.67 |
| P84084 | ADP-ribosylation factor 5 | 3413 | 0.67 |
| P61205 | ADP-ribosylation factor 3 | 3776 | 0.62 |
| Q8BSL7 | ADP-ribosylation factor 2 | 3776 | 0.61 |
| P84078 | ADP-ribosylation factor 1 | 3776 | 0.60 |
| Q3V132 | ADP/ATP translocase 4 | 119 | 0.53 |
| P48962 | ADP/ATP translocase 1 | 227 | 0.51 |
| Q99KI0 | Aconitate hydratase, mitochondrial | 1986 | 0.49 |
| Q99NB1 | Acetyl-coenzyme A synthetase 2-like, mitochondrial | 165 | 0.44 |
| Q8QZT1 | Acetyl-CoA acetyltransferase, mitochondrial | 565 | 0.42 |
| P20029 | 78 kDa glucose-regulated protein | 3678 | 0.36 |
| P99027 | 60S acidic ribosomal protein P2 | 4026 | 0.35 |
| P14869 | 60S acidic ribosomal protein P0 | 1615 | 0.33 |
| P63038 | 60 kDa heat shock protein, mitochondrial | 214 | 0.30 |
| P62242 | 40S ribosomal protein S8 | 620 | 0.26 |
| P62702 | 40S ribosomal protein S4, X isoform | 316 | 0.19 |
| P25444 | 40S ribosomal protein S2 | 288 | 0.12 |
| Q05921 | 2-5A-dependent ribonuclease | 62 | 50 mgF/ L* |
| P26516 | 26S proteasome non-ATPase regulatory subunit 7 | 93 | 50 mgF/ L |
| P14148 | 60S ribosomal protein L7 | 78 | 50 mgF/ L |
| Q4JIM5 | Abelson tyrosine-protein kinase 2 | 123 | 50 mgF/ L |
| Q9EST5 | Acidic leucine-rich nuclear phosphoprotein 32 family member B | 205 | 50 mgF/ L |
| Q8CG27 | Actin-like protein 9 | 93 | 50 mgF/ L |
| Q5SSL4 | Active breakpoint cluster region-related protein | 129 | 50 mgF/ L |
| P31786 | Acyl-CoA-binding protein | 1023 | 50 mgF/ L |
| P50247 | Adenosylhomocysteinase | 152 | 50 mgF/ L |
| Q9WUR9 | Adenylate kinase 4, mitochondrial | 189 | 50 mgF/ L |
| Q60662 | A-kinase anchor protein 4 | 58 | 50 mgF/ L |
| P28474 | Alcohol dehydrogenase class-3 | 681 | 50 mgF/ L |
| P12023 | Amyloid-beta A4 protein | 74 | 50 mgF/ L |
| P19091 | Androgen receptor | 67 | 50 mgF/ L |
| Q99NH0 | Ankyrin repeat domain-containing protein 17 | 70 | 50 mgF/ L |
| Q5SUE8 | Ankyrin repeat domain-containing protein 40 | 67 | 50 mgF/ L |
| Q9QWY8 | Arf-GAP with SH3 domain, ANK repeat and PH domain-containing protein 1 | 92 | 50 mgF/ L |
| Q9D0I9 | Arginine--tRNA ligase, cytoplasmic | 65 | 50 mgF/ L |
| Q9CQQ7 | ATP synthase F(0) complex subunit B1, mitochondrial | 302 | 50 mgF/ L |
| O70133 | ATP-dependent RNA helicase A | 88 | 50 mgF/ L |
| O88967 | ATP-dependent zinc metalloprotease YME1L1 | 69 | 50 mgF/ L |
| P97477 | Aurora kinase A | 63 | 50 mgF/ L |
| P70444 | BH3-interacting domain death agonist | 267 | 50 mgF/ L |
| O35855 | Branched-chain-amino-acid aminotransferase, mitochondrial | 115 | 50 mgF/ L |
| P48754 | Breast cancer type 1 susceptibility protein homolog | 86 | 50 mgF/ L |
| Q8BFX3 | BTB/POZ domain-containing protein KCTD3 | 74 | 50 mgF/ L |
| Q922D8 | C-1-tetrahydrofolate synthase, cytoplasmic | 66 | 50 mgF/ L |
| Q6A068 | Cell division cycle 5-like protein | 49 | 50 mgF/ L |
| Q3UXL4 | Centrosomal protein kizuna | 105 | 50 mgF/ L |
| Q8BI22 | Centrosomal protein of 128 kDa | 264 | 50 mgF/ L |
| B2RX88 | Centrosome and spindle pole associated protein 1 | 171 | 50 mgF/ L |
| Q9D5D8 | Chromodomain Y-like protein 2 | 152 | 50 mgF/ L |
| Q9ESN9 | C-Jun-amino-terminal kinase-interacting protein 3 | 83 | 50 mgF/ L |
| Q8BRT1 | CLIP-associating protein 2 | 176 | 50 mgF/ L |
| Q3THF9 | Coenzyme Q-binding protein COQ10 homolog B, mitochondrial | 210 | 50 mgF/ L |
| Q4QRL3 | Coiled-coil domain-containing protein 88B | 70 | 50 mgF/ L |
| Q64739 | Collagen alpha-2(XI) chain | 42 | 50 mgF/ L |
| Q8R066 | Complement C1q tumor necrosis factor-related protein 4 | 88 | 50 mgF/ L |
| P06684 | Complement C5 | 52 | 50 mgF/ L |
| Q9JMB8 | Contactin-6 | 48 | 50 mgF/ L |
| Q8VE73 | Cullin-7 | 56 | 50 mgF/ L |
| P33267 | Cytochrome P450 2F2 | 68 | 50 mgF/ L |
| Q91WL5 | Cytochrome P450 4A12A | 65 | 50 mgF/ L |
| A2AF47 | Dedicator of cytokinesis protein 11 | 108 | 50 mgF/ L |
| A2RSQ0 | DENN domain-containing protein 5B | 84 | 50 mgF/ L |
| Q8R2M2 | Deoxynucleotidyltransferase terminal-interacting protein 2 | 48 | 50 mgF/ L |
| P49183 | Deoxyribonuclease-1 | 5966 | 50 mgF/ L |
| Q9D7J6 | Deoxyribonuclease-1-like 1 | 80 | 50 mgF/ L |
| Q91YP3 | Deoxyribose-phosphate aldolase | 86 | 50 mgF/ L |
| Q8BH86 | D-glutamate cyclase, mitochondrial | 56 | 50 mgF/ L |
| P97427 | Dihydropyrimidinase-related protein 1 | 184 | 50 mgF/ L |
| Q8BVG4 | Dipeptidyl peptidase 9 | 55 | 50 mgF/ L |
| Q9JJN0 | DNA polymerase eta | 51 | 50 mgF/ L |
| Q6PFE3 | DNA repair and recombination protein RAD54B | 159 | 50 mgF/ L |
| Q8BKF1 | DNA-directed RNA polymerase, mitochondrial | 48 | 50 mgF/ L |
| Q9CZ00 | Dysbindin domain-containing protein 1 | 177 | 50 mgF/ L |
| Q8CHI8 | E1A-binding protein p400 | 78 | 50 mgF/ L |
| Q9QXK2 | E3 ubiquitin-protein ligase RAD18 | 176 | 50 mgF/ L |
| Q8C7M3 | E3 ubiquitin-protein ligase TRIM9 | 95 | 50 mgF/ L |
| Q99MS7 | EH domain-binding protein 1-like protein 1 | 90 | 50 mgF/ L |
| Q60900 | ELAV-like protein 3 | 114 | 50 mgF/ L |
| Q61772 | Ephrin type-A receptor 7 | 66 | 50 mgF/ L |
| P42567 | Epidermal growth factor receptor substrate 15 | 61 | 50 mgF/ L |
| P50171 | Estradiol 17-beta-dehydrogenase 8 | 140 | 50 mgF/ L |
| Q9QZD9 | Eukaryotic translation initiation factor 3 subunit I | 90 | 50 mgF/ L |
| P22315 | Ferrochelatase, mitochondrial | 94 | 50 mgF/ L |
| Q8BX90 | Fibronectin type-III domain-containing protein 3A | 98 | 50 mgF/ L |
| Q8VHX6 | Filamin-C | 83 | 50 mgF/ L |
| Q6P9Q6 | FK506-binding protein 15 | 142 | 50 mgF/ L |
| P42128 | Forkhead box protein K1 | 70 | 50 mgF/ L |
| Q8VEB1 | G protein-coupled receptor kinase 5 | 63 | 50 mgF/ L |
| Q8BKN5 | Gamma-tubulin complex component 5 | 89 | 50 mgF/ L |
| P13020 | Gelsolin | 178 | 50 mgF/ L |
| P19639 | Glutathione S-transferase Mu 3 | 113 | 50 mgF/ L |
| Q3ULJ0 | Glycerol-3-phosphate dehydrogenase 1-like protein | 150 | 50 mgF/ L |
| Q9WUB3 | Glycogen phosphorylase, muscle form | 52 | 50 mgF/ L |
| P08752 | Guanine nucleotide-binding protein G(i) subunit alpha-2 | 111 | 50 mgF/ L |
| Q3V3I2 | Guanine nucleotide-binding protein G(t) subunit alpha-3 | 71 | 50 mgF/ L |
| Q9EQ15 | Guanine nucleotide-binding protein subunit beta-like protein 1 | 98 | 50 mgF/ L |
| Q61418 | H(+)/Cl(-) exchange transporter 4 | 372 | 50 mgF/ L |
| O08755 | Hepatocyte nuclear factor 6 | 86 | 50 mgF/ L |
| P51859 | Hepatoma-derived growth factor | 459 | 50 mgF/ L |
| Q9Z2X1 | Heterogeneous nuclear ribonucleoprotein F | 92 | 50 mgF/ L |
| Q8VEK3 | Heterogeneous nuclear ribonucleoprotein U | 56 | 50 mgF/ L |
| Q8BVE8 | Histone-lysine N-methyltransferase NSD2 | 46 | 50 mgF/ L |
| Q2TPA8 | Hydroxysteroid dehydrogenase-like protein 2 | 65 | 50 mgF/ L |
| Q7TQA1 | Immunoglobulin superfamily member 1 | 47 | 50 mgF/ L |
| Q61249 | Immunoglobulin-binding protein 1 | 81 | 50 mgF/ L |
| O88351 | Inhibitor of nuclear factor kappa-B kinase subunit beta | 118 | 50 mgF/ L |
| Q66JY2 | INO80 complex subunit D | 83 | 50 mgF/ L |
| Q9QUM0 | Integrin alpha-IIb | 53 | 50 mgF/ L |
| Q8R460 | Interleukin-36 gamma | 105 | 50 mgF/ L |
| Q80TG1 | KAT8 regulatory NSL complex subunit 1 | 60 | 50 mgF/ L |
| Q8C0N1 | Kinesin-like protein KIF2B | 61 | 50 mgF/ L |
| Q99PH1 | Leucine-rich repeat-containing protein 4 | 57 | 50 mgF/ L |
| P0C192 | Leucine-rich repeat-containing protein 4B | 64 | 50 mgF/ L |
| Q922Q8 | Leucine-rich repeat-containing protein 59 | 124 | 50 mgF/ L |
| C8YR32 | Lipoxygenase homology domain-containing protein 1 | 74 | 50 mgF/ L |
| P16125 | L-lactate dehydrogenase B chain | 270 | 50 mgF/ L |
| P00342 | L-lactate dehydrogenase C chain | 223 | 50 mgF/ L |
| P41245 | Matrix metalloproteinase-9 | 53 | 50 mgF/ L |
| Q9CXI5 | Mesencephalic astrocyte-derived neurotrophic factor | 706 | 50 mgF/ L |
| Q8C7H1 | Methylmalonic aciduria type A homolog, mitochondrial | 125 | 50 mgF/ L |
| P48377 | MHC class II regulatory factor RFX1 | 63 | 50 mgF/ L |
| Q7TT79 | Microcephalin | 84 | 50 mgF/ L |
| Q80Y86 | Mitogen-activated protein kinase 15 | 92 | 50 mgF/ L |
| Q6P5G0 | Mitogen-activated protein kinase 4 | 126 | 50 mgF/ L |
| Q9WTX8 | Mitotic spindle assembly checkpoint protein MAD1 | 73 | 50 mgF/ L |
| F7BJB9 | MORC family CW-type zinc finger protein 3 | 56 | 50 mgF/ L |
| A6H6E2 | Multimerin-2 | 87 | 50 mgF/ L |
| Q8K5B2 | Multiple coagulation factor deficiency protein 2 homolog | 1254 | 50 mgF/ L |
| Q8VBX6 | Multiple PDZ domain protein | 92 | 50 mgF/ L |
| Q6KAU4 | Multivesicular body subunit 12B | 182 | 50 mgF/ L |
| Q6NZR2 | Myb/SANT-like DNA-binding domain-containing protein 2 | 67 | 50 mgF/ L |
| P11247 | Myeloperoxidase | 150 | 50 mgF/ L |
| P09541 | Myosin light chain 4 | 132 | 50 mgF/ L |
| Q6URW6 | Myosin-14 | 56 | 50 mgF/ L |
| Q9DCS9 | NADH dehydrogenase [ubiquinone] 1 beta subcomplex subunit 10 | 117 | 50 mgF/ L |
| Q8CH77 | Neuron navigator 1 | 61 | 50 mgF/ L |
| P10493 | Nidogen-1 | 60 | 50 mgF/ L |
| P12813 | Nuclear receptor subfamily 4 group A member 1 | 48 | 50 mgF/ L |
| Q02819 | Nucleobindin-1 | 23 | 50 mgF/ L |
| Q9JIK5 | Nucleolar RNA helicase 2 | 62 | 50 mgF/ L |
| Q06348 | Paired mesoderm homeobox protein 2 | 96 | 50 mgF/ L |
| Q3ULF4 | Paraplegin | 238 | 50 mgF/ L |
| Q8CEE6 | PAS domain-containing serine/threonine-protein kinase | 52 | 50 mgF/ L |
| P45878 | Peptidyl-prolyl cis-trans isomerase FKBP2 | 449 | 50 mgF/ L |
| Q62009 | Periostin | 235 | 50 mgF/ L |
| Q7TNF8 | Peripheral-type benzodiazepine receptor-associated protein 1 | 37 | 50 mgF/ L |
| Q8CBQ5 | Phosphatidylinositol 4-kinase type 2-beta | 281 | 50 mgF/ L |
| Q8BH04 | Phosphoenolpyruvate carboxykinase [GTP], mitochondrial | 90 | 50 mgF/ L |
| Q8K212 | Phosphofurin acidic cluster sorting protein 1 | 58 | 50 mgF/ L |
| Q9Z2M7 | Phosphomannomutase 2 | 113 | 50 mgF/ L |
| Q68FH0 | Plakophilin-4 | 64 | 50 mgF/ L |
| Q6Q477 | Plasma membrane calcium-transporting ATPase 4 | 46 | 50 mgF/ L |
| Q6PDH0 | Pleckstrin homology-like domain family B member 1 | 328 | 50 mgF/ L |
| Q8K1N2 | Pleckstrin homology-like domain family B member 2 | 75 | 50 mgF/ L |
| Q9WVJ0 | Potassium voltage-gated channel subfamily H member 3 | 63 | 50 mgF/ L |
| Q8K4P0 | pre-mRNA 3' end processing protein WDR33 | 142 | 50 mgF/ L |
| Q8K301 | Probable ATP-dependent RNA helicase DDX52 | 63 | 50 mgF/ L |
| Q99PJ2 | Probable E3 ubiquitin-protein ligase TRIM8 | 72 | 50 mgF/ L |
| Q91ZA3 | Propionyl-CoA carboxylase alpha chain, mitochondrial | 58 | 50 mgF/ L |
| P99026 | Proteasome subunit beta type-4 | 86 | 50 mgF/ L |
| Q9DB52 | Protein FAM122A | 70 | 50 mgF/ L |
| Q5SXA9 | Protein KIBRA | 49 | 50 mgF/ L |
| Q8CGC4 | Protein LSM14 homolog B | 111 | 50 mgF/ L |
| Q80WJ7 | Protein LYRIC | 84 | 50 mgF/ L |
| A4Q9F1 | Protein monoglycylase TTLL8 | 136 | 50 mgF/ L |
| Q91XT4 | Protein transport protein Sec16B | 81 | 50 mgF/ L |
| Q8K3V4 | Protein-arginine deiminase type-6 | 63 | 50 mgF/ L |
| P19221 | Prothrombin | 137 | 50 mgF/ L |
| Q8R1F5 | Putative hydroxypyruvate isomerase | 104 | 50 mgF/ L |
| Q8K183 | Pyridoxal kinase | 172 | 50 mgF/ L |
| Q8CI78 | Required for meiotic nuclear division protein 1 homolog | 64 | 50 mgF/ L |
| P28704 | Retinoic acid receptor RXR-beta | 134 | 50 mgF/ L |
| Q8VCH7 | Retinol dehydrogenase 10 | 83 | 50 mgF/ L |
| Q91YM2 | Rho GTPase-activating protein 35 | 106 | 50 mgF/ L |
| Q5FWH6 | Rho guanine nucleotide exchange factor 15 | 66 | 50 mgF/ L |
| Q8BWA8 | Rho guanine nucleotide exchange factor 19 | 220 | 50 mgF/ L |
| Q9CTN4 | Rho-related BTB domain-containing protein 3 | 122 | 50 mgF/ L |
| Q9Z1M4 | Ribosomal protein S6 kinase beta-2 | 553 | 50 mgF/ L |
| Q9D7H3 | RNA 3'-terminal phosphate cyclase | 184 | 50 mgF/ L |
| P56959 | RNA-binding protein FUS | 226 | 50 mgF/ L |
| A7XUY5 | Selection and upkeep of intraepithelial T-cells protein 5 | 69 | 50 mgF/ L |
| O88632 | Semaphorin-3F | 142 | 50 mgF/ L |
| Q62179 | Semaphorin-4B | 94 | 50 mgF/ L |
| O09126 | Semaphorin-4D | 64 | 50 mgF/ L |
| P42208 | Septin-2 | 281 | 50 mgF/ L |
| Q8BHI9 | Serine/threonine-protein kinase NIM1 | 151 | 50 mgF/ L |
| Q922R5 | Serine/threonine-protein phosphatase 4 regulatory subunit 3B | 89 | 50 mgF/ L |
| Q6S5L9 | SHC-transforming protein 4 | 72 | 50 mgF/ L |
| Q91V61 | Sideroflexin-3 | 83 | 50 mgF/ L |
| Q99LM3 | Smoothelin-like protein 1 | 112 | 50 mgF/ L |
| Q91XA5 | snRNA-activating protein complex subunit 2 | 100 | 50 mgF/ L |
| Q80SU6 | Sodium-dependent phosphate transport protein 2C | 78 | 50 mgF/ L |
| P06880 | Somatotropin | 125 | 50 mgF/ L |
| Q7TME2 | Sperm-associated antigen 5 | 53 | 50 mgF/ L |
| Q9D5R4 | Spermatogenesis-associated protein 1 | 75 | 50 mgF/ L |
| P52019 | Squalene monooxygenase | 130 | 50 mgF/ L |
| Q80TF6 | StAR-related lipid transfer protein 9 | 81 | 50 mgF/ L |
| Q9D0K2 | Succinyl-CoA:3-ketoacid coenzyme A transferase 1, mitochondrial | 61 | 50 mgF/ L |
| Q62465 | Synaptic vesicle membrane protein VAT-1 homolog | 109 | 50 mgF/ L |
| Q8R570 | Synaptosomal-associated protein 47 | 51 | 50 mgF/ L |
| Q8R2K4 | TAF6-like RNA polymerase II p300/CBP-associated factor-associated factor 65 kDa subunit 6L | 55 | 50 mgF/ L |
| Q921F2 | TAR DNA-binding protein 43 | 355 | 50 mgF/ L |
| B9EKI3 | TATA element modulatory factor | 69 | 50 mgF/ L |
| P80316 | T-complex protein 1 subunit epsilon | 83 | 50 mgF/ L |
| Q8BTG3 | T-complex protein 11-like protein 1 | 102 | 50 mgF/ L |
| Q93092 | Transaldolase | 255 | 50 mgF/ L |
| Q3TKT4 | Transcription activator BRG1 | 40 | 50 mgF/ L |
| Q60722 | Transcription factor 4 | 113 | 50 mgF/ L |
| Q704Y3 | Transient receptor potential cation channel subfamily V member 1 | 72 | 50 mgF/ L |
| Q9D2R4 | Transmembrane emp24 domain-containing protein 11 | 46 | 50 mgF/ L |
| Q62393 | Tumor protein D52 | 357 | 50 mgF/ L |
| Q62120 | Tyrosine-protein kinase JAK2 | 45 | 50 mgF/ L |
| Q5DU02 | Ubiquitin carboxyl-terminal hydrolase 22 | 87 | 50 mgF/ L |
| Q8BWR4 | Ubiquitin carboxyl-terminal hydrolase 40 | 100 | 50 mgF/ L |
| Q9ES00 | Ubiquitin conjugation factor E4 B | 101 | 50 mgF/ L |
| Q8BGG7 | Ubiquitin-associated and SH3 domain-containing protein B | 68 | 50 mgF/ L |
| Q8R3I9 | UDP-GlcNAc:betaGal beta-1,3-N-acetylglucosaminyltransferase 8 | 250 | 50 mgF/ L |
| O88329 | Unconventional myosin-Ia | 66 | 50 mgF/ L |
| P46735 | Unconventional myosin-Ib | 79 | 50 mgF/ L |
| Q9Z1Q9 | Valine--tRNA ligase | 117 | 50 mgF/ L |
| Q60930 | Voltage-dependent anion-selective channel protein 2 | 152 | 50 mgF/ L |
| Q8CC27 | Voltage-dependent L-type calcium channel subunit beta-2 | 105 | 50 mgF/ L |
| Q9Z1G4 | V-type proton ATPase 116 kDa subunit a isoform 1 | 65 | 50 mgF/ L |
| O88342 | WD repeat-containing protein 1 | 73 | 50 mgF/ L |
| Q8BND3 | WD repeat-containing protein 35 | 46 | 50 mgF/ L |
| E9PYY5 | WD repeat-containing protein 78 | 55 | 50 mgF/ L |
| Q8K088 | Zinc finger and BTB domain-containing protein 6 | 92 | 50 mgF/ L |
| Q571J5 | Zinc finger protein 354C | 78 | 50 mgF/ L |
| Q6NV66 | Zinc finger protein 646 | 87 | 50 mgF/ L |
| Q75N73 | Zinc transporter ZIP14 | 188 | 50 mgF/ L |
| A2AP18 | 1-phosphatidylinositol 4,5-bisphosphate phosphodiesterase eta-2 | 100 | control |
| Q9CQ62 | 2,4-dienoyl-CoA reductase, mitochondrial | 155 | control |
| Q9Z0S1 | 3'(2'),5'-bisphosphate nucleotidase 1 | 142 | control |
| O08756 | 3-hydroxyacyl-CoA dehydrogenase type-2 | 114 | control |
| Q8BWT1 | 3-ketoacyl-CoA thiolase, mitochondrial | 204 | control |
| P63325 | 40S ribosomal protein S10 | 319 | control |
| P63323 | 40S ribosomal protein S12 | 257 | control |
| P62264 | 40S ribosomal protein S14 | 693 | control |
| P63276 | 40S ribosomal protein S17 | 389 | control |
| P60867 | 40S ribosomal protein S20 | 260 | control |
| Q6ZWU9 | 40S ribosomal protein S27 | 707 | control |
| Q6ZWY3 | 40S ribosomal protein S27-like | 707 | control |
| Q6ZWV3 | 60S ribosomal protein L10 | 224 | control |
| P86048 | 60S ribosomal protein L10-like | 224 | control |
| Q9CR57 | 60S ribosomal protein L14 | 469 | control |
| P67984 | 60S ribosomal protein L22 | 220 | control |
| P62900 | 60S ribosomal protein L31 | 338 | control |
| Q9D8M4 | 60S ribosomal protein L7-like 1 | 72 | control |
| Q7TT18 | Activating transcription factor 7-interacting protein 1 | 231 | control |
| Q80XL6 | Acyl-CoA dehydrogenase family member 11 | 78 | control |
| Q8BQS5 | Adiponectin receptor protein 2 | 157 | control |
| Q8BMI3 | ADP-ribosylation factor-binding protein GGA3 | 65 | control |
| Q9QZQ1 | Afadin | 123 | control |
| Q9JHW9 | Aldehyde dehydrogenase family 1 member A3 | 172 | control |
| Q9CZS1 | Aldehyde dehydrogenase X, mitochondrial | 86 | control |
| P46660 | Alpha-internexin | 87 | control |
| Q9QXJ1 | Amyloid-beta A4 precursor protein-binding family B member 1 | 234 | control |
| D3Z3C6 | AN1-type zinc finger protein 4 | 83 | control |
| G3UZ78 | Androglobin | 112 | control |
| Q80V94 | AP-4 complex subunit epsilon-1 | 132 | control |
| P09813 | Apolipoprotein A-II | 1176 | control |
| Q9Z0X1 | Apoptosis-inducing factor 1, mitochondrial | 77 | control |
| Q9WTY4 | Aquaporin-5 | 254 | control |
| Q8BVF9 | Archaemetzincin-1 | 70 | control |
| Q6HA09 | Astacin-like metalloendopeptidase | 70 | control |
| Q91YH5 | Atlastin-3 | 175 | control |
| Q91VR2 | ATP synthase subunit gamma, mitochondrial | 248 | control |
| Q91V24 | ATP-binding cassette sub-family A member 7 | 73 | control |
| Q9Z2H5 | Band 4.1-like protein 1 | 76 | control |
| Q8BHT6 | Beta-1,3-glucosyltransferase | 83 | control |
| Q99L88 | Beta-1-syntrophin | 92 | control |
| Q8CGC7 | Bifunctional glutamate/proline--tRNA ligase | 182 | control |
| P28653 | Biglycan | 84 | control |
| Q8K3W0 | BRISC and BRCA1-A complex member 2 | 96 | control |
| A2AHJ4 | Bromodomain and WD repeat-containing protein 3 | 256 | control |
| B2RQC6 | CAD protein | 102 | control |
| Q80XR2 | Calcium-transporting ATPase type 2C member 1 | 92 | control |
| P35564 | Calnexin | 162 | control |
| O35350 | Calpain-1 catalytic subunit | 87 | control |
| Q9ESK3 | Calpain-10 | 125 | control |
| P12367 | cAMP-dependent protein kinase type II-alpha regulatory subunit | 125 | control |
| P13634 | Carbonic anhydrase 1 | 132 | control |
| Q8CB62 | Centrobin | 131 | control |
| Q5SW19 | Clustered mitochondria protein homolog | 63 | control |
| Q9QZE5 | Coatomer subunit gamma-1 | 105 | control |
| Q9QXK3 | Coatomer subunit gamma-2 | 122 | control |
| Q9DA73 | Coiled-coil domain-containing protein 89 | 69 | control |
| Q5SU73 | Coilin | 133 | control |
| Q9Z160 | Conserved oligomeric Golgi complex subunit 1 | 165 | control |
| P07310 | Creatine kinase M-type | 328 | control |
| P11440 | Cyclin-dependent kinase 1 | 245 | control |
| Q9JM84 | Cystatin 10 | 482 | control |
| Q03401 | Cysteine-rich secretory protein 1 | 295 | control |
| Q61753 | D-3-phosphoglycerate dehydrogenase | 81 | control |
| Q8CIQ7 | Dedicator of cytokinesis protein 3 | 123 | control |
| Q3U1T9 | DENN domain-containing protein 1B | 75 | control |
| Q8C4S8 | DENN domain-containing protein 2A | 109 | control |
| P31001 | Desmin | 78 | control |
| Q7M6Y5 | Deuterosome assembly protein 1 | 113 | control |
| O08749 | Dihydrolipoyl dehydrogenase, mitochondrial | 89 | control |
| Q8BMF4 | Dihydrolipoyllysine-residue acetyltransferase component of pyruvate dehydrogenase complex, mitochondrial | 79 | control |
| P31428 | Dipeptidase 1 | 302 | control |
| Q64511 | DNA topoisomerase 2-beta | 104 | control |
| Q6ZQF0 | DNA topoisomerase 2-binding protein 1 | 290 | control |
| Q60611 | DNA-binding protein SATB1 | 107 | control |
| Q9D7M8 | DNA-directed RNA polymerase II subunit RPB4 | 214 | control |
| Q91YW3 | DnaJ homolog subfamily C member 3 | 115 | control |
| Q6XUX1 | Dual serine/threonine and tyrosine protein kinase | 126 | control |
| Q8BZ98 | Dynamin-3 | 134 | control |
| Q8BW94 | Dynein heavy chain 3, axonemal | 99 | control |
| Q8BM54 | E3 ubiquitin-protein ligase MYLIP | 78 | control |
| Q9WTV7 | E3 ubiquitin-protein ligase RLIM | 75 | control |
| Q7TNG5 | Echinoderm microtubule-associated protein-like 2 | 88 | control |
| Q8K0L2 | Ectonucleoside triphosphate diphosphohydrolase 8 | 285 | control |
| Q60902 | Epidermal growth factor receptor substrate 15-like 1 | 92 | control |
| Q99JW5 | Epithelial cell adhesion molecule | 250 | control |
| Q6GQV7 | Erythroid differentiation-related factor 1 | 251 | control |
| Q91V76 | Ester hydrolase C11orf54 homolog | 86 | control |
| Q6ZWX6 | Eukaryotic translation initiation factor 2 subunit 1 | 101 | control |
| Q8R1B4 | Eukaryotic translation initiation factor 3 subunit C | 66 | control |
| Q9WUK2 | Eukaryotic translation initiation factor 4H | 170 | control |
| O35250 | Exocyst complex component 7 | 132 | control |
| Q6P5F9 | Exportin-1 | 231 | control |
| Q00558 | Factor VIII intron 22 protein | 128 | control |
| Q8R123 | FAD synthase | 94 | control |
| Q3TQB2 | FAD-dependent oxidoreductase domain-containing protein 1 | 90 | control |
| Q3TDN2 | FAS-associated factor 2 | 110 | control |
| P19096 | Fatty acid synthase | 66 | control |
| P04117 | Fatty acid-binding protein, adipocyte | 143 | control |
| Q9CQ24 | F-box only protein 36 | 201 | control |
| A2AKB4 | FERM and PDZ domain-containing protein 1 | 118 | control |
| Q8BIE6 | FERM domain-containing protein 4A | 95 | control |
| E9PV24 | Fibrinogen alpha chain | 77 | control |
| Q8VCM7 | Fibrinogen gamma chain | 109 | control |
| O70497 | Ficolin-2 | 105 | control |
| Q9R216 | Frizzled-9 | 78 | control |
| Q3U2I3 | FTS and Hook-interacting protein | 99 | control |
| Q8VE33 | Ganglioside-induced differentiation-associated protein 1-like 1 | 201 | control |
| Q5SNZ0 | Girdin | 98 | control |
| P03995 | Glial fibrillary acidic protein | 55 | control |
| Q91X44 | Glucokinase regulatory protein | 87 | control |
| P26443 | Glutamate dehydrogenase 1, mitochondrial | 115 | control |
| Q01098 | Glutamate receptor ionotropic, NMDA 2C | 97 | control |
| P15105 | Glutamine synthetase | 146 | control |
| O35660 | Glutathione S-transferase Mu 6 | 198 | control |
| Q64521 | Glycerol-3-phosphate dehydrogenase, mitochondrial | 114 | control |
| Q9JJI6 | GPI ethanolamine phosphate transferase 3 | 100 | control |
| P62827 | GTP-binding nuclear protein Ran | 160 | control |
| Q8VEL9 | GTP-binding protein REM 2 | 195 | control |
| P36916 | Guanine nucleotide-binding protein-like 1 | 103 | control |
| Q6TL19 | Guanylate cyclase 2G | 81 | control |
| Q8K0U4 | Heat shock 70 kDa protein 12A | 161 | control |
| Q61699 | Heat shock protein 105 kDa | 143 | control |
| Q99020 | Heterogeneous nuclear ribonucleoprotein A/B | 412 | control |
| P49312 | Heterogeneous nuclear ribonucleoprotein A1 | 414 | control |
| Q9Z130 | Heterogeneous nuclear ribonucleoprotein D-like | 119 | control |
| Q9D0E1 | Heterogeneous nuclear ribonucleoprotein M | 391 | control |
| O88569 | Heterogeneous nuclear ribonucleoproteins A2/B1 | 885 | control |
| P10922 | Histone H1.0 | 391 | control |
| Q9WVG6 | Histone-arginine methyltransferase CARM1 | 276 | control |
| P55200 | Histone-lysine N-methyltransferase 2A | 109 | control |
| P09026 | Homeobox protein Hox-B3 | 145 | control |
| Q9ESM3 | Hyaluronan and proteoglycan link protein 2 | 141 | control |
| Q61425 | Hydroxyacyl-coenzyme A dehydrogenase, mitochondrial | 407 | control |
| Q9DBZ1 | Inhibitor of nuclear factor kappa-B kinase-interacting protein | 98 | control |
| Q8CIM8 | Integrator complex subunit 4 | 181 | control |
| Q9QXH4 | Integrin alpha-X | 127 | control |
| Q9Z0R4 | Intersectin-1 | 78 | control |
| Q5DU25 | IQ motif and SEC7 domain-containing protein 2 | 70 | control |
| Q9DBJ6 | Josephin-1 | 128 | control |
| Q61765 | Keratin, type I cuticular Ha1 | 80 | control |
| Q61897 | Keratin, type I cuticular Ha3-II | 60 | control |
| Q9Z2K1 | Keratin, type I cytoskeletal 16 | 125 | control |
| Q6IMF0 | Keratin, type II cuticular 87 | 55 | control |
| Q9ERE2 | Keratin, type II cuticular Hb1 | 55 | control |
| Q9Z2T6 | Keratin, type II cuticular Hb5 | 55 | control |
| P97861 | Keratin, type II cuticular Hb6 | 55 | control |
| P33173 | Kinesin-like protein KIF1A | 117 | control |
| Q7TNC6 | Kinesin-like protein KIF26B | 66 | control |
| P02468 | Laminin subunit gamma-1 | 169 | control |
| O89112 | LanC-like protein 1 | 162 | control |
| Q8R4U7 | Leucine zipper protein 1 | 181 | control |
| W8DXL4 | Leucine-rich repeat, immunoglobulin-like domain and transmembrane domain-containing protein 3 | 75 | control |
| P51174 | Long-chain specific acyl-CoA dehydrogenase, mitochondrial | 211 | control |
| P59997 | Lysine-specific demethylase 2A | 28 | control |
| P41230 | Lysine-specific demethylase 5C | 65 | control |
| Q8BTZ7 | Mannose-1-phosphate guanyltransferase beta | 164 | control |
| O70423 | Membrane primary amine oxidase | 152 | control |
| O35954 | Membrane-associated phosphatidylinositol transfer protein 1 | 85 | control |
| O55022 | Membrane-associated progesterone receptor component 1 | 155 | control |
| Q8CAQ8 | MICOS complex subunit Mic60 | 88 | control |
| Q9D071 | MMS19 nucleotide excision repair protein homolog | 138 | control |
| P28666 | Murinoglobulin-2 | 66 | control |
| Q5SUV2 | MYCBP-associated protein | 92 | control |
| P97457 | Myosin regulatory light chain 2, skeletal muscle isoform | 1320 | control |
| Q5SX40 | Myosin-1 | 334 | control |
| P13541 | Myosin-3 | 223 | control |
| Q02566 | Myosin-6 | 190 | control |
| Q91Z83 | Myosin-7 | 184 | control |
| A2AQP0 | Myosin-7B | 138 | control |
| Q9Z2C9 | Myotubularin-related protein 7 | 102 | control |
| Q99LC3 | NADH dehydrogenase [ubiquinone] 1 alpha subcomplex subunit 10, mitochondrial | 1049 | control |
| Q91WD5 | NADH dehydrogenase [ubiquinone] iron-sulfur protein 2, mitochondrial | 75 | control |
| Q9CXZ1 | NADH dehydrogenase [ubiquinone] iron-sulfur protein 4, mitochondrial | 92 | control |
| Q60817 | Nascent polypeptide-associated complex subunit alpha | 129 | control |
| P70670 | Nascent polypeptide-associated complex subunit alpha, muscle-specific form | 150 | control |
| Q6ZQA0 | Neurobeachin-like protein 2 | 84 | control |
| P19246 | Neurofilament heavy polypeptide | 79 | control |
| P08551 | Neurofilament light polypeptide | 75 | control |
| P08553 | Neurofilament medium polypeptide | 62 | control |
| Q62443 | Neuronal pentraxin-1 | 105 | control |
| Q8CAF4 | NHS-like protein 1 | 102 | control |
| P42580 | NK1 transcription factor-related protein 2 | 115 | control |
| Q8C163 | Nuclease EXOG, mitochondrial | 332 | control |
| Q9CZ30 | Obg-like ATPase 1 | 75 | control |
| P32848 | Parvalbumin alpha | 932 | control |
| Q3URU2 | Paternally-expressed gene 3 protein | 194 | control |
| Q9WUA2 | Phenylalanine--tRNA ligase beta subunit | 159 | control |
| Q8VEM8 | Phosphate carrier protein, mitochondrial | 294 | control |
| P97350 | Plakophilin-1 | 201 | control |
| G5E829 | Plasma membrane calcium-transporting ATPase 1 | 65 | control |
| Q9CY58 | Plasminogen activator inhibitor 1 RNA-binding protein | 202 | control |
| Q3UH93 | Plexin-D1 | 100 | control |
| Q6PCL9 | Poly(A) polymerase gamma | 238 | control |
| Q91VA6 | Polymerase delta-interacting protein 2 | 80 | control |
| P48678 | Prelamin-A/C | 82 | control |
| A2AKX3 | Probable helicase senataxin | 30 | control |
| Q9DAN1 | Protein disulfide-isomerase-like protein of the testis | 132 | control |
| Q8CJF7 | Protein ELYS | 125 | control |
| Q501J2 | Protein FAM173A | 181 | control |
| Q8BHZ0 | Protein FAM49A | 130 | control |
| Q8C753 | Protein KIAA0556 | 58 | control |
| O55126 | Protein NipSnap homolog 2 | 160 | control |
| Q8C8N2 | Protein SCAI | 206 | control |
| Q08642 | Protein-arginine deiminase type-2 | 61 | control |
| P23492 | Purine nucleoside phosphorylase | 167 | control |
| P35486 | Pyruvate dehydrogenase E1 component subunit alpha, somatic form, mitochondrial | 59 | control |
| O35551 | Rab GTPase-binding effector protein 1 | 112 | control |
| P61027 | Ras-related protein Rab-10 | 2350 | control |
| P35283 | Ras-related protein Rab-12 | 1028 | control |
| Q9DD03 | Ras-related protein Rab-13 | 1284 | control |
| Q91V41 | Ras-related protein Rab-14 | 1163 | control |
| Q8K386 | Ras-related protein Rab-15 | 2369 | control |
| P62821 | Ras-related protein Rab-1A | 2476 | control |
| Q9D1G1 | Ras-related protein Rab-1B | 2366 | control |
| Q504M8 | Ras-related protein Rab-26 | 999 | control |
| Q923S9 | Ras-related protein Rab-30 | 999 | control |
| O35963 | Ras-related protein Rab-33B | 1715 | control |
| Q6PHN9 | Ras-related protein Rab-35 | 2107 | control |
| Q9JKM7 | Ras-related protein Rab-37 | 999 | control |
| Q8BHD0 | Ras-related protein Rab-39A | 1621 | control |
| Q8BHC1 | Ras-related protein Rab-39B | 999 | control |
| P63011 | Ras-related protein Rab-3A | 999 | control |
| Q9CZT8 | Ras-related protein Rab-3B | 999 | control |
| P62823 | Ras-related protein Rab-3C | 999 | control |
| P35276 | Ras-related protein Rab-3D | 999 | control |
| Q8CG50 | Ras-related protein Rab-43 | 999 | control |
| P56371 | Ras-related protein Rab-4A | 999 | control |
| Q91ZR1 | Ras-related protein Rab-4B | 999 | control |
| P35279 | Ras-related protein Rab-6A | 1606 | control |
| P61294 | Ras-related protein Rab-6B | 1606 | control |
| P55258 | Ras-related protein Rab-8A | 2282 | control |
| P61028 | Ras-related protein Rab-8B | 2282 | control |
| Q09PK2 | Retroviral-like aspartic protease 1 | 104 | control |
| Q91VI7 | Ribonuclease inhibitor | 173 | control |
| Q9CXK9 | RNA-binding protein 33 | 95 | control |
| Q8BHW9 | Schlafen-like protein 1 | 93 | control |
| F2YMG0 | Serine protease 56 | 60 | control |
| P29621 | Serine protease inhibitor A3C | 130 | control |
| Q80X76 | Serine protease inhibitor A3F | 130 | control |
| Q91WP6 | Serine protease inhibitor A3N | 130 | control |
| Q9QZX7 | Serine racemase | 91 | control |
| Q8C0C4 | Serine-rich coiled-coil domain-containing protein 1 | 137 | control |
| P52430 | Serum paraoxonase/arylesterase 1 | 127 | control |
| Q62087 | Serum paraoxonase/lactonase 3 | 214 | control |
| Q8BH49 | Sesquipedalian-1 | 341 | control |
| Q8K2Q9 | Shootin-1 | 91 | control |
| Q9JM90 | Signal-transducing adaptor protein 1 | 233 | control |
| Q91Z67 | SLIT-ROBO Rho GTPase-activating protein 2 | 180 | control |
| Q3UZP4 | Small VCP/p97-interacting protein | 136 | control |
| Q62417 | Sorbin and SH3 domain-containing protein 1 | 55 | control |
| O55183 | Stanniocalcin-1 | 114 | control |
| Q9CPR7 | Suppressor of IKBKE 1 | 328 | control |
| Q3UKC1 | Tax1-binding protein 1 homolog | 122 | control |
| Q9D494 | Telomere repeats-binding bouquet formation protein 2 | 159 | control |
| Q8R3G9 | Tetraspanin-8 | 214 | control |
| Q7TN22 | Thioredoxin domain-containing protein 16 | 94 | control |
| Q91W90 | Thioredoxin domain-containing protein 5 | 487 | control |
| Q3U269 | Thiosulfate sulfurtransferase/rhodanese-like domain-containing protein 2 | 152 | control |
| Q60803 | TNF receptor-associated factor 3 | 70 | control |
| P70399 | TP53-binding protein 1 | 103 | control |
| Q8VE65 | Transcription initiation factor TFIID subunit 12 | 298 | control |
| Q5NC05 | Transcription termination factor 2 | 142 | control |
| Q9JJG0 | Transforming acidic coiled-coil-containing protein 2 | 120 | control |
| Q62186 | Translocon-associated protein subunit delta | 138 | control |
| Q99KF1 | Transmembrane emp24 domain-containing protein 9 | 263 | control |
| Q8VHK8 | Transmembrane protease serine 11D | 128 | control |
| Q8BNV1 | tRNA (uracil-5-)-methyltransferase homolog A | 260 | control |
| Q3UX10 | Tubulin alpha chain-like 3 | 101 | control |
| P83887 | Tubulin gamma-1 chain | 103 | control |
| Q8VCK3 | Tubulin gamma-2 chain | 103 | control |
| P24529 | Tyrosine 3-monooxygenase | 327 | control |
| Q5I043 | Ubiquitin carboxyl-terminal hydrolase 28 | 61 | control |
| P62838 | Ubiquitin-conjugating enzyme E2 D2 | 480 | control |
| P61079 | Ubiquitin-conjugating enzyme E2 D3 | 480 | control |
| Q8VI16 | UDP-GlcNAc:betaGal beta-1,3-N-acetylglucosaminyltransferase 9 | 159 | control |
| Q63886 | UDP-glucuronosyltransferase 1-1 | 248 | control |
| P70691 | UDP-glucuronosyltransferase 1-2 | 248 | control |
| Q64435 | UDP-glucuronosyltransferase 1-6 | 248 | control |
| Q6ZQM8 | UDP-glucuronosyltransferase 1-7C | 248 | control |
| Q62452 | UDP-glucuronosyltransferase 1-9 | 249 | control |
| A1A535 | Ventricular zone-expressed PH domain-containing protein 1 | 81 | control |
| P50544 | Very long-chain specific acyl-CoA dehydrogenase, mitochondrial | 120 | control |
| Q6QD59 | Vesicle transport protein SEC20 | 121 | control |
| P21614 | Vitamin D-binding protein | 109 | control |
| Q6PHS9 | Voltage-dependent calcium channel subunit alpha-2/delta-2 | 58 | control |
| P50516 | V-type proton ATPase catalytic subunit A | 120 | control |
| Q6VNB8 | WD repeat and FYVE domain-containing protein 3 | 106 | control |
| Q91V09 | WD repeat-containing protein 13 | 93 | control |
| Q8R1D1 | Zinc finger protein 426 | 361 | control |
| Q7TSH3 | Zinc finger protein 516 | 56 | control |

^a^Identification is based on proteins ID from UniProt protein database, reviewed only (<http://www.uniprot.org/>).

^b^ Proteins with expression significantly altered are organized according to the ratio

*Indicates unique proteins in alphabetical order.

**Table S3**. Proteins with different expression significantly altered in the submandibular glands of mice exposed to 50 mg F/L vs. 10 mg F/L

| ***^a^*Access Number** | **Protein name description** | **PLGS**  **Score** | **^b^Ratio  50 mgF/ L : 10 mgF/ L** |
| --- | --- | --- | --- |
| B9EJA2 | Cortactin-binding protein 2 | 334 | 79.84 |
| Q61900 | Submaxillary gland androgen-regulated protein 3A | 511 | 3.16 |
| P00687 | Alpha-amylase 1 | 337 | 2.77 |
| P00688 | Pancreatic alpha-amylase | 319 | 2.12 |
| Q9QXK2 | E3 ubiquitin-protein ligase RAD18 | 72 | 2.10 |
| Q91XA9 | Acidic mammalian chitinase | 415 | 1.77 |
| A2CG49 | Kalirin | 86 | 1.55 |
| Q3UNW5 | Transcription factor CP2-like protein 1 | 196 | 1.55 |
| E9Q8I9 | Protein furry homolog | 52 | 1.28 |
| Q3UJB9 | Enhancer of mRNA-decapping protein 4 | 135 | 1.25 |
| Q8C6C9 | Protein LEG1 homolog | 4298 | 1.15 |
| Q61759 | Kallikrein 1-related peptidase b21 | 24307 | 1.09 |
| Q9JM71 | Kallikrein 1-related peptidase b27 | 27693 | 1.07 |
| Q61754 | Kallikrein 1-related peptidase b24 | 11521 | 1.04 |
| P36369 | Kallikrein 1-related peptidase b26 | 31102 | 1.04 |
| P00756 | Kallikrein 1-related peptidase b3 | 15907 | 1.03 |
| P04071 | Kallikrein 1-related peptidase b16 | 22868 | 0.98 |
| P15948 | Kallikrein 1-related peptidase b22 | 52433 | 0.95 |
| P56565 | Protein S100-A1 | 4981 | 0.94 |
| P21550 | Beta-enolase | 3562 | 0.92 |
| Q922R8 | Protein disulfide-isomerase A6 | 1428 | 0.91 |
| P07759 | Serine protease inhibitor A3K | 800 | 0.91 |
| P17183 | Gamma-enolase | 3355 | 0.90 |
| P15946 | Kallikrein 1-related peptidase b11 | 17541 | 0.90 |
| P47955 | 60S acidic ribosomal protein P1 | 5553 | 0.89 |
| Q9JKR6 | Hypoxia up-regulated protein 1 | 1360 | 0.89 |
| P01878 | Ig alpha chain C region | 326 | 0.89 |
| Q78PY7 | Staphylococcal nuclease domain-containing protein 1 | 164 | 0.89 |
| P62082 | 40S ribosomal protein S7 | 1283 | 0.88 |
| P07758 | Alpha-1-antitrypsin 1-1 | 288 | 0.88 |
| P22599 | Alpha-1-antitrypsin 1-2 | 277 | 0.88 |
| Q03402 | Cysteine-rich secretory protein 3 | 347 | 0.88 |
| P20029 | Endoplasmic reticulum chaperone BiP | 2319 | 0.88 |
| P08113 | Endoplasmin | 1782 | 0.88 |
| P15949 | Kallikrein 1-related peptidase b9 | 47839 | 0.88 |
| Q61879 | Myosin-10 | 337 | 0.88 |
| Q61598 | Rab GDP dissociation inhibitor beta | 199 | 0.88 |
| P24549 | Retinal dehydrogenase 1 | 97 | 0.88 |
| Q62261 | Spectrin beta chain, non-erythrocytic 1 | 73 | 0.88 |
| P20108 | Thioredoxin-dependent peroxide reductase, mitochondrial | 370 | 0.88 |
| Q00896 | Alpha-1-antitrypsin 1-3 | 288 | 0.87 |
| P17182 | Alpha-enolase | 5690 | 0.87 |
| P02104 | Hemoglobin subunit epsilon-Y2 | 2343 | 0.87 |
| P18242 | Cathepsin D | 699 | 0.86 |
| P06745 | Glucose-6-phosphate isomerase | 281 | 0.86 |
| Q9JKF1 | Ras GTPase-activating-like protein IQGAP1 | 140 | 0.86 |
| Q99PL5 | Ribosome-binding protein 1 | 91 | 0.86 |
| P35979 | 60S ribosomal protein L12 | 198 | 0.85 |
| Q04857 | Collagen alpha-1(VI) chain | 90 | 0.85 |
| P60843 | Eukaryotic initiation factor 4A-I | 409 | 0.85 |
| P54818 | Galactocerebrosidase | 106 | 0.85 |
| Q64475 | Histone H2B type 1-B | 4807 | 0.85 |
| Q6ZWY9 | Histone H2B type 1-C/E/G | 4807 | 0.85 |
| Q64478 | Histone H2B type 1-H | 4807 | 0.85 |
| P34884 | Macrophage migration inhibitory factor | 1051 | 0.85 |
| Q9DBJ1 | Phosphoglycerate mutase 1 | 518 | 0.85 |
| Q9QXS1 | Plectin | 33 | 0.85 |
| P62908 | 40S ribosomal protein S3 | 1481 | 0.84 |
| P14869 | 60S acidic ribosomal protein P0 | 1537 | 0.84 |
| P99027 | 60S acidic ribosomal protein P2 | 1510 | 0.84 |
| Q08091 | Calponin-1 | 1144 | 0.84 |
| Q9CZU6 | Citrate synthase, mitochondrial | 224 | 0.84 |
| Q9JIF7 | Coatomer subunit beta | 101 | 0.84 |
| P30275 | Creatine kinase U-type, mitochondrial | 1476 | 0.84 |
| P10630 | Eukaryotic initiation factor 4A-II | 463 | 0.84 |
| P02088 | Hemoglobin subunit beta-1 | 58438 | 0.84 |
| P02089 | Hemoglobin subunit beta-2 | 23050 | 0.84 |
| Q8CGP5 | Histone H2A type 1-F | 23604 | 0.84 |
| Q8CGP7 | Histone H2A type 1-K | 23604 | 0.84 |
| Q8CGP2 | Histone H2B type 1-P | 4807 | 0.84 |
| P01867 | Ig gamma-2B chain C region | 552 | 0.84 |
| P09411 | Phosphoglycerate kinase 1 | 1059 | 0.84 |
| Q9D051 | Pyruvate dehydrogenase E1 component subunit beta, mitochondrial | 363 | 0.84 |
| P37804 | Transgelin | 1211 | 0.84 |
| P62858 | 40S ribosomal protein S28 | 886 | 0.84 |
| P48036 | Annexin A5 | 413 | 0.84 |
| P14824 | Annexin A6 | 222 | 0.84 |
| P43024 | Cytochrome c oxidase subunit 6A1, mitochondrial | 1102 | 0.84 |
| Q80W21 | Glutathione S-transferase Mu 7 | 730 | 0.84 |
| Q64523 | Histone H2A type 2-C | 23604 | 0.84 |
| Q8R1M2 | Histone H2A.J | 23604 | 0.84 |
| P62962 | Profilin-1 | 3237 | 0.84 |
| P68040 | Receptor of activated protein C kinase 1 | 198 | 0.84 |
| P45376 | Aldose reductase | 162 | 0.83 |
| Q7TPR4 | Alpha-actinin-1 | 424 | 0.83 |
| P14211 | Calreticulin | 1899 | 0.83 |
| Q02788 | Collagen alpha-2(VI) chain | 69 | 0.83 |
| P97315 | Cysteine and glycine-rich protein 1 | 497 | 0.83 |
| P56391 | Cytochrome c oxidase subunit 6B1 | 1742 | 0.83 |
| Q62188 | Dihydropyrimidinase-related protein 3 | 1359 | 0.83 |
| Q91VC3 | Eukaryotic initiation factor 4A-III | 360 | 0.83 |
| P15626 | Glutathione S-transferase Mu 2 | 614 | 0.83 |
| P15947 | Kallikrein-1 | 39580 | 0.83 |
| Q9EQ20 | Methylmalonate-semialdehyde dehydrogenase [acylating], mitochondrial | 208 | 0.83 |
| O08638 | Myosin-11 | 562 | 0.83 |
| P17742 | Peptidyl-prolyl cis-trans isomerase A | 5040 | 0.83 |
| Q8VDN2 | Sodium/potassium-transporting ATPase subunit alpha-1 | 636 | 0.83 |
| P40142 | Transketolase | 640 | 0.83 |
| P62702 | 40S ribosomal protein S4, X isoform | 753 | 0.82 |
| P14206 | 40S ribosomal protein SA | 2149 | 0.82 |
| Q9Z1P8 | Angiopoietin-related protein 4 | 239 | 0.82 |
| P00920 | Carbonic anhydrase 2 | 335 | 0.82 |
| Q99LC5 | Electron transfer flavoprotein subunit alpha, mitochondrial | 729 | 0.82 |
| P10126 | Elongation factor 1-alpha 1 | 15504 | 0.82 |
| P10649 | Glutathione S-transferase Mu 1 | 736 | 0.82 |
| Q6GSS7 | Histone H2A type 2-A | 23604 | 0.82 |
| Q61171 | Peroxiredoxin-2 | 1153 | 0.82 |
| O08709 | Peroxiredoxin-6 | 1197 | 0.82 |
| P08003 | Protein disulfide-isomerase A4 | 107 | 0.82 |
| Q99PT1 | Rho GDP-dissociation inhibitor 1 | 570 | 0.82 |
| Q01853 | Transitional endoplasmic reticulum ATPase | 743 | 0.82 |
| Q9CXW4 | 60S ribosomal protein L11 | 171 | 0.81 |
| P63268 | Actin, gamma-enteric smooth muscle | 29666 | 0.81 |
| P57780 | Alpha-actinin-4 | 901 | 0.81 |
| P56135 | ATP synthase subunit f, mitochondrial | 249 | 0.81 |
| Q8CIE6 | Coatomer subunit alpha | 67 | 0.81 |
| Q8BTM8 | Filamin-A | 422 | 0.81 |
| P16858 | Glyceraldehyde-3-phosphate dehydrogenase | 7300 | 0.81 |
| Q64467 | Glyceraldehyde-3-phosphate dehydrogenase, testis-specific | 488 | 0.81 |
| P63017 | Heat shock cognate 71 kDa protein | 2575 | 0.81 |
| Q9CQN1 | Heat shock protein 75 kDa, mitochondrial | 2341 | 0.81 |
| P01942 | Hemoglobin subunit alpha | 11813 | 0.81 |
| Q9D154 | Leukocyte elastase inhibitor A | 152 | 0.81 |
| P14152 | Malate dehydrogenase, cytoplasmic | 3123 | 0.81 |
| Q91YT0 | NADH dehydrogenase [ubiquinone] flavoprotein 1, mitochondrial | 128 | 0.81 |
| P29341 | Polyadenylate-binding protein 1 | 616 | 0.81 |
| Q61838 | Pregnancy zone protein | 123 | 0.81 |
| P27773 | Protein disulfide-isomerase A3 | 2831 | 0.81 |
| Q921I1 | Serotransferrin | 1560 | 0.81 |
| Q02053 | Ubiquitin-like modifier-activating enzyme 1 | 173 | 0.81 |
| Q60597 | 2-oxoglutarate dehydrogenase, mitochondrial | 98 | 0.80 |
| P68033 | Actin, alpha cardiac muscle 1 | 31785 | 0.80 |
| P56480 | ATP synthase subunit beta, mitochondrial | 4906 | 0.80 |
| P62897 | Cytochrome c, somatic | 864 | 0.80 |
| Q4VA61 | Down syndrome cell adhesion molecule-like protein 1 homolog | 101 | 0.80 |
| P11499 | Heat shock protein HSP 90-beta | 4350 | 0.80 |
| P01837 | Immunoglobulin kappa constant | 681 | 0.80 |
| Q9CQ19 | Myosin regulatory light polypeptide 9 | 4476 | 0.80 |
| P24369 | Peptidyl-prolyl cis-trans isomerase B | 275 | 0.80 |
| P09103 | Protein disulfide-isomerase | 7579 | 0.80 |
| P52480 | Pyruvate kinase PKM | 3136 | 0.80 |
| Q07417 | Short-chain specific acyl-CoA dehydrogenase, mitochondrial | 220 | 0.80 |
| Q8QZT1 | Acetyl-CoA acetyltransferase, mitochondrial | 287 | 0.79 |
| Q99KI0 | Aconitate hydratase, mitochondrial | 713 | 0.79 |
| Q9DB20 | ATP synthase subunit O, mitochondrial | 1734 | 0.79 |
| P18760 | Cofilin-1 | 680 | 0.79 |
| Q9CR68 | Cytochrome b-c1 complex subunit Rieske, mitochondrial | 533 | 0.79 |
| P07901 | Heat shock protein HSP 90-alpha | 2959 | 0.79 |
| P62806 | Histone H4 | 10900 | 0.79 |
| P08249 | Malate dehydrogenase, mitochondrial | 3401 | 0.79 |
| Q60605 | Myosin light polypeptide 6 | 2726 | 0.79 |
| P99029 | Peroxiredoxin-5, mitochondrial | 1356 | 0.79 |
| P0CG49 | Polyubiquitin-B | 5660 | 0.79 |
| P0CG50 | Polyubiquitin-C | 5660 | 0.79 |
| Q61207 | Prosaposin | 295 | 0.79 |
| Q99J77 | Sialic acid synthase | 584 | 0.79 |
| Q9D1D4 | Transmembrane emp24 domain-containing protein 10 | 181 | 0.79 |
| Q9JJZ2 | Tubulin alpha-8 chain | 789 | 0.79 |
| P62983 | Ubiquitin-40S ribosomal protein S27a | 5660 | 0.79 |
| P62984 | Ubiquitin-60S ribosomal protein L40 | 5660 | 0.79 |
| O35945 | Aldehyde dehydrogenase, cytosolic 1 | 97 | 0.79 |
| Q00897 | Alpha-1-antitrypsin 1-4 | 207 | 0.79 |
| Q68FD5 | Clathrin heavy chain 1 | 123 | 0.79 |
| P00405 | Cytochrome c oxidase subunit 2 | 569 | 0.79 |
| Q9DCW4 | Electron transfer flavoprotein subunit beta | 1212 | 0.79 |
| P26040 | Ezrin | 48 | 0.79 |
| P05064 | Fructose-bisphosphate aldolase A | 3753 | 0.79 |
| P55012 | Solute carrier family 12 member 2 | 138 | 0.79 |
| P17751 | Triosephosphate isomerase | 6419 | 0.79 |
| P63101 | 14-3-3 protein zeta/delta | 3553 | 0.78 |
| Q9DCX2 | ATP synthase subunit d, mitochondrial | 1126 | 0.78 |
| Q9CZ13 | Cytochrome b-c1 complex subunit 1, mitochondrial | 386 | 0.78 |
| P28654 | Decorin | 133 | 0.78 |
| P70696 | Histone H2B type 1-A | 1007 | 0.78 |
| Q9D3R6 | Katanin p60 ATPase-containing subunit A-like 2 | 82 | 0.78 |
| A6H6E2 | Multimerin-2 | 94 | 0.78 |
| Q6PIE5 | Sodium/potassium-transporting ATPase subunit alpha-2 | 364 | 0.78 |
| Q9WVA4 | Transgelin-2 | 247 | 0.78 |
| Q8BMS1 | Trifunctional enzyme subunit alpha, mitochondrial | 281 | 0.78 |
| P63260 | Actin, cytoplasmic 2 | 41048 | 0.77 |
| O88990 | Alpha-actinin-3 | 165 | 0.77 |
| O35855 | Branched-chain-amino-acid aminotransferase, mitochondrial | 279 | 0.77 |
| Q8BGY2 | Eukaryotic translation initiation factor 5A-2 | 374 | 0.77 |
| Q9DCV7 | Keratin, type II cytoskeletal 7 | 2769 | 0.77 |
| P81117 | Nucleobindin-2 | 633 | 0.77 |
| Q61937 | Nucleophosmin | 217 | 0.77 |
| P15532 | Nucleoside diphosphate kinase A | 1968 | 0.77 |
| Q01768 | Nucleoside diphosphate kinase B | 2583 | 0.77 |
| P00796 | Renin-2 | 11524 | 0.77 |
| P09671 | Superoxide dismutase [Mn], mitochondrial | 206 | 0.77 |
| P05213 | Tubulin alpha-1B chain | 1505 | 0.77 |
| Q9D6F9 | Tubulin beta-4A chain | 747 | 0.77 |
| Q922F4 | Tubulin beta-6 chain | 3476 | 0.77 |
| Q8VDJ3 | Vigilin | 167 | 0.77 |
| P60710 | Actin, cytoplasmic 1 | 41048 | 0.76 |
| Q03265 | ATP synthase subunit alpha, mitochondrial | 4522 | 0.76 |
| P12787 | Cytochrome c oxidase subunit 5A, mitochondrial | 965 | 0.76 |
| P10853 | Histone H2B type 1-F/J/L | 4807 | 0.76 |
| P10854 | Histone H2B type 1-M | 4807 | 0.76 |
| Q64524 | Histone H2B type 2-E | 3654 | 0.76 |
| Q9D2U9 | Histone H2B type 3-A | 3654 | 0.76 |
| Q8CGP0 | Histone H2B type 3-B | 3654 | 0.76 |
| Q60854 | Serpin B6 | 6178 | 0.76 |
| P68369 | Tubulin alpha-1A chain | 1586 | 0.76 |
| P68373 | Tubulin alpha-1C chain | 1610 | 0.76 |
| P05214 | Tubulin alpha-3 chain | 1332 | 0.76 |
| P68368 | Tubulin alpha-4A chain | 1988 | 0.76 |
| A2AQ07 | Tubulin beta-1 chain | 224 | 0.76 |
| Q9ERD7 | Tubulin beta-3 chain | 3842 | 0.76 |
| Q9CQV8 | 14-3-3 protein beta/alpha | 1461 | 0.76 |
| P62259 | 14-3-3 protein epsilon | 1939 | 0.76 |
| P40124 | Adenylyl cyclase-associated protein 1 | 339 | 0.76 |
| P61750 | ADP-ribosylation factor 4 | 1602 | 0.76 |
| P84084 | ADP-ribosylation factor 5 | 1113 | 0.76 |
| G5E8K5 | Ankyrin-3 | 93 | 0.76 |
| P63242 | Eukaryotic translation initiation factor 5A-1 | 340 | 0.76 |
| P97807 | Fumarate hydratase, mitochondrial | 339 | 0.76 |
| Q8CGP1 | Histone H2B type 1-K | 4807 | 0.76 |
| Q64525 | Histone H2B type 2-B | 4807 | 0.76 |
| P05784 | Keratin, type I cytoskeletal 18 | 4167 | 0.76 |
| P35700 | Peroxiredoxin-1 | 3681 | 0.76 |
| Q9CWF2 | Tubulin beta-2B chain | 3915 | 0.76 |
| P68372 | Tubulin beta-4B chain | 4077 | 0.76 |
| O70456 | 14-3-3 protein sigma | 1415 | 0.75 |
| P68134 | Actin, alpha skeletal muscle | 31396 | 0.75 |
| Q8VCW8 | Acyl-CoA synthetase family member 2, mitochondrial | 165 | 0.75 |
| Q8BSL7 | ADP-ribosylation factor 2 | 1474 | 0.75 |
| P01027 | Complement C3 | 89 | 0.75 |
| Q8K0G5 | EARP-interacting protein | 103 | 0.75 |
| Q80X90 | Filamin-B | 92 | 0.75 |
| Q8CGP6 | Histone H2A type 1-H | 23604 | 0.75 |
| Q8BFU2 | Histone H2A type 3 | 23604 | 0.75 |
| P15945 | Kallikrein 1-related peptidase b5 | 26505 | 0.75 |
| Q8K5B2 | Multiple coagulation factor deficiency protein 2 homolog | 1254 | 0.75 |
| Q99LX0 | Protein/nucleic acid deglycase DJ-1 | 1337 | 0.75 |
| Q7TMM9 | Tubulin beta-2A chain | 3915 | 0.75 |
| P99024 | Tubulin beta-5 chain | 3987 | 0.75 |
| P68510 | 14-3-3 protein eta | 1335 | 0.74 |
| P61982 | 14-3-3 protein gamma | 1388 | 0.74 |
| P62242 | 40S ribosomal protein S8 | 149 | 0.74 |
| P84078 | ADP-ribosylation factor 1 | 1556 | 0.74 |
| P61205 | ADP-ribosylation factor 3 | 1556 | 0.74 |
| P24270 | Catalase | 317 | 0.74 |
| O08553 | Dihydropyrimidinase-related protein 2 | 168 | 0.74 |
| P19001 | Keratin, type I cytoskeletal 19 | 665 | 0.74 |
| Q3THE2 | Myosin regulatory light chain 12B | 4229 | 0.74 |
| O08807 | Peroxiredoxin-4 | 183 | 0.74 |
| P53657 | Pyruvate kinase PKLR | 100 | 0.74 |
| P08228 | Superoxide dismutase [Cu-Zn] | 1765 | 0.74 |
| P21107 | Tropomyosin alpha-3 chain | 1793 | 0.74 |
| P68254 | 14-3-3 protein theta | 1364 | 0.73 |
| Q8BG05 | Heterogeneous nuclear ribonucleoprotein A3 | 64 | 0.73 |
| Q6PIC6 | Sodium/potassium-transporting ATPase subunit alpha-3 | 320 | 0.73 |
| P47738 | Aldehyde dehydrogenase, mitochondrial | 1369 | 0.73 |
| Q8BFZ3 | Beta-actin-like protein 2 | 10404 | 0.73 |
| P36368 | Epidermal growth factor-binding protein type B | 19039 | 0.73 |
| P11679 | Keratin, type II cytoskeletal 8 | 3603 | 0.73 |
| P26039 | Talin-1 | 283 | 0.73 |
| O88329 | Unconventional myosin-Ia | 118 | 0.73 |
| P51881 | ADP/ATP translocase 2 | 1997 | 0.72 |
| P11881 | Inositol 1,4,5-trisphosphate receptor type 1 | 115 | 0.72 |
| Q9QWL7 | Keratin, type I cytoskeletal 17 | 175 | 0.72 |
| P26041 | Moesin | 72 | 0.72 |
| P09041 | Phosphoglycerate kinase 2 | 68 | 0.72 |
| Q69ZK6 | Probable JmjC domain-containing histone demethylation protein 2C | 82 | 0.72 |
| Q3UMC0 | Spermatogenesis-associated protein 5 | 160 | 0.72 |
| P58252 | Elongation factor 2 | 1134 | 0.71 |
| P01756 | Ig heavy chain V region MOPC 104E | 192 | 0.71 |
| P20152 | Vimentin | 769 | 0.71 |
| P27661 | Histone H2AX | 3230 | 0.70 |
| P01868 | Ig gamma-1 chain C region secreted form | 262 | 0.70 |
| P01869 | Ig gamma-1 chain C region, membrane-bound form | 244 | 0.70 |
| P01757 | Ig heavy chain V region J558 | 192 | 0.70 |
| Q61414 | Keratin, type I cytoskeletal 15 | 122 | 0.70 |
| P58771 | Tropomyosin alpha-1 chain | 1813 | 0.70 |
| P62852 | 40S ribosomal protein S25 | 238 | 0.70 |
| Q3THW5 | Histone H2A.V | 4061 | 0.70 |
| P0C0S6 | Histone H2A.Z | 4061 | 0.70 |
| O88844 | Isocitrate dehydrogenase [NADP] cytoplasmic | 148 | 0.70 |
| P54071 | Isocitrate dehydrogenase [NADP], mitochondrial | 408 | 0.70 |
| P06151 | L-lactate dehydrogenase A chain | 1166 | 0.70 |
| Q8VDD5 | Myosin-9 | 517 | 0.70 |
| P14094 | Sodium/potassium-transporting ATPase subunit beta-1 | 592 | 0.70 |
| Q6IFZ9 | Keratin, type II cytoskeletal 74 | 502 | 0.69 |
| Q8BHN3 | Neutral alpha-glucosidase AB | 73 | 0.69 |
| P50396 | Rab GDP dissociation inhibitor alpha | 90 | 0.69 |
| P62631 | Elongation factor 1-alpha 2 | 1760 | 0.68 |
| P57776 | Elongation factor 1-delta | 434 | 0.68 |
| Q8BH95 | Enoyl-CoA hydratase, mitochondrial | 240 | 0.68 |
| Q64522 | Histone H2A type 2-B | 2575 | 0.68 |
| P06330 | Ig heavy chain V region AC38 205.12 | 192 | 0.68 |
| Q93092 | Transaldolase | 293 | 0.68 |
| P58774 | Tropomyosin beta chain | 1794 | 0.68 |
| P47962 | 60S ribosomal protein L5 | 509 | 0.68 |
| Q99NB1 | Acetyl-coenzyme A synthetase 2-like, mitochondrial | 216 | 0.68 |
| Q922U2 | Keratin, type II cytoskeletal 5 | 400 | 0.68 |
| P10493 | Nidogen-1 | 102 | 0.68 |
| Q9D6P8 | Calmodulin-like protein 3 | 130 | 0.67 |
| Q9R0H5 | Keratin, type II cytoskeletal 71 | 476 | 0.67 |
| Q64436 | Potassium-transporting ATPase alpha chain 1 | 154 | 0.67 |
| Q8C8R3 | Ankyrin-2 | 117 | 0.66 |
| P04104 | Keratin, type II cytoskeletal 1 | 198 | 0.66 |
| P50446 | Keratin, type II cytoskeletal 6A | 390 | 0.66 |
| P61979 | Heterogeneous nuclear ribonucleoprotein K | 153 | 0.65 |
| P07628 | Kallikrein 1-related peptidase b8 | 14774 | 0.65 |
| Q3UU35 | Ovostatin homolog | 132 | 0.65 |
| P06281 | Renin-1 | 9096 | 0.65 |
| Q8VEK3 | Heterogeneous nuclear ribonucleoprotein U | 102 | 0.64 |
| Q9Z331 | Keratin, type II cytoskeletal 6B | 390 | 0.64 |
| Q8CI43 | Myosin light chain 6B | 560 | 0.64 |
| Q8R1F5 | Putative hydroxypyruvate isomerase | 195 | 0.64 |
| Q9WV27 | Sodium/potassium-transporting ATPase subunit alpha-4 | 167 | 0.64 |
| Q8R570 | Synaptosomal-associated protein 47 | 119 | 0.64 |
| Q64727 | Vinculin | 167 | 0.64 |
| Q8CHG3 | GRIP and coiled-coil domain-containing protein 2 | 145 | 0.64 |
| P07744 | Keratin, type II cytoskeletal 4 | 177 | 0.64 |
| P38647 | Stress-70 protein, mitochondrial | 152 | 0.64 |
| P07309 | Transthyretin | 578 | 0.64 |
| Q8VED5 | Keratin, type II cytoskeletal 79 | 177 | 0.63 |
| Q9Z1W8 | Potassium-transporting ATPase alpha chain 2 | 167 | 0.63 |
| Q6IME9 | Keratin, type II cytoskeletal 72 | 240 | 0.63 |
| Q6NXH9 | Keratin, type II cytoskeletal 73 | 177 | 0.63 |
| P00755 | Kallikrein 1-related peptidase b1 | 38253 | 0.62 |
| Q6IFZ6 | Keratin, type II cytoskeletal 1b | 207 | 0.62 |
| Q3TTY5 | Keratin, type II cytoskeletal 2 epidermal | 194 | 0.62 |
| Q80Y86 | Mitogen-activated protein kinase 15 | 149 | 0.61 |
| P10107 | Annexin A1 | 141 | 0.60 |
| P17156 | Heat shock-related 70 kDa protein 2 | 705 | 0.60 |
| P25444 | 40S ribosomal protein S2 | 86 | 0.59 |
| P30999 | Catenin delta-1 | 94 | 0.59 |
| P07724 | Serum albumin | 14561 | 0.58 |
| P07743 | BPI fold-containing family A member 2 | 6240 | 0.57 |
| Q9ESN9 | C-Jun-amino-terminal kinase-interacting protein 3 | 117 | 0.57 |
| Q9R0P5 | Destrin | 301 | 0.57 |
| Q99M73 | Keratin, type II cuticular Hb4 | 413 | 0.57 |
| P17879 | Heat shock 70 kDa protein 1B | 652 | 0.55 |
| Q62009 | Periostin | 138 | 0.55 |
| Q9D3D9 | ATP synthase subunit delta, mitochondrial | 133 | 0.53 |
| P48962 | ADP/ATP translocase 1 | 456 | 0.53 |
| Q61696 | Heat shock 70 kDa protein 1A | 652 | 0.53 |
| P68433 | Histone H3.1 | 2391 | 0.53 |
| P84228 | Histone H3.2 | 2786 | 0.53 |
| P84244 | Histone H3.3 | 2391 | 0.53 |
| P02301 | Histone H3.3C | 2391 | 0.53 |
| P16125 | L-lactate dehydrogenase B chain | 355 | 0.53 |
| Q3UV17 | Keratin, type II cytoskeletal 2 oral | 440 | 0.52 |
| F7BJB9 | MORC family CW-type zinc finger protein 3 | 104 | 0.52 |
| P06467 | Hemoglobin subunit zeta | 371 | 0.51 |
| Q9Z2M7 | Phosphomannomutase 2 | 188 | 0.50 |
| Q91WN4 | Kynurenine 3-monooxygenase | 96 | 0.50 |
| P16627 | Heat shock 70 kDa protein 1-like | 652 | 0.49 |
| Q8BGZ7 | Keratin, type II cytoskeletal 75 | 579 | 0.49 |
| Q7TMK9 | Heterogeneous nuclear ribonucleoprotein Q | 303 | 0.47 |
| Q7TME2 | Sperm-associated antigen 5 | 64 | 0.47 |
| P48774 | Glutathione S-transferase Mu 5 | 226 | 0.44 |
| P00342 | L-lactate dehydrogenase C chain | 202 | 0.43 |
| Q3V132 | ADP/ATP translocase 4 | 274 | 0.42 |
| O08810 | 116 kDa U5 small nuclear ribonucleoprotein component | 548 | 0.39 |
| Q5PR68 | Centrosomal protein of 112 kDa | 80 | 0.37 |
| P08730 | Keratin, type I cytoskeletal 13 | 121 | 0.35 |
| B2RX88 | Centrosome and spindle pole associated protein 1 | 464 | 0.33 |
| Q9CPW0 | Contactin-associated protein-like 2 | 116 | 0.22 |
| Q9Z1Q9 | Valine--tRNA ligase | 101 | 0.11 |
| A2AP18 | 1-phosphatidylinositol 4,5-bisphosphate phosphodiesterase eta-2 | 166 | 50 mgF / L* |
| Q9CX56 | 26S proteasome non-ATPase regulatory subunit 8 | 136 | 50 mgF / L |
| Q91ZV4 | 2-acylglycerol O-acyltransferase 1 | 107 | 50 mgF / L |
| O08756 | 3-hydroxyacyl-CoA dehydrogenase type-2 | 294 | 50 mgF / L |
| Q99L13 | 3-hydroxyisobutyrate dehydrogenase, mitochondrial | 147 | 50 mgF / L |
| Q8BWT1 | 3-ketoacyl-CoA thiolase, mitochondrial | 74 | 50 mgF / L |
| Q9CZX8 | 40S ribosomal protein S19 | 690 | 50 mgF / L |
| P60867 | 40S ribosomal protein S20 | 696 | 50 mgF / L |
| Q9CQR2 | 40S ribosomal protein S21 | 929 | 50 mgF / L |
| Q9CR57 | 60S ribosomal protein L14 | 386 | 50 mgF / L |
| P67984 | 60S ribosomal protein L22 | 232 | 50 mgF / L |
| Q9D8E6 | 60S ribosomal protein L4 | 103 | 50 mgF / L |
| Q9CQ60 | 6-phosphogluconolactonase | 300 | 50 mgF / L |
| P04756 | Acetylcholine receptor subunit alpha | 109 | 50 mgF / L |
| Q9Z2N8 | Actin-like protein 6A | 333 | 50 mgF / L |
| Q9QXN3 | Activating signal cointegrator 1 | 242 | 50 mgF / L |
| Q8K348 | Activin receptor type-1C | 234 | 50 mgF / L |
| Q9QYR9 | Acyl-coenzyme A thioesterase 2, mitochondrial | 134 | 50 mgF / L |
| P61211 | ADP-ribosylation factor-like protein 1 | 314 | 50 mgF / L |
| A2ASQ1 | Agrin | 74 | 50 mgF / L |
| Q9JHW9 | Aldehyde dehydrogenase family 1 member A3 | 89 | 50 mgF / L |
| Q9CZS1 | Aldehyde dehydrogenase X, mitochondrial | 155 | 50 mgF / L |
| P46660 | Alpha-internexin | 59 | 50 mgF / L |
| Q9DBR4 | Amyloid-beta A4 precursor protein-binding family B member 2 | 163 | 50 mgF / L |
| P53995 | Anaphase-promoting complex subunit 1 | 169 | 50 mgF / L |
| Q9CZK6 | Ankyrin repeat and SAM domain-containing protein 3 | 57 | 50 mgF / L |
| Q6PD24 | Ankyrin repeat domain-containing protein 13D | 183 | 50 mgF / L |
| O88312 | Anterior gradient protein 2 homolog | 218 | 50 mgF / L |
| Q80WC7 | Arf-GAP domain and FG repeat-containing protein 2 | 120 | 50 mgF / L |
| Q4LDD4 | Arf-GAP with Rho-GAP domain, ANK repeat and PH domain-containing protein 1 | 482 | 50 mgF / L |
| Q61176 | Arginase-1 | 117 | 50 mgF / L |
| Q91YI0 | Argininosuccinate lyase | 184 | 50 mgF / L |
| Q3URY6 | Armadillo repeat-containing protein 2 | 284 | 50 mgF / L |
| Q3UD01 | Ataxin-7-like protein 3B | 186 | 50 mgF / L |
| Q91VR2 | ATP synthase subunit gamma, mitochondrial | 222 | 50 mgF / L |
| Q5SSE9 | ATP-binding cassette sub-family A member 13 | 86 | 50 mgF / L |
| O88566 | Axin-2 | 115 | 50 mgF / L |
| Q99L88 | Beta-1-syntrophin | 104 | 50 mgF / L |
| P20060 | Beta-hexosaminidase subunit beta | 141 | 50 mgF / L |
| Q8CGC7 | Bifunctional glutamate/proline--tRNA ligase | 69 | 50 mgF / L |
| Q8R015 | Biogenesis of lysosome-related organelles complex 1 subunit 5 | 123 | 50 mgF / L |
| A2A5R2 | Brefeldin A-inhibited guanine nucleotide-exchange protein 2 | 181 | 50 mgF / L |
| Q8K3W0 | BRISC and BRCA1-A complex member 2 | 171 | 50 mgF / L |
| B2RQC6 | CAD protein | 159 | 50 mgF / L |
| O88338 | Cadherin-16 | 109 | 50 mgF / L |
| Q8K1N1 | Calcium-independent phospholipase A2-gamma | 74 | 50 mgF / L |
| Q9D805 | Calpain-9 | 137 | 50 mgF / L |
| P48758 | Carbonyl reductase [NADPH] 1 | 116 | 50 mgF / L |
| P23953 | Carboxylesterase 1C | 104 | 50 mgF / L |
| Q8BK63 | Casein kinase I isoform alpha | 106 | 50 mgF / L |
| E9Q355 | Cation channel sperm-associated protein subunit gamma 1 | 68 | 50 mgF / L |
| C6KI89 | Cation channel sperm-associated protein subunit gamma 2 | 76 | 50 mgF / L |
| P27548 | CD40 ligand | 73 | 50 mgF / L |
| P51949 | CDK-activating kinase assembly factor MAT1 | 143 | 50 mgF / L |
| Q8CII2 | Cell division cycle protein 123 homolog | 75 | 50 mgF / L |
| Q9CXS4 | Centromere protein V | 165 | 50 mgF / L |
| Q6IRU7 | Centrosomal protein of 78 kDa | 62 | 50 mgF / L |
| Q60952 | Centrosome-associated protein CEP250 | 128 | 50 mgF / L |
| Q61410 | cGMP-dependent protein kinase 2 | 240 | 50 mgF / L |
| P0CG14 | Chromosome transmission fidelity protein 8 homolog isoform 2 | 247 | 50 mgF / L |
| Q80VN0 | Cilia- and flagella-associated protein 100 | 97 | 50 mgF / L |
| Q5XJY5 | Coatomer subunit delta | 60 | 50 mgF / L |
| Q3ULW6 | Coiled-coil domain-containing protein 33 | 96 | 50 mgF / L |
| P11087 | Collagen alpha-1(I) chain | 59 | 50 mgF / L |
| Q8BLX7 | Collagen alpha-1(XVI) chain | 231 | 50 mgF / L |
| Q01149 | Collagen alpha-2(I) chain | 56 | 50 mgF / L |
| Q8K2Z4 | Condensin complex subunit 1 | 88 | 50 mgF / L |
| Q8C0L8 | Conserved oligomeric Golgi complex subunit 5 | 112 | 50 mgF / L |
| O88543 | COP9 signalosome complex subunit 3 | 123 | 50 mgF / L |
| Q8K2X3 | CST complex subunit STN1 | 397 | 50 mgF / L |
| Q3TCH7 | Cullin-4A | 282 | 50 mgF / L |
| A2A432 | Cullin-4B | 88 | 50 mgF / L |
| Q8BGU5 | Cyclin-Y | 82 | 50 mgF / L |
| Q03401 | Cysteine-rich secretory protein 1 | 104 | 50 mgF / L |
| Q9JHU4 | Cytoplasmic dynein 1 heavy chain 1 | 105 | 50 mgF / L |
| Q45VK7 | Cytoplasmic dynein 2 heavy chain 1 | 37 | 50 mgF / L |
| Q7TS74 | Cytoskeleton-associated protein 2-like | 119 | 50 mgF / L |
| Q99LN9 | Deoxyhypusine hydroxylase | 199 | 50 mgF / L |
| P31001 | Desmin | 211 | 50 mgF / L |
| O08749 | Dihydrolipoyl dehydrogenase, mitochondrial | 91 | 50 mgF / L |
| Q8BMF4 | Dihydrolipoyllysine-residue acetyltransferase component of pyruvate dehydrogenase complex, mitochondrial | 86 | 50 mgF / L |
| B1AZP2 | Disks large-associated protein 4 | 202 | 50 mgF / L |
| Q9D7K5 | Distal membrane-arm assembly complex protein 2 | 131 | 50 mgF / L |
| P33611 | DNA polymerase alpha subunit B | 73 | 50 mgF / L |
| Q6ZQF0 | DNA topoisomerase 2-binding protein 1 | 151 | 50 mgF / L |
| O35134 | DNA-directed RNA polymerase I subunit RPA1 | 100 | 50 mgF / L |
| Q921X6 | DNA-directed RNA polymerase III subunit RPC6 | 176 | 50 mgF / L |
| Q9R022 | DnaJ homolog subfamily C member 12 | 289 | 50 mgF / L |
| Q91YW3 | DnaJ homolog subfamily C member 3 | 206 | 50 mgF / L |
| Q05AA6 | Dystrophin-related protein 2 | 71 | 50 mgF / L |
| Q5DTM8 | E3 ubiquitin-protein ligase BRE1A | 107 | 50 mgF / L |
| Q8R516 | E3 ubiquitin-protein ligase MIB2 | 72 | 50 mgF / L |
| Q8R0K2 | E3 ubiquitin-protein ligase TRIM31 | 60 | 50 mgF / L |
| Q3UVK0 | Endoplasmic reticulum metallopeptidase 1 | 62 | 50 mgF / L |
| Q9D1Q6 | Endoplasmic reticulum resident protein 44 | 85 | 50 mgF / L |
| P42125 | Enoyl-CoA delta isomerase 1, mitochondrial | 167 | 50 mgF / L |
| O54839 | Eomesodermin homolog | 99 | 50 mgF / L |
| Q3UYR4 | Espin-like protein | 64 | 50 mgF / L |
| Q9QZ11 | Exonuclease 1 | 177 | 50 mgF / L |
| Q08943 | FACT complex subunit SSRP1 | 79 | 50 mgF / L |
| Q3UQN2 | F-BAR domain only protein 2 | 251 | 50 mgF / L |
| Q9EPX5 | F-box/LRR-repeat protein 12 | 153 | 50 mgF / L |
| Q8BHD4 | FERM domain-containing protein 3 | 74 | 50 mgF / L |
| Q8K0E8 | Fibrinogen beta chain | 290 | 50 mgF / L |
| Q8BUR3 | Forkhead box protein J3 | 99 | 50 mgF / L |
| Q61091 | Frizzled-8 | 84 | 50 mgF / L |
| Q810T2 | G2/mitotic-specific cyclin-B3 | 73 | 50 mgF / L |
| Q8VE33 | Ganglioside-induced differentiation-associated protein 1-like 1 | 121 | 50 mgF / L |
| Q8BL74 | General transcription factor 3C polypeptide 2 | 128 | 50 mgF / L |
| P03995 | Glial fibrillary acidic protein | 176 | 50 mgF / L |
| Q91X44 | Glucokinase regulatory protein | 77 | 50 mgF / L |
| P97324 | Glucose-6-phosphate 1-dehydrogenase 2 | 152 | 50 mgF / L |
| Q01097 | Glutamate receptor ionotropic, NMDA 2B | 107 | 50 mgF / L |
| Q8CHP8 | Glycerol-3-phosphate phosphatase | 70 | 50 mgF / L |
| Q9ET01 | Glycogen phosphorylase, liver form | 91 | 50 mgF / L |
| Q8K3J9 | G-protein coupled receptor family C group 5 member C | 951 | 50 mgF / L |
| Q80TI0 | GRAM domain-containing protein 1B | 87 | 50 mgF / L |
| Q03160 | Growth factor receptor-bound protein 7 | 80 | 50 mgF / L |
| P62827 | GTP-binding nuclear protein Ran | 310 | 50 mgF / L |
| Q61820 | GTP-binding nuclear protein Ran, testis-specific isoform | 67 | 50 mgF / L |
| P36536 | GTP-binding protein SAR1a | 256 | 50 mgF / L |
| Q9CQC9 | GTP-binding protein SAR1b | 174 | 50 mgF / L |
| Q9R0C8 | Guanine nucleotide exchange factor VAV3 | 78 | 50 mgF / L |
| P50149 | Guanine nucleotide-binding protein G(t) subunit alpha-2 | 79 | 50 mgF / L |
| P48722 | Heat shock 70 kDa protein 4L | 74 | 50 mgF / L |
| Q91X72 | Hemopexin | 155 | 50 mgF / L |
| P49312 | Heterogeneous nuclear ribonucleoprotein A1 | 248 | 50 mgF / L |
| O88569 | Heterogeneous nuclear ribonucleoproteins A2/B1 | 843 | 50 mgF / L |
| P70349 | Histidine triad nucleotide-binding protein 1 | 541 | 50 mgF / L |
| P79457 | Histone demethylase UTY | 183 | 50 mgF / L |
| P10922 | Histone H1.0 | 132 | 50 mgF / L |
| Q61188 | Histone-lysine N-methyltransferase EZH2 | 109 | 50 mgF / L |
| Q3U8K7 | Histone-lysine N-methyltransferase KMT5B | 286 | 50 mgF / L |
| P53564 | Homeobox protein cut-like 1 | 143 | 50 mgF / L |
| Q8R1H0 | Homeodomain-only protein | 152 | 50 mgF / L |
| Q61425 | Hydroxyacyl-coenzyme A dehydrogenase, mitochondrial | 582 | 50 mgF / L |
| P03975 | IgE-binding protein | 380 | 50 mgF / L |
| P15975 | Inactive ubiquitin carboxyl-terminal hydrolase 53 | 179 | 50 mgF / L |
| Q9D8Y8 | Inhibitor of growth protein 5 | 294 | 50 mgF / L |
| Q9Z329 | Inositol 1,4,5-trisphosphate receptor type 2 | 118 | 50 mgF / L |
| A2ARA8 | Integrin alpha-8 | 92 | 50 mgF / L |
| Q80SU7 | Interferon-induced very large GTPase 1 | 133 | 50 mgF / L |
| P19182 | Interferon-related developmental regulator 1 | 147 | 50 mgF / L |
| Q9Z0R4 | Intersectin-1 | 96 | 50 mgF / L |
| Q6VH22 | Intraflagellar transport protein 172 homolog | 67 | 50 mgF / L |
| Q5DTN8 | Janus kinase and microtubule-interacting protein 3 | 72 | 50 mgF / L |
| Q61765 | Keratin, type I cuticular Ha1 | 134 | 50 mgF / L |
| Q62168 | Keratin, type I cuticular Ha2 | 134 | 50 mgF / L |
| Q61897 | Keratin, type I cuticular Ha3-II | 134 | 50 mgF / L |
| Q497I4 | Keratin, type I cuticular Ha5 | 134 | 50 mgF / L |
| B1AQ75 | Keratin, type I cuticular Ha6 | 134 | 50 mgF / L |
| P02535 | Keratin, type I cytoskeletal 10 | 134 | 50 mgF / L |
| Q9Z2K1 | Keratin, type I cytoskeletal 16 | 68 | 50 mgF / L |
| A1L317 | Keratin, type I cytoskeletal 24 | 134 | 50 mgF / L |
| A6BLY7 | Keratin, type I cytoskeletal 28 | 152 | 50 mgF / L |
| Q6IFX3 | Keratin, type I cytoskeletal 40 | 134 | 50 mgF / L |
| Q6IMF0 | Keratin, type II cuticular 87 | 176 | 50 mgF / L |
| Q9ERE2 | Keratin, type II cuticular Hb1 | 176 | 50 mgF / L |
| Q9Z2T6 | Keratin, type II cuticular Hb5 | 176 | 50 mgF / L |
| P97861 | Keratin, type II cuticular Hb6 | 176 | 50 mgF / L |
| A2A9C3 | KICSTOR complex protein SZT2 | 66 | 50 mgF / L |
| O08672 | Kinesin-like protein KIFC2 | 92 | 50 mgF / L |
| Q8BGA5 | KRR1 small subunit processome component homolog | 64 | 50 mgF / L |
| Q80ST9 | Lebercilin | 75 | 50 mgF / L |
| Q8R4U7 | Leucine zipper protein 1 | 154 | 50 mgF / L |
| Q8BFW7 | Lipoma-preferred partner homolog | 76 | 50 mgF / L |
| Q61805 | Lipopolysaccharide-binding protein | 144 | 50 mgF / L |
| Q8BSS9 | Liprin-alpha-2 | 90 | 50 mgF / L |
| O35711 | Liprin-beta-2 | 139 | 50 mgF / L |
| P41230 | Lysine-specific demethylase 5C | 229 | 50 mgF / L |
| Q99MN1 | Lysine--tRNA ligase | 128 | 50 mgF / L |
| P70699 | Lysosomal alpha-glucosidase | 134 | 50 mgF / L |
| Q62190 | Macrophage-stimulating protein receptor | 73 | 50 mgF / L |
| Q9EQK5 | Major vault protein | 120 | 50 mgF / L |
| Q0PMG2 | MAM domain-containing glycosylphosphatidylinositol anchor protein 1 | 74 | 50 mgF / L |
| Q924M7 | Mannose-6-phosphate isomerase | 141 | 50 mgF / L |
| O70423 | Membrane primary amine oxidase | 68 | 50 mgF / L |
| O55022 | Membrane-associated progesterone receptor component 1 | 250 | 50 mgF / L |
| P70669 | Metalloendopeptidase homolog PEX | 168 | 50 mgF / L |
| Q3TY92 | Methyl-CpG-binding domain protein 6 | 154 | 50 mgF / L |
| Q8BG87 | Methylcytosine dioxygenase TET3 | 78 | 50 mgF / L |
| P16332 | Methylmalonyl-CoA mutase, mitochondrial | 90 | 50 mgF / L |
| Q922T2 | Microfibril-associated glycoprotein 3 | 210 | 50 mgF / L |
| P10637 | Microtubule-associated protein tau | 96 | 50 mgF / L |
| Q9D071 | MMS19 nucleotide excision repair protein homolog | 212 | 50 mgF / L |
| Q80WJ6 | Multidrug resistance-associated protein 9 | 63 | 50 mgF / L |
| Q5RJH2 | Multiple C2 and transmembrane domain-containing protein 2 | 59 | 50 mgF / L |
| Q61006 | Muscle, skeletal receptor tyrosine-protein kinase | 75 | 50 mgF / L |
| Q80YT7 | Myomegalin | 129 | 50 mgF / L |
| Q8BMF3 | NADP-dependent malic enzyme, mitochondrial | 100 | 50 mgF / L |
| Q60817 | Nascent polypeptide-associated complex subunit alpha | 72 | 50 mgF / L |
| P70670 | Nascent polypeptide-associated complex subunit alpha, muscle-specific form | 94 | 50 mgF / L |
| Q4FZC9 | Nesprin-3 | 63 | 50 mgF / L |
| Q6ZQA0 | Neurobeachin-like protein 2 | 51 | 50 mgF / L |
| P21661 | Neuroendocrine convertase 2 | 155 | 50 mgF / L |
| P19246 | Neurofilament heavy polypeptide | 59 | 50 mgF / L |
| P08551 | Neurofilament light polypeptide | 64 | 50 mgF / L |
| P08553 | Neurofilament medium polypeptide | 75 | 50 mgF / L |
| Q99K10 | Neuroligin-1 | 94 | 50 mgF / L |
| P97333 | Neuropilin-1 | 87 | 50 mgF / L |
| Q8BVW0 | Neutral alpha-glucosidase C | 62 | 50 mgF / L |
| P29477 | Nitric oxide synthase, inducible | 82 | 50 mgF / L |
| Q3UP24 | NLR family CARD domain-containing protein 4 | 63 | 50 mgF / L |
| Q9CZA6 | Nuclear distribution protein nudE homolog 1 | 192 | 50 mgF / L |
| Q8R0G9 | Nuclear pore complex protein Nup133 | 54 | 50 mgF / L |
| Q9WU42 | Nuclear receptor corepressor 2 | 242 | 50 mgF / L |
| Q6PIP5 | NudC domain-containing protein 1 | 79 | 50 mgF / L |
| P34983 | Olfactory receptor 1537 | 140 | 50 mgF / L |
| P29758 | Ornithine aminotransferase, mitochondrial | 74 | 50 mgF / L |
| A2AJ88 | Patatin-like phospholipase domain-containing protein 7 | 204 | 50 mgF / L |
| B9EJ80 | PDZ domain-containing protein 8 | 66 | 50 mgF / L |
| Q6RUU0 | Pentraxin-4 | 83 | 50 mgF / L |
| Q64378 | Peptidyl-prolyl cis-trans isomerase FKBP5 | 142 | 50 mgF / L |
| O54943 | Period circadian protein homolog 2 | 84 | 50 mgF / L |
| P15331 | Peripherin | 119 | 50 mgF / L |
| Q9Z280 | Phospholipase D1 | 121 | 50 mgF / L |
| P97813 | Phospholipase D2 | 321 | 50 mgF / L |
| Q8BLJ3 | PI-PLC X domain-containing protein 3 | 125 | 50 mgF / L |
| P48678 | Prelamin-A/C | 124 | 50 mgF / L |
| Q8BHJ9 | Pre-mRNA-splicing factor SLU7 | 109 | 50 mgF / L |
| Q3UX83 | Probable inactive 1-aminocyclopropane-1-carboxylate synthase-like protein 2 | 110 | 50 mgF / L |
| O35129 | Prohibitin-2 | 191 | 50 mgF / L |
| Q99MN9 | Propionyl-CoA carboxylase beta chain, mitochondrial | 110 | 50 mgF / L |
| Q8VCR7 | Protein ABHD14B | 513 | 50 mgF / L |
| P70403 | Protein CASP | 153 | 50 mgF / L |
| Q8CJF7 | Protein ELYS | 119 | 50 mgF / L |
| Q9D6I7 | Protein FAM69A | 83 | 50 mgF / L |
| Q8BPI1 | Protein kintoun | 69 | 50 mgF / L |
| P33215 | Protein NEDD1 | 60 | 50 mgF / L |
| O55126 | Protein NipSnap homolog 2 | 323 | 50 mgF / L |
| Q9D281 | Protein Noxp20 | 66 | 50 mgF / L |
| Q3UMT1 | Protein phosphatase 1 regulatory subunit 12C | 54 | 50 mgF / L |
| Q8BVT6 | Protein phosphatase 2C-like domain-containing protein 1 | 112 | 50 mgF / L |
| Q99K43 | Protein regulator of cytokinesis 1 | 68 | 50 mgF / L |
| P14069 | Protein S100-A6 | 368 | 50 mgF / L |
| Q99KD5 | Protein unc-45 homolog A | 52 | 50 mgF / L |
| Q8BZ32 | Putative Polycomb group protein ASXL2 | 88 | 50 mgF / L |
| P35486 | Pyruvate dehydrogenase E1 component subunit alpha, somatic form, mitochondrial | 266 | 50 mgF / L |
| Q80Y56 | Rabenosyn-5 | 140 | 50 mgF / L |
| Q9QUG9 | RAS guanyl-releasing protein 2 | 108 | 50 mgF / L |
| Q8C2K5 | RAS protein activator like-3 | 85 | 50 mgF / L |
| Q9Z268 | RasGAP-activating-like protein 1 | 58 | 50 mgF / L |
| B2RU80 | Receptor-type tyrosine-protein phosphatase beta | 54 | 50 mgF / L |
| Q05909 | Receptor-type tyrosine-protein phosphatase gamma | 75 | 50 mgF / L |
| Q99NE5 | Regulating synaptic membrane exocytosis protein 1 | 134 | 50 mgF / L |
| Q9Z2H1 | Regulator of G-protein signaling 11 | 146 | 50 mgF / L |
| Q8BH78 | Reticulon | 54 | 50 mgF / L |
| Q99P72 | Reticulon-4 | 104 | 50 mgF / L |
| Q62148 | Retinal dehydrogenase 2 | 145 | 50 mgF / L |
| O35600 | Retinal-specific ATP-binding cassette transporter | 63 | 50 mgF / L |
| Q61599 | Rho GDP-dissociation inhibitor 2 | 354 | 50 mgF / L |
| A2AWP8 | Rho guanine nucleotide exchange factor 10-like protein | 64 | 50 mgF / L |
| Q8R4H2 | Rho guanine nucleotide exchange factor 12 | 238 | 50 mgF / L |
| Q7TNR9 | Rho guanine nucleotide exchange factor 4 | 218 | 50 mgF / L |
| Q91VI7 | Ribonuclease inhibitor | 218 | 50 mgF / L |
| P07742 | Ribonucleoside-diphosphate reductase large subunit | 144 | 50 mgF / L |
| P18653 | Ribosomal protein S6 kinase alpha-1 | 135 | 50 mgF / L |
| Q9WUT3 | Ribosomal protein S6 kinase alpha-2 | 173 | 50 mgF / L |
| P18654 | Ribosomal protein S6 kinase alpha-3 | 163 | 50 mgF / L |
| Q149F1 | RNA pseudouridylate synthase domain-containing protein 2 | 133 | 50 mgF / L |
| Q9QX96 | Sal-like protein 2 | 76 | 50 mgF / L |
| Q3URD3 | Sarcolemmal membrane-associated protein | 184 | 50 mgF / L |
| P29621 | Serine protease inhibitor A3C | 669 | 50 mgF / L |
| Q80X76 | Serine protease inhibitor A3F | 636 | 50 mgF / L |
| P70458 | Serine protease inhibitor A3G | 138 | 50 mgF / L |
| Q91WP6 | Serine protease inhibitor A3N | 645 | 50 mgF / L |
| Q9QZX7 | Serine racemase | 63 | 50 mgF / L |
| P84104 | Serine/arginine-rich splicing factor 3 | 123 | 50 mgF / L |
| P83741 | Serine/threonine-protein kinase WNK1 | 58 | 50 mgF / L |
| Q80UE6 | Serine/threonine-protein kinase WNK4 | 126 | 50 mgF / L |
| Q80W00 | Serine/threonine-protein phosphatase 1 regulatory subunit 10 | 146 | 50 mgF / L |
| P19324 | Serpin H1 | 86 | 50 mgF / L |
| Q62087 | Serum paraoxonase/lactonase 3 | 171 | 50 mgF / L |
| Q8BMC3 | SHC-transforming protein 2 | 135 | 50 mgF / L |
| Q91Y57 | Sialic acid-binding Ig-like lectin 12 | 63 | 50 mgF / L |
| Q9JMH7 | Sialidase-3 | 107 | 50 mgF / L |
| Q6ZWQ7 | Signal peptidase complex subunit 3 | 141 | 50 mgF / L |
| P47758 | Signal recognition particle receptor subunit beta | 81 | 50 mgF / L |
| P42225 | Signal transducer and activator of transcription 1 | 134 | 50 mgF / L |
| Q8BHY8 | Sorting nexin-14 | 81 | 50 mgF / L |
| Q9CZV5 | STAGA complex 65 subunit gamma | 95 | 50 mgF / L |
| F6XZJ7 | Sterile alpha motif domain-containing protein 15 | 78 | 50 mgF / L |
| Q62209 | Synaptonemal complex protein 1 | 97 | 50 mgF / L |
| Q9R0N4 | Synaptotagmin-10 | 99 | 50 mgF / L |
| Q71LX4 | Talin-2 | 89 | 50 mgF / L |
| Q3UES3 | Tankyrase-2 | 70 | 50 mgF / L |
| O88746 | Target of Myb protein 1 | 276 | 50 mgF / L |
| Q8BM85 | TBC domain-containing protein kinase-like protein | 63 | 50 mgF / L |
| P42932 | T-complex protein 1 subunit theta | 126 | 50 mgF / L |
| Q9D494 | Telomere repeats-binding bouquet formation protein 2 | 172 | 50 mgF / L |
| Q8CC21 | Tetratricopeptide repeat protein 19, mitochondrial | 139 | 50 mgF / L |
| Q91W90 | Thioredoxin domain-containing protein 5 | 343 | 50 mgF / L |
| Q8BH58 | TIP41-like protein | 100 | 50 mgF / L |
| P70191 | TNF receptor-associated factor 5 | 108 | 50 mgF / L |
| Q5NC05 | Transcription termination factor 2 | 55 | 50 mgF / L |
| Q9JJG0 | Transforming acidic coiled-coil-containing protein 2 | 79 | 50 mgF / L |
| Q91YD4 | Transient receptor potential cation channel subfamily M member 2 | 110 | 50 mgF / L |
| Q9D4D4 | Transketolase-like protein 2 | 131 | 50 mgF / L |
| Q62186 | Translocon-associated protein subunit delta | 146 | 50 mgF / L |
| Q8BH24 | Transmembrane 9 superfamily member 4 | 227 | 50 mgF / L |
| Q99KF1 | Transmembrane emp24 domain-containing protein 9 | 235 | 50 mgF / L |
| P20801 | Troponin C, skeletal muscle | 139 | 50 mgF / L |
| Q3UX10 | Tubulin alpha chain-like 3 | 84 | 50 mgF / L |
| P83887 | Tubulin gamma-1 chain | 61 | 50 mgF / L |
| Q8VCK3 | Tubulin gamma-2 chain | 111 | 50 mgF / L |
| Q99NB8 | Ubiquilin-4 | 226 | 50 mgF / L |
| Q6P5E4 | UDP-glucose:glycoprotein glucosyltransferase 1 | 171 | 50 mgF / L |
| Q5SYD0 | Unconventional myosin-Id | 58 | 50 mgF / L |
| E9Q634 | Unconventional myosin-Ie | 77 | 50 mgF / L |
| Q9QZZ4 | Unconventional myosin-XV | 40 | 50 mgF / L |
| Q2QI47 | Usherin | 112 | 50 mgF / L |
| P49766 | Vascular endothelial growth factor B | 111 | 50 mgF / L |
| Q62059 | Versican core protein | 112 | 50 mgF / L |
| P21614 | Vitamin D-binding protein | 75 | 50 mgF / L |
| P62814 | V-type proton ATPase subunit B, brain isoform | 98 | 50 mgF / L |
| Q9JHY3 | WAP four-disulfide core domain protein 12 | 1089 | 50 mgF / L |
| O88532 | Zinc finger RNA-binding protein | 64 | 50 mgF / L |
| Q64433 | 10 kDa heat shock protein, mitochondrial | 194 | 10 mgF / L |
| Q05921 | 2-5A-dependent ribonuclease | 62 | 10 mgF / L |
| P26516 | 26S proteasome non-ATPase regulatory subunit 7 | 93 | 10 mgF / L |
| Q9D110 | 5-formyltetrahydrofolate cyclo-ligase | 313 | 10 mgF / L |
| P14148 | 60S ribosomal protein L7 | 78 | 10 mgF / L |
| P51410 | 60S ribosomal protein L9 | 461 | 10 mgF / L |
| Q4JIM5 | Abelson tyrosine-protein kinase 2 | 123 | 10 mgF / L |
| Q9EST5 | Acidic leucine-rich nuclear phosphoprotein 32 family member B | 205 | 10 mgF / L |
| Q8CG27 | Actin-like protein 9 | 93 | 10 mgF / L |
| Q5SSL4 | Active breakpoint cluster region-related protein | 129 | 10 mgF / L |
| P31786 | Acyl-CoA-binding protein | 1023 | 10 mgF / L |
| P50247 | Adenosylhomocysteinase | 152 | 10 mgF / L |
| Q9WUR9 | Adenylate kinase 4, mitochondrial | 189 | 10 mgF / L |
| Q60662 | A-kinase anchor protein 4 | 58 | 10 mgF / L |
| P28474 | Alcohol dehydrogenase class-3 | 681 | 10 mgF / L |
| E9Q3E1 | Aldehyde dehydrogenase family 3 member B2 | 94 | 10 mgF / L |
| P19091 | Androgen receptor | 67 | 10 mgF / L |
| Q99NH0 | Ankyrin repeat domain-containing protein 17 | 70 | 10 mgF / L |
| Q5SUE8 | Ankyrin repeat domain-containing protein 40 | 67 | 10 mgF / L |
| Q00623 | Apolipoprotein A-I | 321 | 10 mgF / L |
| Q9QWY8 | Arf-GAP with SH3 domain, ANK repeat and PH domain-containing protein 1 | 92 | 10 mgF / L |
| Q9D0I9 | Arginine--tRNA ligase, cytoplasmic | 65 | 10 mgF / L |
| Q91WU5 | Arsenite methyltransferase | 102 | 10 mgF / L |
| Q61137 | Astrotactin-1 | 195 | 10 mgF / L |
| Q9CQQ7 | ATP synthase F(0) complex subunit B1, mitochondrial | 302 | 10 mgF / L |
| O70133 | ATP-dependent RNA helicase A | 88 | 10 mgF / L |
| O88967 | ATP-dependent zinc metalloprotease YME1L1 | 69 | 10 mgF / L |
| P97477 | Aurora kinase A | 63 | 10 mgF / L |
| P70444 | BH3-interacting domain death agonist | 267 | 10 mgF / L |
| Q91WG8 | Bifunctional UDP-N-acetylglucosamine 2-epimerase/N-acetylmannosamine kinase | 166 | 10 mgF / L |
| Q8BFX3 | BTB/POZ domain-containing protein KCTD3 | 74 | 10 mgF / L |
| P16015 | Carbonic anhydrase 3 | 417 | 10 mgF / L |
| Q9WVJ3 | Carboxypeptidase Q | 98 | 10 mgF / L |
| Q6A068 | Cell division cycle 5-like protein | 49 | 10 mgF / L |
| Q3UXL4 | Centrosomal protein kizuna | 105 | 10 mgF / L |
| Q8BI22 | Centrosomal protein of 128 kDa | 264 | 10 mgF / L |
| Q9D5D8 | Chromodomain Y-like protein 2 | 152 | 10 mgF / L |
| Q8BRT1 | CLIP-associating protein 2 | 176 | 10 mgF / L |
| Q3THF9 | Coenzyme Q-binding protein COQ10 homolog B, mitochondrial | 210 | 10 mgF / L |
| Q9D5Y1 | Coiled-coil domain-containing protein 39 | 70 | 10 mgF / L |
| Q64739 | Collagen alpha-2(XI) chain | 42 | 10 mgF / L |
| Q9QZS0 | Collagen alpha-3(IV) chain | 234 | 10 mgF / L |
| Q8R066 | Complement C1q tumor necrosis factor-related protein 4 | 88 | 10 mgF / L |
| P06684 | Complement C5 | 52 | 10 mgF / L |
| Q9JMB8 | Contactin-6 | 48 | 10 mgF / L |
| Q8VE73 | Cullin-7 | 56 | 10 mgF / L |
| Q91WL5 | Cytochrome P450 4A12A | 65 | 10 mgF / L |
| A2RSQ0 | DENN domain-containing protein 5B | 84 | 10 mgF / L |
| F8WJE0 | Deoxynucleoside triphosphate triphosphohydrolase | 52 | 10 mgF / L |
| Q60710 | Deoxynucleoside triphosphate triphosphohydrolase SAMHD1 | 49 | 10 mgF / L |
| Q8R2M2 | Deoxynucleotidyltransferase terminal-interacting protein 2 | 48 | 10 mgF / L |
| P49183 | Deoxyribonuclease-1 | 5966 | 10 mgF / L |
| Q9D7J6 | Deoxyribonuclease-1-like 1 | 80 | 10 mgF / L |
| Q91YP3 | Deoxyribose-phosphate aldolase | 86 | 10 mgF / L |
| Q8BH86 | D-glutamate cyclase, mitochondrial | 56 | 10 mgF / L |
| Q8BVG4 | Dipeptidyl peptidase 9 | 55 | 10 mgF / L |
| Q9JIC3 | DNA cross-link repair 1A protein | 65 | 10 mgF / L |
| Q9JJN0 | DNA polymerase eta | 51 | 10 mgF / L |
| Q8BKF1 | DNA-directed RNA polymerase, mitochondrial | 48 | 10 mgF / L |
| Q91YQ5 | Dolichyl-diphosphooligosaccharide--protein glycosyltransferase subunit 1 | 148 | 10 mgF / L |
| Q9CZ00 | Dysbindin domain-containing protein 1 | 177 | 10 mgF / L |
| E9Q555 | E3 ubiquitin-protein ligase RNF213 | 83 | 10 mgF / L |
| Q99MS7 | EH domain-binding protein 1-like protein 1 | 90 | 10 mgF / L |
| Q60900 | ELAV-like protein 3 | 114 | 10 mgF / L |
| Q61772 | Ephrin type-A receptor 7 | 66 | 10 mgF / L |
| P42567 | Epidermal growth factor receptor substrate 15 | 61 | 10 mgF / L |
| Q03146 | Epithelial discoidin domain-containing receptor 1 | 114 | 10 mgF / L |
| Q9D172 | ES1 protein homolog, mitochondrial | 196 | 10 mgF / L |
| P50171 | Estradiol 17-beta-dehydrogenase 8 | 140 | 10 mgF / L |
| Q9QZD9 | Eukaryotic translation initiation factor 3 subunit I | 90 | 10 mgF / L |
| P22315 | Ferrochelatase, mitochondrial | 94 | 10 mgF / L |
| Q8BX90 | Fibronectin type-III domain-containing protein 3A | 98 | 10 mgF / L |
| Q8VHX6 | Filamin-C | 83 | 10 mgF / L |
| Q6P9Q6 | FK506-binding protein 15 | 142 | 10 mgF / L |
| P42128 | Forkhead box protein K1 | 70 | 10 mgF / L |
| Q8VEB1 | G protein-coupled receptor kinase 5 | 63 | 10 mgF / L |
| Q8BKN5 | Gamma-tubulin complex component 5 | 89 | 10 mgF / L |
| P08752 | Guanine nucleotide-binding protein G(i) subunit alpha-2 | 111 | 10 mgF / L |
| Q9EQ15 | Guanine nucleotide-binding protein subunit beta-like protein 1 | 98 | 10 mgF / L |
| Q61418 | H(+)/Cl(-) exchange transporter 4 | 372 | 10 mgF / L |
| Q8BM72 | Heat shock 70 kDa protein 13 | 353 | 10 mgF / L |
| O08755 | Hepatocyte nuclear factor 6 | 86 | 10 mgF / L |
| P51859 | Hepatoma-derived growth factor | 459 | 10 mgF / L |
| Q9Z2X1 | Heterogeneous nuclear ribonucleoprotein F | 92 | 10 mgF / L |
| Q8BVE8 | Histone-lysine N-methyltransferase NSD2 | 46 | 10 mgF / L |
| P01864 | Ig gamma-2A chain C region secreted form | 136 | 10 mgF / L |
| Q7TQA1 | Immunoglobulin superfamily member 1 | 47 | 10 mgF / L |
| Q61249 | Immunoglobulin-binding protein 1 | 81 | 10 mgF / L |
| Q9QUM0 | Integrin alpha-IIb | 53 | 10 mgF / L |
| Q8R460 | Interleukin-36 gamma | 105 | 10 mgF / L |
| Q811J3 | Iron-responsive element-binding protein 2 | 104 | 10 mgF / L |
| Q80TG1 | KAT8 regulatory NSL complex subunit 1 | 60 | 10 mgF / L |
| Q80W68 | Kin of IRRE-like protein 1 | 189 | 10 mgF / L |
| Q8C0N1 | Kinesin-like protein KIF2B | 61 | 10 mgF / L |
| Q9EQR5 | Lck-interacting transmembrane adapter 1 | 119 | 10 mgF / L |
| Q99PH1 | Leucine-rich repeat-containing protein 4 | 57 | 10 mgF / L |
| P0C192 | Leucine-rich repeat-containing protein 4B | 64 | 10 mgF / L |
| P51885 | Lumican | 105 | 10 mgF / L |
| P41245 | Matrix metalloproteinase-9 | 53 | 10 mgF / L |
| Q9CXI5 | Mesencephalic astrocyte-derived neurotrophic factor | 706 | 10 mgF / L |
| Q8C7H1 | Methylmalonic aciduria type A homolog, mitochondrial | 125 | 10 mgF / L |
| P48377 | MHC class II regulatory factor RFX1 | 63 | 10 mgF / L |
| Q7TT79 | Microcephalin | 84 | 10 mgF / L |
| Q6P5G0 | Mitogen-activated protein kinase 4 | 126 | 10 mgF / L |
| Q9WTX8 | Mitotic spindle assembly checkpoint protein MAD1 | 73 | 10 mgF / L |
| Q8VBX6 | Multiple PDZ domain protein | 92 | 10 mgF / L |
| Q6KAU4 | Multivesicular body subunit 12B | 182 | 10 mgF / L |
| P28665 | Murinoglobulin-1 | 103 | 10 mgF / L |
| Q6NZR2 | Myb/SANT-like DNA-binding domain-containing protein 2 | 67 | 10 mgF / L |
| P11247 | Myeloperoxidase | 150 | 10 mgF / L |
| P09541 | Myosin light chain 4 | 132 | 10 mgF / L |
| Q5SX39 | Myosin-4 | 73 | 10 mgF / L |
| P13542 | Myosin-8 | 73 | 10 mgF / L |
| Q9DCS9 | NADH dehydrogenase [ubiquinone] 1 beta subcomplex subunit 10 | 117 | 10 mgF / L |
| Q8K1S3 | Netrin receptor UNC5B | 47 | 10 mgF / L |
| Q8CH77 | Neuron navigator 1 | 61 | 10 mgF / L |
| Q3TRM4 | Neuropathy target esterase | 152 | 10 mgF / L |
| P12813 | Nuclear receptor subfamily 4 group A member 1 | 48 | 10 mgF / L |
| Q02819 | Nucleobindin-1 | 23 | 10 mgF / L |
| Q9JIK5 | Nucleolar RNA helicase 2 | 62 | 10 mgF / L |
| Q06348 | Paired mesoderm homeobox protein 2 | 96 | 10 mgF / L |
| Q3ULF4 | Paraplegin | 238 | 10 mgF / L |
| Q8CEE6 | PAS domain-containing serine/threonine-protein kinase | 52 | 10 mgF / L |
| P58501 | PAX3- and PAX7-binding protein 1 | 65 | 10 mgF / L |
| Q7TNF8 | Peripheral-type benzodiazepine receptor-associated protein 1 | 37 | 10 mgF / L |
| Q8CBQ5 | Phosphatidylinositol 4-kinase type 2-beta | 281 | 10 mgF / L |
| Q8BH04 | Phosphoenolpyruvate carboxykinase [GTP], mitochondrial | 90 | 10 mgF / L |
| Q8K212 | Phosphofurin acidic cluster sorting protein 1 | 58 | 10 mgF / L |
| Q68FH0 | Plakophilin-4 | 64 | 10 mgF / L |
| Q6Q477 | Plasma membrane calcium-transporting ATPase 4 | 46 | 10 mgF / L |
| Q99K51 | Plastin-3 | 140 | 10 mgF / L |
| P17225 | Polypyrimidine tract-binding protein 1 | 121 | 10 mgF / L |
| Q9WVJ0 | Potassium voltage-gated channel subfamily H member 3 | 63 | 10 mgF / L |
| Q8K4P0 | pre-mRNA 3' end processing protein WDR33 | 142 | 10 mgF / L |
| Q501J6 | Probable ATP-dependent RNA helicase DDX17 | 113 | 10 mgF / L |
| Q8K301 | Probable ATP-dependent RNA helicase DDX52 | 63 | 10 mgF / L |
| Q99PJ2 | Probable E3 ubiquitin-protein ligase TRIM8 | 72 | 10 mgF / L |
| P99026 | Proteasome subunit beta type-4 | 86 | 10 mgF / L |
| Q6PDI5 | Proteasome-associated protein ECM29 homolog | 51 | 10 mgF / L |
| Q9DB52 | Protein FAM122A | 70 | 10 mgF / L |
| Q8C729 | Protein FAM126B | 205 | 10 mgF / L |
| Q5SXA9 | Protein KIBRA | 49 | 10 mgF / L |
| Q8CGC4 | Protein LSM14 homolog B | 111 | 10 mgF / L |
| Q80WJ7 | Protein LYRIC | 84 | 10 mgF / L |
| A4Q9F1 | Protein monoglycylase TTLL8 | 136 | 10 mgF / L |
| Q7TPM1 | Protein PRRC2B | 87 | 10 mgF / L |
| Q8K3V4 | Protein-arginine deiminase type-6 | 63 | 10 mgF / L |
| P19221 | Prothrombin | 137 | 10 mgF / L |
| Q8VIG3 | Radial spoke head 1 homolog | 58 | 10 mgF / L |
| Q8CI78 | Required for meiotic nuclear division protein 1 homolog | 64 | 10 mgF / L |
| P28704 | Retinoic acid receptor RXR-beta | 134 | 10 mgF / L |
| Q8VCH7 | Retinol dehydrogenase 10 | 83 | 10 mgF / L |
| Q91YM2 | Rho GTPase-activating protein 35 | 106 | 10 mgF / L |
| Q5FWH6 | Rho guanine nucleotide exchange factor 15 | 66 | 10 mgF / L |
| Q8BWA8 | Rho guanine nucleotide exchange factor 19 | 220 | 10 mgF / L |
| Q9Z1M4 | Ribosomal protein S6 kinase beta-2 | 553 | 10 mgF / L |
| P56959 | RNA-binding protein FUS | 226 | 10 mgF / L |
| A7XUY5 | Selection and upkeep of intraepithelial T-cells protein 5 | 69 | 10 mgF / L |
| O88632 | Semaphorin-3F | 142 | 10 mgF / L |
| Q62179 | Semaphorin-4B | 94 | 10 mgF / L |
| O09126 | Semaphorin-4D | 64 | 10 mgF / L |
| Q8BHI9 | Serine/threonine-protein kinase NIM1 | 151 | 10 mgF / L |
| Q922R5 | Serine/threonine-protein phosphatase 4 regulatory subunit 3B | 89 | 10 mgF / L |
| Q6S5L9 | SHC-transforming protein 4 | 72 | 10 mgF / L |
| Q99LM3 | Smoothelin-like protein 1 | 112 | 10 mgF / L |
| Q91XA5 | snRNA-activating protein complex subunit 2 | 100 | 10 mgF / L |
| Q80SU6 | Sodium-dependent phosphate transport protein 2C | 78 | 10 mgF / L |
| P06880 | Somatotropin | 125 | 10 mgF / L |
| Q9D5R4 | Spermatogenesis-associated protein 1 | 75 | 10 mgF / L |
| P52019 | Squalene monooxygenase | 130 | 10 mgF / L |
| Q80TF6 | StAR-related lipid transfer protein 9 | 81 | 10 mgF / L |
| Q9Z2I8 | Succinate--CoA ligase [GDP-forming] subunit beta, mitochondrial | 233 | 10 mgF / L |
| Q9D0K2 | Succinyl-CoA:3-ketoacid coenzyme A transferase 1, mitochondrial | 61 | 10 mgF / L |
| O70439 | Syntaxin-7 | 82 | 10 mgF / L |
| Q8R2K4 | TAF6-like RNA polymerase II p300/CBP-associated factor-associated factor 65 kDa subunit 6L | 55 | 10 mgF / L |
| Q921F2 | TAR DNA-binding protein 43 | 355 | 10 mgF / L |
| B9EKI3 | TATA element modulatory factor | 69 | 10 mgF / L |
| P80315 | T-complex protein 1 subunit delta | 78 | 10 mgF / L |
| Q8BTG3 | T-complex protein 11-like protein 1 | 102 | 10 mgF / L |
| P10639 | Thioredoxin | 271 | 10 mgF / L |
| P97770 | THUMP domain-containing protein 3 | 107 | 10 mgF / L |
| Q3TKT4 | Transcription activator BRG1 | 40 | 10 mgF / L |
| Q60722 | Transcription factor 4 | 113 | 10 mgF / L |
| Q704Y3 | Transient receptor potential cation channel subfamily V member 1 | 72 | 10 mgF / L |
| Q7TN60 | Transmembrane channel-like protein 6 | 730 | 10 mgF / L |
| Q9D2R4 | Transmembrane emp24 domain-containing protein 11 | 46 | 10 mgF / L |
| Q62393 | Tumor protein D52 | 357 | 10 mgF / L |
| Q62120 | Tyrosine-protein kinase JAK2 | 45 | 10 mgF / L |
| Q5DU02 | Ubiquitin carboxyl-terminal hydrolase 22 | 87 | 10 mgF / L |
| Q8BWR4 | Ubiquitin carboxyl-terminal hydrolase 40 | 100 | 10 mgF / L |
| Q9ES00 | Ubiquitin conjugation factor E4 B | 101 | 10 mgF / L |
| Q8BGG7 | Ubiquitin-associated and SH3 domain-containing protein B | 68 | 10 mgF / L |
| Q8R3I9 | UDP-GlcNAc:betaGal beta-1,3-N-acetylglucosaminyltransferase 8 | 250 | 10 mgF / L |
| Q9DBP5 | UMP-CMP kinase | 230 | 10 mgF / L |
| Q60930 | Voltage-dependent anion-selective channel protein 2 | 152 | 10 mgF / L |
| Q8CC27 | Voltage-dependent L-type calcium channel subunit beta-2 | 105 | 10 mgF / L |
| Q9Z1G4 | V-type proton ATPase 116 kDa subunit a isoform 1 | 65 | 10 mgF / L |
| Q8BND3 | WD repeat-containing protein 35 | 46 | 10 mgF / L |
| E9PYY5 | WD repeat-containing protein 78 | 55 | 10 mgF / L |
| Q8K088 | Zinc finger and BTB domain-containing protein 6 | 92 | 10 mgF / L |
| Q571J5 | Zinc finger protein 354C | 78 | 10 mgF / L |
| Q6NV66 | Zinc finger protein 646 | 87 | 10 mgF / L |
| Q75N73 | Zinc transporter ZIP14 | 188 | 10 mgF / L |

^a^Identification is based on proteins ID from UniProt protein database, reviewed only (<http://www.uniprot.org/>).

^b^ Proteins with expression significantly altered are organized according to the ratio

*Indicates unique proteins in alphabetical order.

**Table S4.** Proteins regulated according to the Over-Representation Analysis (ORA) with more PPI interactions in the submandibular glands for the comparisons: 10 mg F / L vs. Control, 50 mg F / L vs. Control and 50 mg F / L vs. 10 mg F / L.

| **Accession ID^a^** | **Protein name** | **10 mg F/L *vs.* control** | **50 mg F/L *vs.* control** | **50 mg F/L *vs.* 10 mg F/L** |
| --- | --- | --- | --- | --- |
| B2RQC6 | CAD protein | - | Down | UP |
| O70456 | 14-3-3 protein sigma | Up | Up | Down |
| O88569 | Heterogeneous nuclear ribonucleoproteins A2/B1 | Up | Down | Up |
| P05213 | Tubulin alpha-1B chain | Up | - | Down |
| P07901 | Heat shock protein HSP 90-alpha | - | - | Down |
| P08113 | Endoplasmin | Up | Down | Down |
| P08752 | Guanine nucleotide-binding protein G(i) subunit alpha-2 | - | UP | Down |
| P0c0s6 | Histone H2A.Z | Up | Down | Down |
| P0cg49 | Polyubiquitin-B | Up | Down | Down |
| P10126 | Elongation factor 1-alpha 1 | - | Down | Down |
| P11440 | Cyclin-dependent kinase 1 | Down | Down | - |
| P11499 | Heat shock protein hsp 90-beta | - | Down | Down |
| P13541 | Myosin-3 | Down | Down | - |
| P14869 | 60S acidic ribosomal protein P0 | Up | Down | Down |
| P16627 | Heat shock 70 kDa protein 1-like | Up | - | Down |
| P16858 | Glyceraldehyde-3-phosphate dehydrogenase | - | - | Down |
| P17156 | Heat shock-related 70 kDa protein 2 | Up | - | Down |
| P18653 | Ribosomal protein S6 kinase alpha-1 | Up | - | Up |
| P18654 | Ribosomal protein S6 kinase alpha-3 | Up | - | Up |
| P19091 | Androgen receptor | - | UP | Down |
| P20029 | Endoplasmic reticulum chaperone BiP | Up | Down | Down |
| P27661 | Histone H2AX | Up | Down | Down |
| P38647 | Stress-70 protein, mitochondria | - | Down | Down |
| P47962 | 60S ribosomal protein L5 | - | - | Down |
| P50149 | Guanine nucleotide-binding protein G(t) subunit alpha-2 | Up | - | Up |
| P60710 | Actin, cytoplasmic 1 | - | - | Down |
| P61982 | 14-3-3 protein gamma | Up | - | Down |
| P62259 | 14-3-3 protein épsilon | Up | - | Down |
| P62737 | Actin, aortic smooth muscle | Up | Up | - |
| P62908 | 40S ribosomal protein S3 | - | - | Down |
| P63017 | Heat shock cognate 71 kDa protein | - | - | Down |
| P63038 | 60 kDa heat shock protein, mitochondrial | Down | Down | - |
| P63101 | 14-3-3 protein zeta/delta | - | UP | Down |
| P63260 | Actin, cytoplasmic 2 | - | - | Down |
| P68033 | Actin, alpha cardiac muscle 1 | Up | Up | Down |
| P68040 | Receptor of activated protein C kinase 1 | Up | - | Down |
| P68134 | Actin, alpha skeletal muscle | Up | Up | Down |
| P68368 | Tubulin alpha-4A chain | - | Down | Down |
| P68369 | Tubulin alpha-1A chain | Up | Down | Down |
| P68372 | Tubulin beta-4B chain | - | Down | Down |
| P68510 | 14-3-3 protein eta | Up | - | Down |
| P80315 | T-complex protein 1 subunit delta | Down | Down | Down |
| P80316 | T-complex protein 1 subunit epsilon | Up | Up | - |
| P84078 | ADP-ribosylation factor 1 | Up | Down | Down |
| P97477 | Aurora kinase A | - | UP | Down |
| P99024 | Tubulin beta-5 chain | Up | Down | Down |
| Q01853 | Transitional endoplasmic reticulum ATPase | Up | Down | Down |
| Q02248 | Catenin beta-1 | Down | Down | - |
| Q02257 | Junction plakoglobin | Up | - | - |
| Q02566 | Myosin-6 | Down | Down | - |
| Q3TKT4 | Transcription activator BRG1 | - | UP | Down |
| Q3V312 | Receptor-type tyrosine-protein phosphatase U | Up | Up | - |
| Q5SX39 | Myosin-4 | Down | Down | Down |
| Q5SX40 | Myosin-1 | Down | Down | - |
| Q61188 | Histone-lysine N-methyltransferase EZH2 | Up | - | Up |
| Q61696 | Heat shock 70 kDa protein 1A | Up | - | Down |
| Q61699 | Heat shock protein 105 kDa | - | Down | - |
| Q61879 | Myosin-10 | Up | - | Down |
| Q64511 | DNA topoisomerase 2-beta | Down | Down | - |
| Q6P5F9 | Exportin-1 | Down | Down | - |
| Q6P5G0 | Mitogen-activated protein kinase 4 | - | UP | Down |
| Q6URW6 | Myosin-14 | Up | Up | - |
| Q7TMM9 | Tubulin beta-2A chain | Up | Down | Down |
| Q80Y86 | Mitogen-activated protein kinase 15 | Up | Up | Down |
| Q8BFZ3 | Beta-actin-like protein 2 | Up | - | Down |
| Q8BG05 | Heterogeneous nuclear ribonucleoprotein A3 | Up | Down | Down |
| Q8BM72 | Heat shock 70 kDa protein 13 | Down | - | Down |
| Q8VDD5 | Myosin-9 | Up | - | Down |
| Q91VC3 | Eukaryotic initiation factor 4A-III | - | - | Down |
| Q91Z83 | Myosin-7 | Down | Down | - |
| Q922F4 | Tubulin beta-6 chain | Up | Down | Down |
| Q99NB8 | Ubiquilin-4 | Up | - | Up |
| Q9CQN1 | Heat shock protein 75 kDa, mitochondrial | - | - | Down |
| Q9CQV8 | 14-3-3 protein beta/alpha | - | UP | Down |
| Q9CTN4 | Rho-related BTB domain-containing protein 3 | Up | Up | - |
| Q9CWF2 | Tubulin beta-2B chain | Up | Down | Down |
| Q9D6F9 | Tubulin beta-4A chain | Down | Down | Down |
| Q9ERD7 | Tubulin beta-3 chain | - | Down | Down |
| Q9JI91 | Alpha-actinin-2 | Up | - | - |
| Q9WUT3 | Ribosomal protein S6 kinase alpha-2 | Up | - | Up |
| Q9Z1M4 | Ribosomal protein S6 kinase beta-2 | - | Up | Down |
